# Phase Transition of a Disordered Nuage Protein Generates Environmentally Responsive Membraneless Organelles

**DOI:** 10.1016/j.molcel.2015.01.013

**Published:** 2015-03-05

**Authors:** Timothy J. Nott, Evangelia Petsalaki, Patrick Farber, Dylan Jervis, Eden Fussner, Anne Plochowietz, Timothy D. Craggs, David P. Bazett-Jones, Tony Pawson, Julie D. Forman-Kay, Andrew J. Baldwin

**Affiliations:** 1 Lunenfeld-Tanenbaum Research Institute, Mount Sinai Hospital, Toronto, ON M5G 1X5, Canada; 2 Physical and Theoretical Chemistry Laboratory, University of Oxford, Oxford OX1 3QZ, UK; 3 Research Institute, Hospital for Sick Children, 686 Bay Street, Toronto, ON M5G 0A4, Canada; 4 Department of Physics, University of Toronto, 60 St. George Street, Toronto, ON M5S 1A7, Canada; 5 Clarendon Laboratory, University of Oxford, Oxford OX1 3PU, UK; 6 Department of Biochemistry, University of Toronto, 1 King's College Circle, Toronto, ON M5S 1A8, Canada

## Abstract

Cells chemically isolate molecules in compartments to both facilitate and regulate their interactions. In addition to membrane-encapsulated compartments, cells can form proteinaceous and membraneless organelles, including nucleoli, Cajal and PML bodies, and stress granules. The principles that determine when and why these structures form have remained elusive. Here, we demonstrate that the disordered tails of Ddx4, a primary constituent of nuage or germ granules, form phase-separated organelles both in live cells and in vitro. These bodies are stabilized by patterned electrostatic interactions that are highly sensitive to temperature, ionic strength, arginine methylation, and splicing. Sequence determinants are used to identify proteins found in both membraneless organelles and cell adhesion. Moreover, the bodies provide an alternative solvent environment that can concentrate single-stranded DNA but largely exclude double-stranded DNA. We propose that phase separation of disordered proteins containing weakly interacting blocks is a general mechanism for forming regulated, membraneless organelles.

## Highlights

• Intrinsically disordered N terminus of Ddx4 forms organelles in cells and in vitro
• Phase transition to form organelles is driven by electrostatic interactions
• Methylation, ionic strength, and temperature changes can dissolve the organelles
• Sequence determinants of formation are common in membraneless organelle proteins

## Graphical Abstract

**Figure:**
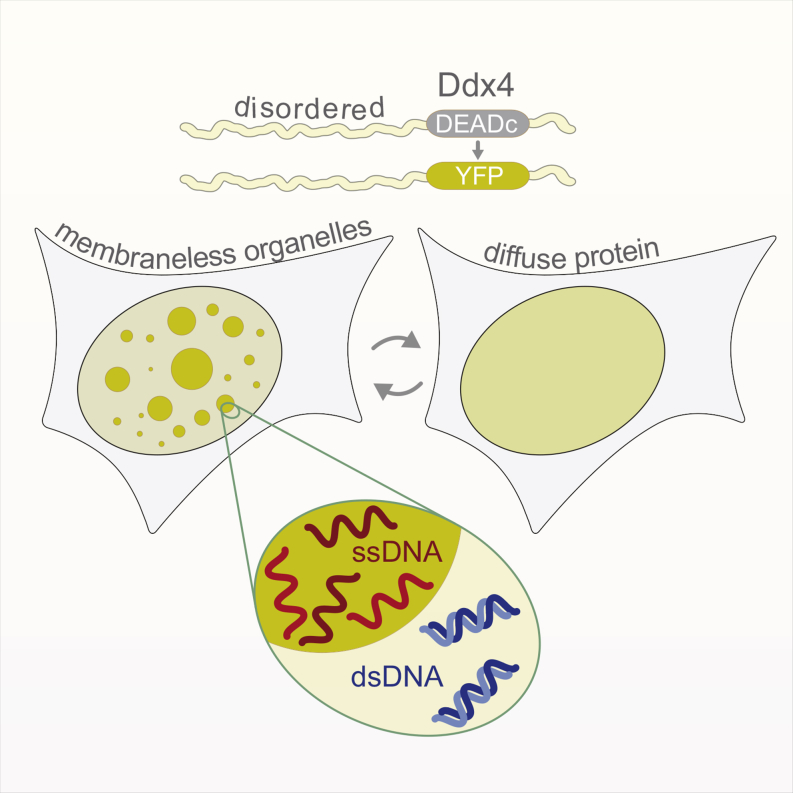

## Teaser

Nott et al. demonstrate that a single protein constituent can reversibly form membraneless organelles both in vitro and in cells. The bodies provide an alternative solvent environment that concentrates single-stranded, but exclude double-stranded DNA. We propose that phase separation of disordered proteins is a general mechanism for forming regulated, membraneless organelles.

## Introduction

Biochemical reactions in the cell frequently have mutually exclusive solution requirements, leading to a need to keep them spatially separated. Membrane encapsulation is a commonly used strategy in roles ranging from controlling the flow of genetic information, via the nucleus and the ER, to maintaining the isolated acidic environment within a lysosome. An alternative strategy involves the formation of membraneless, proteinaceous organelles, including the prominent nucleolus (Montgomery, 1898), PML bodies (de Thé et al., 1990; Melnick and Licht, 1999), Cajal bodies (Cajal, 1903) and nuclear speckles (Cajal, 1910) in the nucleus, and P bodies and both stress and germ granules in the cytoplasm. These cellular structures have been described as coacervates (Hyman and Brangwynne, 2011; Wilson, 1899) and are optically resolvable as spherical micron-sized droplets. The absence of a surrounding membrane enables these organelles to rapidly assemble or dissolve following changes in the cell’s environment and in response to intracellular signals, critical for cellular integrity and homeostasis (Dundr and Misteli, 2010) (Figure S1).

A striking feature of membraneless organelles is that their largely proteinaceous interior partially excludes the bulk aqueous phase (Brangwynne, 2011; Hyman and Brangwynne, 2011). Such organelles behave as liquid droplets. Fluorescence recovery after photobleaching (FRAP) experiments interrogating organelles such as the nucleolus and Cajal bodies indicate that their constituent molecules internally diffuse rapidly (Phair and Misteli, 2000), and P-granules, the worm analog of mammalian nuage or germ granules, condense from a pool of diffuse constituents following specific biological cues (Brangwynne et al., 2009). Moreover, spherical nucleoli of the amphibian oocyte have been observed to coalesce when in close contact and show a size distribution that obeys a simple power law, indicating the formation of liquid droplets (Brangwynne et al., 2011). On a residue level, sequences of low complexity, such as repeated RG, QN, and YG repeats, are important for forming RNA granules, stress granules and P bodies (Decker et al., 2007; Kato et al., 2012; Sun et al., 2011). An understanding of the interactions that stabilize such structures and regulate their biogenesis, as well as a rationale for their biochemical function, has remained elusive.

**Figure 1:**
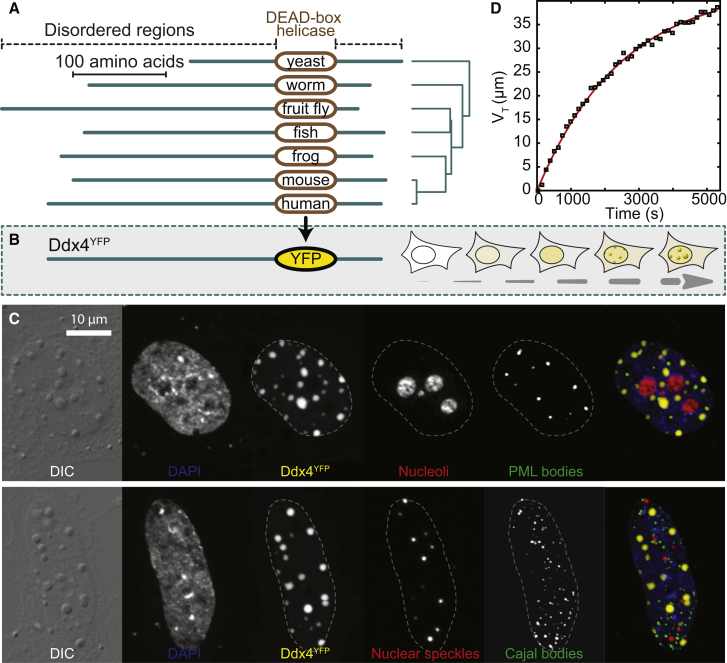
Ddx4 Spontaneously Self-Assembles to Form Organelles in Live Cells (A) Evolutionary relationships between the disordered regions of Ddx4 homologs and their domain architectures. Disordered regions (green) and locations of DEAD-box helicase domains (brown) are indicated. (B) Schematic showing the DEAD-box helicase domain of Ddx4 replaced with YFP before being transfected into HeLa cells. Ddx4^YFP^ organelles appear over time. (C) Differential interference contrast (DIC) and corresponding extended focus fluorescence intensity images of a HeLa cell expressing Ddx4^YFP^. Ddx4^YFP^ forms dense, spherical organelles in the nucleus. Cells were stained with antibodies to visualize nucleoli, PML bodies, nuclear speckles, and Cajal bodies as indicated, revealing that Ddx4 organelles are entirely distinct from these other bodies. (D) The variation in total droplet volume with time is explained by the Avrami equation for nucleated growth (Supplemental Experimental Procedures Section 5). The time is measured from the appearance of the first droplet.

To address these questions, we have studied a dominant protein constituent of a membraneless organelle as a model. Ddx4 proteins are essential for the assembly and maintenance of the related nuage in mammals, P-granules in worms, and pole plasm and polar granules in flies (Liang et al., 1994). This epigenetically crucial nuage/chromatoid body (CB) family of membraneless organelles hosts components of an RNAi pathway, guarding spermatocytes and spermatids against the deleterious activity of transposable elements (Kotaja and Sassone-Corsi, 2007). Typical of non-membrane encapsulated organelles, nuages are generally spherical and dynamically change in number, size, and composition over their lifecycle (Meikar et al., 2011), appearing first in the juxtanuclear cytoplasm of early spermatocytes, moving toward the base of the flagellum during spermatogenesis before finally dispersing. A primary constituent of nuage is Ddx4 (Kotaja et al., 2006). In addition to a central DEAD-box RNA helicase domain that uses ATP to unwind short RNA duplexes, Ddx4 has extended N and C termini that are predicted to be intrinsically disordered (Figures 1A and S2) (Forman-Kay and Mittag, 2013).

Here, we demonstrate that human Ddx4 and its isolated disordered N terminus spontaneously self-associate both in cells and in vitro into structures that are indistinguishable from the cellular Ddx4-organelles. The mechanism for this is a phase separation commonly encountered in polymer chemistry, and the interactions that hold the organelles together are primarily electrostatic in origin. Moreover, arginine methylation, alternative splicing, and changes in ionic strength and temperature under near-physiological conditions readily dissolve the Ddx4 bodies. Two highly conserved features in the sequence of Ddx4 that enable droplet formation are identified: repeating 8–10 residue blocks of alternating net charge and an over-representation of FG, GF, RG, and GR motifs within the positively charged blocks. These features are found to occur in a significant number of intrinsically disordered proteins associated with membraneless organelles. The interior of the organelles concentrate single-stranded DNA, yet largely exclude double-stranded DNA, suggesting that the bodies play a role in localizing nucleic acids. These findings lend insights into the role of intrinsically disordered proteins in the regulated spontaneous self-assembly of cellular membraneless organelles.

## Results

### The Intrinsically Disordered Termini of Ddx4 Condense in the Nucleus of HeLa Cells to Form Organelles

In order to follow the Ddx4 disordered regions within a cell, we generated a mimic, Ddx4^YFP^, in which the DEAD-box helicase is substituted by YFP, a fluorescent protein of similar dimensions and overall charge to the helicase domain (Figure 1A). This protein was transfected into HeLa cells, and both its expression and localization were monitored using fluorescence microscopy (Figures 1B and S2). At low expression levels, Ddx4^YFP^ was diffuse in both the nucleus and cytoplasm. As the intra-cellular concentration increased over time, dense micron-sized spherical bodies were observed to form in the nucleus that were similar in appearance to nuclear foci but physically distinct from other membraneless organelles such as the nucleoli (Figure 1C). A Ddx4 construct containing both YFP and the DEAD-box helicase domain similarly formed organelles, although they were observed to form in the cytoplasm (Figure S2C). The Ddx4^YFP^ bodies have the appearance and behavior of organelles by optical microscopy and can therefore be classified as such.

The maturation of Ddx4^YFP^ organelles was followed using time-lapsed live-cell imaging, where foci corresponding to spherical organelles of radius 0.1–1 μm could be readily identified (Movie S1). Rather than precipitating from solution in many locations at once, organelles were observed to appear individually. The growth of individual organelles within a single cell monitored over time conforms exceptionally well to that expected for Avrami nucleated particle growth (Fanfoni and Tomellini, 1998) (Figure 1D), consistent with the interpretation of the phenomenon as a phase separation involving condensation of Ddx4^YFP^ monomers into proteinaceous organelles. A more detailed analysis suggests that the number of droplets and their sizes are limited by the quantity of free monomer, a property that in principle can be closely regulated (Figure S3).

### Ddx4^YFP^ Organelles Have a Dynamic Liquid-like Structure and Respond Rapidly to Changing Solution Conditions

**Figure 2:**
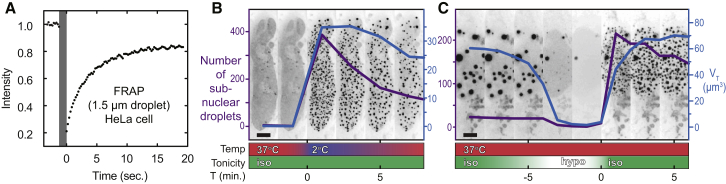
Ddx4^YFP^ Organelles Are Internally Mobile and Respond Rapidly to Changes in Environmental Temperature and Tonicity (A) Fluorescence recovery after photobleaching (FRAP) of a Ddx4^YFP^ organelle in a live HeLa cell at 37°C. Sample bleaching is indicated with a gray bar. 50% of the fluorescence signal is recovered within approximately 2.5 s post-bleach, corresponding to a diffusion coefficient of 3 ± 1 × 10^−13^ m^2^ s^−1^. (B) Cold shock induces condensation of sub-nuclear Ddx4^YFP^ droplets at low expression levels. Extended focus fluorescence intensity images showing the nucleus from a time series analysis of a HeLa cell expressing Ddx4^YFP^ undergoing cold shock. Images are shown at 2-min intervals. Prior to cold shock treatment, Ddx4^YFP^ had not reached the critical concentration for phase separation at 37°C and was diffuse in the nucleoplasm (first two frames). Rapid exchange of growth media at 37°C for media cooled on ice (time = 0) induced small Ddx4^YFP^ droplets to condense rapidly within the nucleus (purple line, number of droplets; blue line, total volume of droplets). Following cold shock, the number of Ddx4^YFP^ droplets decreased through a combination of coalescence and dissolution as the temperature rose. Scale bar, 5 μm (see Movie S2). (C) Extended focus fluorescence intensity image slices showing a section of the nucleus from a time series analysis of a HeLa cell containing Ddx4^YFP^ droplets undergoing osmotic shock. Images are shown at 2-min intervals. Axis labels, data colors, and scale as in (B). See Movies S3 and S4.

The internal order within the organelles was assessed using fluorescence recovery after photobleaching (FRAP) measurements. The half time to recovery of the fluorescence signal of a photo-bleached body of diameter 1.5 μm took approximately 2.5 s at 37°C (Figure 2A), corresponding to an approximate diffusion coefficient of 3 ± 1 × 10^−13^ m^2^ s^−1^, a value two orders of magnitude lower than that measured for free globular proteins of a similar size as determined by both FRAP and NMR (Figure S5B). These self-diffusion rates are consistent with those of other non-membrane organelles, such as nuclear speckles and nucleoli (Phair and Misteli 2000). While the observed diffusion within the droplets is substantially slower than the motion of free protein, the interior is nevertheless highly mobile, consistent with weak interactions between Ddx4 proteins within the droplet.

To assess the internal structure of in situ Ddx4^YFP^ organelles and to determine if they contain, for example, fibrillar substructure, we employed electron spectroscopic imaging (ESI). This technique enables the visualization of nitrogen and phosphorous structures at the approximate resolution of 30 atoms per pixel (60 Å × 60 Å) without the use of contrast-enhancing reagents required for conventional TEM experiments. At this resolution, there was no significant variation in density through the organelles, as would be expected if the structure were based on amyloid fibrils (Kato et al., 2012; Kim et al., 2013). The organelles are clearly partitioned from surrounding nuclear structures including chromatin domains and other native nuclear bodies (Figures 1C and S2B), a finding consistent with the liquid droplet-like nature suggested by FRAP measurements (Figure 2A).

Ddx4^YFP^ organelles were exposed to cellular environments varying in both temperature and tonicity to elucidate factors that dictate their stability. Cells transfected with Ddx4^YFP^ at low expression levels where no organelles were observed were subjected to cold shock by rapidly transferring them from 37°C to 2°C. This caused immediate condensation of Ddx4^YFP^ organelles (Figure 2B and Movie S2). As the temperature of the cells was subsequently allowed to rise, the number (purple) and total volume (blue) of organelles decreased as Ddx4^YFP^ protein dissociated from organelles and dispersed into the nucleoplasm. Similarly osmotic shock, induced by a rapid change from isotonic (150 mM ionic strength) to hypotonic conditions (∼150 μM ionic strength) caused the rapid dissolution of Ddx4^YFP^ droplets. These immediately reformed upon return to isotonic conditions (Figure 2C and Movies S3 and S4). These data reveal that the disordered termini of Ddx4 mediate self-organization into macroscopic fluxional spherical structures in live cells that can be classified as organelles, which can respond rapidly to changes in the cellular environment.

### Ddx4 Disordered Regions Reversibly Form Structures In Vitro that Are Indistinguishable from Those Formed in Cells

**Figure 3:**
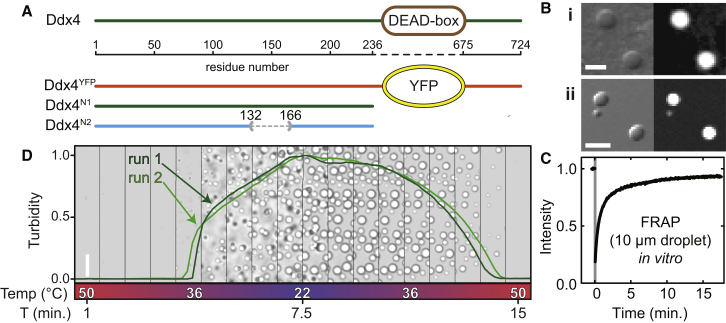
The N Terminus of Ddx4 Reversibly Forms Organelles In Vitro (A) Schematic showing the relationship between constructs of Ddx4 and the wild-type protein. Ddx4^N1^ (residues 1–236) and Ddx4^N2^ contain only the disordered N terminus. (B) DIC (left) and YFP fluorescence (right) images of (i) Ddx4^YFP^ organelles inside HeLa cells (scale bar, 2 μm) and (ii) 60:1 Ddx4^N1^:Ddx4^YFP^ organelles formed in vitro at 150 mM NaCl (scale bar, 10 μm). (C) FRAP curve of a 10 μm diameter droplet containing Ddx4^N1^ and recombinant, purified Ddx4^YFP^ at a molar ratio of 60:1 in 150 mM NaCl buffer at 20°C. The bleach period is indicated with the gray bar. 50% of the fluorescence signal is recovered after approximately 1 min, corresponding to a diffusion coefficient of 4 ± 1 × 10^−13^ m^2^ s^−1^. (D) Time series analysis of bright-field microscopy images of Ddx4^N1^ (202 μM protein, 200 mM NaCl) with varying temperature, shown at 50 s intervals (scale bar, 50 μm). At 50°C, the sample was monophasic with low turbidity. Temperature was linearly decreased (4°C min^−1^) from 50°C to 22°C. At 36°C, the turbidity of the sample rapidly increased concomitant with the emergence of an incipient dense phase containing concentrated Ddx4^N1^. After holding at 22°C for 1 min, the sample was reheated to 50°C. At approximately 45°C during reheating, the condensed phase was completely dissolved and the turbidity of the solution returned to its initial turbidity. The thermal cycle was repeated with the same sample in situ (light green line), revealing that the changes in the droplet are fully reversible.

To gain insight into the transitions observed in cells, we expressed and purified Ddx4^YFP^ recombinantly. In addition, two further polypeptides were also produced, corresponding to the N-terminal disordered region (residues 1–236), Ddx4^N1^, and residues 132–166 exchanged for a single aspartate, Ddx4^N2^, corresponding to a naturally occurring splice variant (Figure 3A). The properties of the dispersed Ddx4^N1^ and Ddx4^N2^ were interrogated using solution-state NMR. The narrow range of amide proton chemical shifts observed in a ^1^H/^15^N HSQC spectra of the two proteins (Figure S5A) confirmed that they are intrinsically disordered. The hydrodynamic radii (R_h_) of the constructs, determined using pulsed field gradient NMR experiments, were found to fall between 29 and 33 Å (6.3–7.1 × 10^−10^ m^2^s^−1^ Figure S5B). This size is closer to that predicted for a folded protein of this length (23 Å) rather than an unfolded protein (50 Å) of this length (Marsh and Forman-Kay, 2010; Wilkins et al., 1999), indicating that the Ddx4 N-terminal intrinsically disordered region maintains transient tertiary contacts that keep the protein compact (Dedmon et al., 2005).

Under near-physiological conditions of ionic strength 150 mM, 37°C, solutions of 100 μM Ddx4^N1^ and Ddx4^YFP^ rapidly became turbid. When droplets in the turbid phase were imaged, their morphologies and (qualitatively) their distribution of particle size and time dependence mirrored that seen within cells, indicating that we can readily recapitulate the organelles in vitro (Figure 3B). By contrast, under identical conditions, the splicing variant Ddx4^N2^ remained soluble, revealing that alternative splicing can regulate the formation of organelles.

As the droplets formed in vitro appeared identical in form to those observed in cells, FRAP experiments were performed to determine if they also have similar physical characteristics and internal structure. Using Ddx4^YFP^ as a tracer in droplets otherwise composed of Ddx4^N1^ (molar ratio of 1:60), 50% of fluorescence signal intensity was recovered after approximately 1 min following photobleaching of a 10 μm diameter dense-phase droplet (Figure 3C) corresponding to a diffusion coefficient of 4 ± 1 × 10^−13^ m^2^ s^−1^. Within experimental uncertainty, this is identical to the value observed directly within live cells (3 ± 1 × 10^−13^ m^2^ s^−1^; Figure 2A), indicating that the internal structure and internal dynamics of the organelles formed both in vivo and in vitro are highly similar.

Since Ddx4^YFP^ organelles could be induced in cells by cold shock (Figure 2B), the in vitro organelles were subject to thermal perturbation. Using bright-field microscopy and a thermal stage, a fully dispersed solution of Ddx4^N1^ (pH 8.0) at 50°C was cooled at 4°C min^−1^ to 22°C. At 36°C, the solution became turbid and droplets were observed to condense (Figure 3D). After equilibration at 22°C for 1 min, the sample was reheated to 50°C. As the temperature was raised, the droplets were observed to dissolve. Multiple cycles were repeated, revealing that the process is reversible (Figure 3D). In both respects, the thermal cycle was highly similar to that observed within cells.

### Ddx4^N1^ Organelles Are Stabilized Predominantly by Electrostatic Interactions

**Figure 4:**
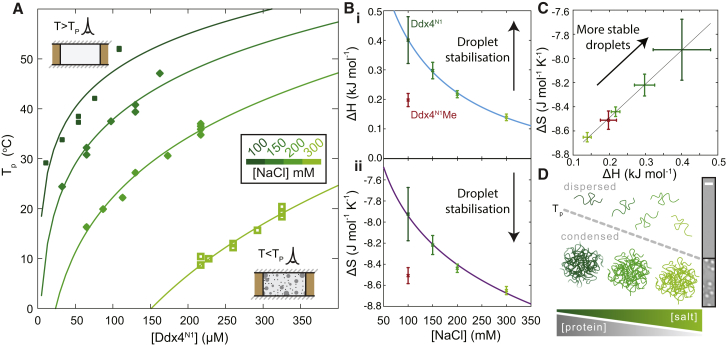
Quantitative Analysis and Interpretation of the Ddx4^N1^ Phase Transition (A) The temperature at which the phase transition is observed, T_P_, was determined as a function of protein concentration and ionic strength at pH 8. At a given ionic strength, the Flory-Huggins model of polymer phase separation quantitatively describes each curve. This yields two fitting parameters, the enthalpy and entropy changes of the transition, which report on the microscopic interactions between molecules. (B) The interaction parameters varied in a predictable way with increasing salt. The enthalpic contribution to the interaction parameter (i) was found to decrease as a function of increasing NaCl. This is quantitatively explained by fitting the curve to a screened coulomb potential (light blue, Equation S19). The non-ionic component of the enthalpy is close to zero, −0.058 ± 0.137 kJ mol^−1^, the relative permittivity within the condense phase was 45 ± 13, and the average spacing between opposite charges is 13 ± 2 Å. The entropic contribution to the interaction parameter (ii) decreases slightly with increasing salt, fitted to Equation S20. The error bars represent the SE in the fitted parameters (Figure 4A). (C) The entropy and enthalpy values are correlated, suggesting that when the interactions are destabilized at higher salt, the chains in the interior of the droplet become more mobile. The error bars represent the SE in the fitted parameters (Figure 4A). (D) Schematic representation of dissolution of the Ddx4 condensed phase and expansion of the monomer in the disperse phase through increasing ionic strength or temperature. Ddx4^N1^ protein chains depicted as green lines. Transition point (T_p_) is indicated with a dashed gray line. The ionic interactions within the droplets are attenuated with increasing salt, as is the residual structure within the protein in the dispersed phase. Corresponding bright-field images are shown on the right. Scale bar, 10 μm.

In the case where molecular chains attract each other, polymer theory anticipates that, at high concentrations and low temperatures, they will phase separate and form condensed droplets suspended in solvent. By contrast, at high temperature, the translational entropy of the free polymer will dominate and the polymer will mix with solvent. As the temperature is lowered, a “bimodal” or “cloud point” is reached, T_P_, where favorable interactions overcome the translational entropy loss, and droplets of pure polymer will condense via a nucleated mechanism, as quantitatively described by Flory-Huggins theory of phase separation (Flory, 1942; Huggins, 1942). We measured T_P_ for Ddx4^N1^ (Figure S4) as a function of protein concentration and ionic strength, enabling the construction of a phase diagram (Figure 4A, points). At all ionic strengths examined, T_P_ increases with increasing Ddx4^N1^ concentration in a manner that is well predicted by Flory-Huggins theory (Figure 4A, solid lines). The transition temperatures were found to decrease as ionic strength was increased, indicating that the interactions between the protein molecules within the condensed phase have a strong electrostatic component. Flory-Huggins theory (Flory, 1942; Huggins, 1942) was found to quantitatively explain the scaling of T_P_ with increasing protein concentration, enabling characterization of how the enthalpic and entropic interaction terms vary with ionic strength (Figure 4A).

The scaling of the enthalpic part of the free energy can be quantitatively explained by assuming that the individual interactions between protein molecules within the droplet can be represented by a screened coulombic (electrostatic) potential (Shammas et al., 2011) such that increasing salt predictably attenuates the interaction (Equation S19). Fitting this model to the data (Figure 4Bi) yields an average separation between interacting charges of 13 ± 2 Å and the relative permittivity, or dielectric, of the droplet of 45 ± 13. This value is smaller than that of bulk water (80) (Figure 4Bi), suggesting that electrostatic interactions will be less screened than in bulk water. It is interesting to compare this value to those of the hydrophobic interior of folded proteins (4), of the interior of a lipid bilayer (2–4), and of polar organic solvents such as acetonitrile (38) and DMSO (47). The favorable interactions are effectively entirely screened at high salt, indicating that electrostatic forces are responsible for droplet stability.

The entropic part of the free energy was found to favor droplet formation under all conditions, suggesting that the residual order in either the dispersed protein or its associated solvent molecules is substantially reduced upon condensation into droplets. Both entropy and enthalpy changes are correlated (Figure 4C) tentatively, suggesting that as interactions are weakened with salt the interior becomes more mobile. Taken together, we can conclude that the protein droplets are held together primarily by electrostatic interactions. These findings reveal that the responsiveness of organelles in the cell to changing environmental conditions (Figure 4D) stems from the microscopic interactions between individual protein chains.

### Arginine Methylation Destabilizes Ddx4^N1^ Organelles

**Figure 5:**
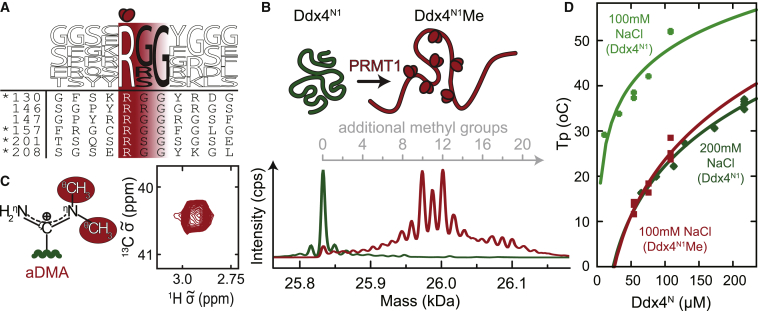
Post-Translational Modification by Arginine Methylation Alters the Phase Transition of Ddx4^N1^ (A) Sequence logo (weblogo.berkeley.edu) depicting the amino acid motifs surrounding arginine residues of Ddx4^N1^ predominantly targeted by PRMT1. Arginine residues to be converted to aDMA are highlighted in dark red and with two small ellipses. The amino acid numbers of the modified arginine residues are shown within their respective sequence contexts. Asterisks highlight aDMA sites identified in Ddx4^N1^Me with 95% probability (Scaffold score) from a combination of trypsin and GluC digestion of recombinant, purified Ddx4^N1^Me. aDMA at sites 146 and 147 was identified at ∼65% probability (Scaffold score). (B) Schematic and mass reconstruction of +TOF MS spectra of Ddx4^N1^ (green; 25.833 kDa) and Ddx4^N1^Me (dark red). In the latter, a series of peaks was observed between 1 and 20 methyl additions. The major peaks indicate complete aDMA modification at 5 and 6 sites, respectively. (C) A schematic of aDMA together with an insert showing the ^1^H-^13^C HSQC NMR spectrum of the ^θ^CH_3_ of Ddx4^N1^Me. The chemical shifts of the methyl groups verify that the modification is aDMA (see Figure S5). (D) The phase-transition temperatures of Ddx4^N1^Me (dark red) are shifted compared to the unmodified form under the same conditions (light green). Modification with aDMA at a mixture of 5–6 aDMA sites reduces the transition temperature by 25°C, an effect on the phase transition comparable to increasing the ionic strength by 100 mM.

Ddx4 and other nuage proteins are regulated through the methylation of multiple arginine sites (Chen et al., 2009; Kirino et al., 2010). In the case of the Piwi proteins, arginine methylation generates binding sites for Tudor domain-containing binding partners, thereby co-localizing them with the nuage (Liu et al., 2010). In mammalian cells, the enzyme PRMT1 catalyzes the addition of two methyl groups to one of the guanidine nitrogen atoms of the arginine side chain in predominantly RGG motifs (Wooderchak et al., 2008), converting it to asymmetric dimethyl arginine (aDMA) (Tang et al., 2000) (Figure S5E). Ddx4^N1^ is post-translationally modified at multiple sites by PRMT1 in vivo (Kirino et al., 2010) and contains six predicted methylation sites (Figure 5A). By co-expressing Ddx4^N1^ with PRMT1 in *E. coli*, between 1 and 20 methyl groups were added, with the majority of chains containing 10 and 12 additional methyl groups (Figure 5B). Solution-state NMR confirmed that the dominant modification was aDMA (Figures 5C and S5), and the locations of the sites were confirmed by proteolytic cleavage and fragmentation mass spectrometry. Taken together, the majority of protein molecules had either 5 or 6 aDMA-modified arginine residues in the predicted sites (Figure S5). Remarkably, methylation of this type significantly destabilized the droplets, lowering the transition temperature by 25°C (Figure 5D). The extent of the destabilization of the droplets is the equivalent of adding 100 mM of additional salt to Ddx4^N1^. Post-translational modification is therefore revealed to be a mechanism through which droplet formation can be attenuated under physiological conditions and an effective method to regulate distinct subcellular microenvironments.

### Patterns of Charged Residues Are Required for Organelle Formation

The propensity for polypeptides to spontaneously phase separate under physiological conditions is not a property common to all intrinsically disordered proteins. To identify which features within the sequence confer this ability, we analyzed the disordered tails of orthologous Ddx4 proteins to understand what distinguishes them from other intrinsically disordered proteins. Droplet formation both in cells and in vitro was highly sensitive to ionic strength, indicating that the charged residues confer significant stability to the droplets. While the number of hydrophobic residues is lower in Ddx4 proteins than the values for an “average” intrinsically disordered protein (Figure S6A), the proportion of charged residues in Ddx4 orthologs (25%) is very close to the average number of charged residues in all IDPs surveyed (26.1%), revealing that the predictors are more complex.

**Figure 6:**
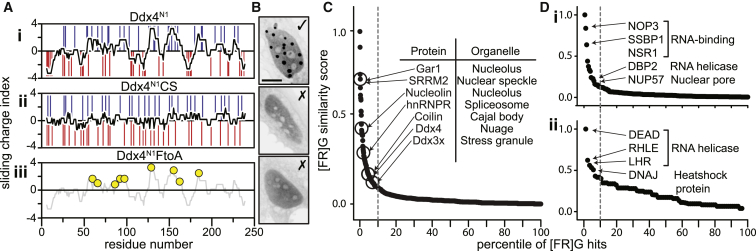
The Sequence Features that Enable Droplet Formation by Ddx4 and Their Distribution within the Human Genome (A) Sliding net charge (10 amino acid window, black) is shown for (i) Ddx4^N1^ and (ii) a charge-scrambled mutant, Ddx4^N1^CS, obtained by swapping the positions of positive residues (blue bars) and negative residues (red bars) to minimize any persistence of blocks of charge. (iii) A mutant where nine phenylalanine residues, whose placement was highly conserved, were mutated to alanine (Ddx4^N1^FtoA, see Figure S6). The positions of the nine phenylalanine residues (yellow circles) mutated to alanine are indicated. (B) Representative fluorescence images from cell imaging experiments reveal that Ddx4^N1^CS and Ddx4^N1^FtoA do not form organelles in cells under physiological conditions. Residual HeLa nucleoli are still observed as fluorescence-depleted regions within the cell nucleus. (C) The human genome was surveyed for sequences with similar physical properties to the Ddx4 disordered termini. 1,556 sequences out of 14,198 were identified to have [F/R]G spacings in their sequence that are similar to the Ddx4 ortholog family. The top 10% of these are indicated (dotted line). A significant number of proteins associated with forming non-membrane organelles were present in this group. (D) Similar plots from the yeast (i) and *E. coli* (ii) genomes revealing a number of proteins closely associated with nucleic acid biochemistry.

A notable feature of Ddx4 disordered termini is that they arrange their charged residues into clustered blocks of net positive and negative charge (Figure 6Ai), resembling a block co-polymer. The clusters persist for approximately 8–10 residues in length and tend to contain 3–8 similarly charged residues. To determine the physical importance of this charge patterning, we produced a Ddx4 variant, Ddx4^N1^CS, with the same overall net charge, but in which the blocks were scrambled (Figure S6G). In Ddx4^N1^CS, the regions of opposing charge are removed while simultaneously maintaining the same overall isoelectric point (PI), amino acid composition, and positions of all other residues (Figure 6Aii). This construct was unable to form organelles in vitro under near-physiological conditions. When expressed in cells in the charge-scrambled form of Ddx4^YFP^, the protein accumulated to high concentrations without forming organelle-like structures (Figure 6Bii), revealing the importance of charge patterning in organelle formation.

### Repeated FG and RG Spacing in Ddx4 N Termini Suggest Cation-Pi Interactions Contribute to Droplet Stability

To ascertain specific sequence features that contribute to droplet stability, we looked for over-representation of amino acid pairs in the disordered regions of Ddx4 orthologs when compared to their background proteomes (Supplemental Experimental Procedures). We found that both GF and FG groupings were both significantly over-represented in human Ddx4 and a common feature of the Ddx4 family of orthologs (Figure S6B, dot size and color, respectively). Closer inspection of the linear sequences of Ddx4 proteins revealed that FG and GF motifs were clustered within positively charged blocks, typically close to arginine residues in the form of either RG or GR dipeptides. We undertook an analysis to determine the relative locations of these residues within the Ddx4 disordered termini to ascertain whether the spacing between these repeats was statistically significant. To conduct this analysis, we first measured the sequence distance between all F-F, R-F, F-R, and R-R residues within the Ddx4 ortholog families, including only F/R residues that are immediately followed or preceded by a G. For example, FGxxxxGR would be recorded as an F-R spacing of 7 residues (Figure S6C). The counts were then normalized to those of the background sequences to ensure that any occurrence is a significant property of the Ddx4 family and not an intrinsic property of disordered proteins. Strikingly, the test revealed a significant statistical trend for FG and GF pairs to be spaced by 8–11 residues apart in Ddx4 disordered termini and RG and GR pairs to be spaced 4 residues apart (Figure S6C). Similar patterns were observed in the F-R distances. When the analysis was extended to include groups of three or more dipeptide repeats, similar, but more pronounced, trends were observed in the spacing of multiple [F/R]G dipeptides (Figure S6D). Taken together, it would appear that there has been evolutionary pressure acting on Ddx4 that holds the relative spacing of [F/R]G pairs within a well-defined window.

Of the 14 F residues within the sequence, this method identified ten within Ddx4^N1^ as having their relative positions conserved by evolution with respect to R and other F residues. Of these, nine were present within positively charged blocks (Figure 6Aiii). To test the physical significance of these residues to droplet formation, a construct was produced where these nine residues were mutated to alanine (Ddx4^N1^FtoA; Figure 6Aiii). Notably, Ddx4^N1^FtoA was unable to induce droplet formation either in cells or in vitro (Figure 6Biii). Finally, the strength of the quadrupole in the aromatic ring of the phenylalanine residues was reduced by enrichment with 3-fluorophenylalanine, Ddx4^N1^F (Figure S6Fi). The organelles were significantly destabilized (Figure S6Fii), confirming the importance of these aromatic residues and suggesting that cation-pi interactions are required for organelle formation.

### Sequence Determinants of Ddx4 Droplet Formation Are Found in Other Organelle-Forming Proteins

The statistical map of FG and RG proximities can be considered a “fingerprint” of organelle-forming features of Ddx4 (Figure S6D). Using this as a reference, we interrogated the human proteome (Yaffe et al., 2001), identifying 1,566 similar sequences (Figure 6C). After ranking the scores of the sequences, we identified a sharp increase in the scoring function that occurred for the top 10%. Interestingly, this group of 156 sequences included a number known to be primary constituents of non-membrane encapsulated organelles, such as Nucleolin and Gar1 (nucleolus), Coilin (Cajal body), hnRNPs (splicing speckles), and Ddx3x (stress granules) (Figure 6C). These results strongly suggest that the sequence properties identified in Ddx4 enabling organelle formation via a phase separation mechanism are general features of organelle-forming proteins. Moreover, similar patterns were observed in a subset of proteins associated with RNA processing in both yeast and *E. coli* genomes, a function commonly localized to membraneless organelles (Figure 6D).

We performed an analysis of the gene ontology (GO) terms in the UniProt database for the top 10% of our ranked sequences. Significantly, these sequences were found to be generally localized in membraneless cellular compartments, such as nuclear bodies, nuclear speckles, the spliceosome, and nucleolus, and involved in associated biological processes, such as RNA processing, chromatin organization, and methylation in both human and yeast genomes (Figure S6E). Moreover, there were a large number of proteins related to cell adhesion found in the extracellular matrix. This observation is remarkable in light of the finding that several of these proteins have been observed to coacervate (Yeo et al., 2011). These results identify a class of intracellular proteins involved in organelle formation and cell-cell adhesion that contain blocks rich in FG/RG repeats and likely form phase-separated structures in vivo.

### Ddx4 Droplets Differentially Solubilize Nucleic Acids

**Figure 7:**
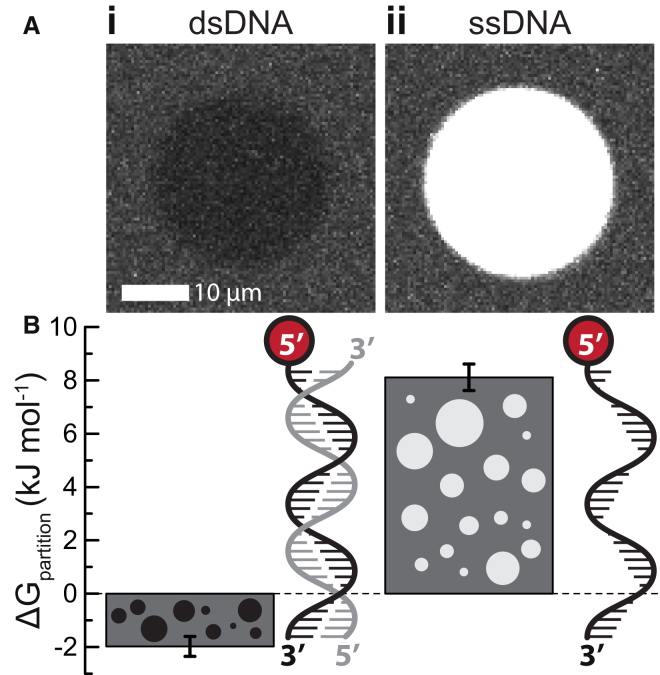
Proteinaceous Organelles Differentially Solubilize Nucleic Acids (A) Ddx4^N1^ organelles were allowed to form under near-physiological conditions at a total concentration of 162.5 μM. (i) Double- and (ii) single-stranded 32-nt DNAs (dsDNA and ssDNA, respectively) tagged with atto647N were added at a concentration of 1 μM. In the case of dsDNA, the majority of the material was excluded from the droplets. The reverse effect was observed for ssDNA. (B) The average and SD (error bar) confocal fluorescence emission intensities from both inside and outside the organelles were used to quantify the partition equilibrium coefficient and its corresponding free energy (Equation 1, Figure S7).

Ddx4-containing nuage and other membraneless organelles are frequently associated with nucleic acid biochemistry. Thus, we might expect them to differentially bind and concentrate various nucleic acids as well as other biomolecules. To test this, we prepared a 32 base single-stranded and 32 base-paired double-stranded DNA labeled with the fluorescent dye atto657N and mixed them with Ddx4 organelles. The relative fluorescence in the two phases indicates the relative concentration, and thus solubility, of the DNA in the two contrasting environments. While the double-stranded DNA was largely excluded from the droplets, the single-stranded DNA was concentrated significantly in the interior of the droplets (Figure 7). This result suggests that the interior of Ddx4 organelles provides a substantially different environment to the aqueous cellular interior in order to preferentially solubilize and concentrate certain types of biomolecules.

## Discussion

### Ddx4 Organelles Are Phase-Separated Droplets

Membraneless organelles such as the nucleolus, nuclear speckles, Cajal bodies, P bodies, and stress granules share many properties (Brangwynne, 2011; Phair and Misteli, 2000). They are resolvable by microscopy, are generally spherical, are internally dynamic, and are composed of a well-defined set of proteins. The disordered N terminus of Ddx4, a primary component of nuage organelles, can self-associate to form bodies indistinguishable from these by optical microscopy, both in cells and in vitro. These condensed droplets have a liquid-like interior, consistent with maintenance of disorder and lack of observed discrete fibrillar or other ordered structure. Their mechanism of formation and thermodynamic properties reveal that these bodies are a condensed phase, distinct from the aqueous background, with the availability of free protein likely determining both the number of organelles and their size distribution through growth kinetics.

Consistent with this picture, droplet formation is readily reversible and responsive to changes in environmental conditions. Increasing the ionic strength or temperature, methylating certain significant arginine residues, and disrupting the charged blocks by alternative splicing can lead to complete dissolution of the droplets. As the functions performed by these organelles are both cell-type specific and temporally regulated, these are means by which the droplets can be regulated in vivo. These organelles are effectively liquid droplets of condensed disordered protein that form an intra-cellular compartment that is distinct from the aqueous background (Brangwynne, 2009, 2011).

### Ddx4 Organelles Are Held Together Primarily by Electrostatic Interactions

Intrinsically disordered proteins experience a higher rate of sequence alteration through evolution than globular proteins (Brown et al., 2011), yet we find two clear sequence features to provide the required electrostatic interactions. Clusters of opposing charge of length 8–10 residues are required for droplet formation together with both FG and RG pairs held in close sequence proximity. It is likely that the F/R residues are engaged in cation-pi interactions, as frequently observed in the context of folded proteins (Gallivan and Dougherty, 1999). Both increased levels of salt and arginine methylation would disrupt quadrupolar interactions, as we observe.

The specific placement of certain residue types has recently been identified as crucial for forming self-assembled structures with desirable physical properties. In the cases of assembly of EWS/FUS proteins, P bodies, and the nuclear pore complex, formation is facilitated by repeated occurrences of the dipeptides YG/RG, QN, and FG, respectively (Balagopal and Parker, 2009; Decker et al., 2007; Frey et al., 2006; Kato et al., 2012; Sun et al., 2011). While individual interactions are relatively weak, careful placement of many such multivalent interactions can lead to the self-assembly of otherwise disordered polypeptide chains (Li et al., 2012).

The Ddx4 sequences are found to conform to a “fingerprint” that consists of FG and RG groups arranged in a distinct pattern. A wider screening of the human, yeast, and *E. coli* genomes reveals that many intrinsically disordered proteins share this pattern, the majority of which are associated with non-membrane encapsulated organelles and extra-cellular adhesion proteins. This indicates that the sequence features we have identified for Ddx4 organelle formation may be more general for formation of membraneless organelles and that prokaryotic organisms may also utilize such proteinaceous bodies for compartmentalization.

### Ddx4 Organelle Formation Is Distinct from Amyloid Formation

It is interesting to compare phase separation of Ddx4 proteins into organelles with the aggregation of proteins more generally into the amyloid fibrils that are associated with misfolding conditions, including Alzheimer’s and Parkinson’s diseases (Dobson, 2003). Their morphologies and internal features easily distinguish the two types of aggregates, as the former is spherical with internal mobility, whereas the latter are fibrillar with constituent monomers that adopt a precise structural arrangement with similar interactions (Baldwin et al., 2011; Fitzpatrick et al., 2013; Tycko, 2011). Both aggregation mechanisms require only that there is favorable free energy for monomer association and both processes are reversible (Baldwin et al., 2011). In the case of amyloid fibril formation, monomers interact predominantly by backbone hydrogen bond interactions between adjacent β sheets, a property likely to be generic to all polypeptide chains (Dobson, 2003). By contrast, the formation of Ddx4 organelles requires patterned electrostatic interactions, suggesting it is not likely to be so widespread. It would be highly undesirable to have uncontrolled protein association into micron-sized bodies and, as for amyloid formation (Monsellier and Chiti, 2007), the majority of the proteome has likely experienced negative selection to remove traces of sequence determinants where they are deleterious.

### Ddx4 Organelles Are Likely Minimally Stable to Enable Regulation by PTMs

The folded structures of proteins have been recognized as being minimally stable, a property that facilitates the relative motion of domains and enables function (Bryngelson et al., 1995). For Ddx4, small changes at the level of individual chains can result in organelle dissolution under physiological conditions, indicating that they too are minimally stable, perhaps to prevent uncontrolled growth. This renders them susceptible to regulation by small perturbations, for example by methylation of arginines by PRMT1, a modification observed in vivo (Chen et al., 2011). Another regulatory mechanism that exploits the finely tuned stability utilizes alternative splicing of Ddx4 to substitute residues 132–166 with a single aspartate, effectively removing an entire positively charged “block” from the sequence and rendering it unable to form organelles under physiological conditions.

Other membraneless organelles have been shown to have similarly finely tuned stabilities under physiological conditions. FUS is induced to self-associate and form granules in the cytoplasm following arginine methylation by PRMT1 (Yamaguchi and Kitajo, 2012). SR proteins enter nuclear speckles for engagement in RNA splicing in the event of phosphorylation, and phosphorylation of coilin is correlated with Cajal body formation (Hearst et al., 2009; Misteli et al., 1998). SUMOylation of PML is required for PML nuclear body formation, and de-SUMOylation allows constituent proteins to be released and bodies to be broken apart during mitosis (Dellaire et al., 2006a, 2006b). Our results together with these observations suggest that PTMs, by affecting both self-association and co-localization with other binding partners, provide a powerful mechanism of dynamic and responsive regulation of organelle formation.

### Ddx4 Organelles Provide an Alternative Phase for Biochemical Processes

By exploiting differential intermolecular interactions between the bulk aqueous solvent and the interior of the droplets, Ddx4 organelles effectively offer a disordered protein phase as an alternative solvent environment for biomolecules. In this case, the organelle phase can largely exclude double-stranded DNA, yet concentrate single-stranded DNA, acting as a molecular filter. It is likely that Ddx4 binds to single-stranded DNA using the same cation-pi interactions that appear to drive self-association, as observed in the context of folded proteins mediating interactions with single-stranded nucleic acids (Gromiha et al., 2004; Morozova et al., 2006). Many membraneless organelles are associated with RNA processing functions, strongly suggesting that this unexpected property of the organelles is functionally relevant. It is a common strategy for an organic chemist to perform reactions in different solvents depending on the reaction required, and so it is interesting to consider a similar process occurring in vivo.

### Conclusion

Here, we demonstrate that intrinsically disordered regions of Ddx4 reversibly phase separate to form droplets both in live cells and in intro via a mechanism encountered frequently in polymer chemistry. These organelles lack an internal structure and are highly fluid, effectively creating a separate solvent from the bulk aqueous environment of the cell, with unique biochemical properties. The interactions that stabilize the droplets are primarily electrostatic in origin and are readily modified by alternative splicing, arginine methylation, changes in ionic strength, and temperature, providing a means for organelle regulation. The sequence characteristics that enable Ddx4 droplet formation are found to be present in a large number of disordered proteins associated with membraneless organelles. Results strongly suggest that phase separation of specific disordered proteins to form organelles is a widespread phenomenon, providing an elegant and dynamically responsive strategy for biological compartmentalization.

## Experimental Procedures

Genes for Ddx4 proteins were synthesized by GenScript and expressed recombinantly in *E. coli*. Methylated Ddx4^N1^, Ddx4^N1^Me, was produced by co-expression with PRMT1. For NMR analysis, isotopically enriched samples were prepared by growing the appropriate sample in M9 media enriched in ^15^N or ^13^C reagents as required (Supplemental Experimental Procedures Section 1). HeLa cells were cultured on MatTek dishes and transfected with Ddx4 variants using the Effectene (QIAGEN) or polyethylenimine (PEI) methods. Cold shock, osmotic shock, and organelle growth experiments were performed on a Leica DMIRE2 inverted microscope equipped with a live cell chamber. Image z stacks recorded at each time point were de-convolved using an appropriate point-spread function, and automated corrections for photobleaching, sample movement, and contrast enhancement were performed using Volocity software. Ddx4^YFP^ organelles were identified by having significant intensity in the microscopic images (>6 SD in pixel intensity more than the background) and were tracked using the Volocity software. The total organelle volume (Figure 1D) was fitted to the Avrami equation (Fanfoni and Tomellini, 1998) for nucleated growth (Supplemental Experimental Procedures Section 5).

FRAP experiments both in vitro and in vivo were performed in a live cell chamber mounted on an Olympus IX81 inverted microscope. The effects of bleaching at 515 nm on the emission intensity at 527 nm were followed, and the recovery of intensity was analyzed using the diffusion equations of Fick (Supplemental Experimental Procedures Section 2).

The transition temperature was measured using a Linkam THMS600 thermal stage mounted on an Olympus BX61 microscope. Sealed sample chambers containing protein solutions comprised coverslips sandwiching a SecureSeal imaging spacer (Sigma) and were mounted on the THMS600 silver heating/cooling block. The variance in the solution conditions was monitored with temperature. The data were analyzed using Flory-Huggins theory as described in the Supplemental Information to obtain the binodal phase temperature, giving estimates for *ΔH* and *ΔS*, the specific enthalpy and entropy changes induced by the interaction (Supplemental Experimental Procedures Section 6).

Statistical analysis of the Ddx4 family included 68 orthologous proteins from 46 species, with remaining intrinsically disordered regions of proteins in the genomes being used as a reference state. The sliding charge score was calculated as the net charge in a 10-residue window, normalized by the probability of finding that charge window within the reference proteins. Similarly, the spacing of FG/GF/RG/GR motifs was calculated as the significance of occurrence in the Ddx4 orthologous set, normalized by the significance of occurrence in the background set (Supplemental Experimental Procedures Section 7). Having defined a position-specific “fingerprint” of the arrangement of these motifs from Ddx4^N1^, we used it to find similar proteins in human, yeast (*S. cerevisiae*), and *E. coli* (K12) genomes.

For DNA uptake measurements, double- and single-stranded DNA (see Supplemental Information) was coupled to Atto647N (Atto-Tec) through a dT-C6 linker. DIC and fluorescence images were obtained after preparing organelles as described for the T_P_ experiments, with a total protein concentration of 162.5 μM and nucleic acid concentration of 1 μm. The partition free energy was defined as

Equation 1 $$K_{partition}=\frac{{\left[DNA\right]}^{outside}}{{\left[DNA\right]}^{inside}}=\frac{{\bar{E}}_{+DNA}^{outside}-{\bar{E}}_{empty}^{outside}}{{\bar{E}}_{+DNA}^{inside}-{\bar{E}}_{empty}^{inside}},$$

where *E* is the average emission intensity in the specified phase after excitation at 635 nm (Supplemental Experimental Procedures Section 8).

## Supplemental Information

Document S1. Supplemental Experimental Procedures and Figures S1–S7

Movie S1. Nucleation and Growth of Ddx4^YFP^ Organelles, Related to Figure 1 Scale bar, 10 μm. Time in seconds (top left of screen). Organelles can be seen to spontaneously appear inside HeLa cell nucleus.

Movie S2. Cold Shock Inducing Rapid Formation of Ddx4^YFP^ Organelles, Related to Figure 2 Scale bar, 10 μm. Time in minutes (top left of screen).

Movie S3. Osmotic Shock Causing Rapid Dissolution and Condensation of Ddx4^YFP^ Organelles, Related to Figure 2 Scale bar, 10 μm. Time in minutes (top left of screen).

Movie S4. Osmotic Shock Causing Rapid Dissolution and Condensation of Ddx4^YFP^ Organelles, Related to Figure 2 Scale bar, 10 μm. Time in minutes (top left of screen).

Document S2. Article plus Supplemental Information
