# Supplementary material for: Phase Transition of a Disordered Nuage Protein Generates Environmentally Responsive Membraneless Organelles
Source: Mol Cell. 2015 Mar 5;57(5):936–47. doi: 10.1016/j.molcel.2015.01.013 (PMC4352761; doi:10.1016/j.molcel.2015.01.013)
Supplement: Document S1. Supplemental Experimental Procedures and Figures S1–S7 [file mmc1.pdf]

Molecular Cell, Volume *57*

Supplemental Information

Phase Transition of a Disordered Nuage Protein Generates Environmentally Responsive Membraneless Organelles

Timothy J. Nott, Evangelia Petsalaki, Patrick Farber, Dylan Jervis, Eden Fussner, Anne Plochowietz, Timothy D. Craggs, David P. Bazett-Jones, Tony Pawson, Julie D. Forman-Kay, and Andrew J. Baldwin

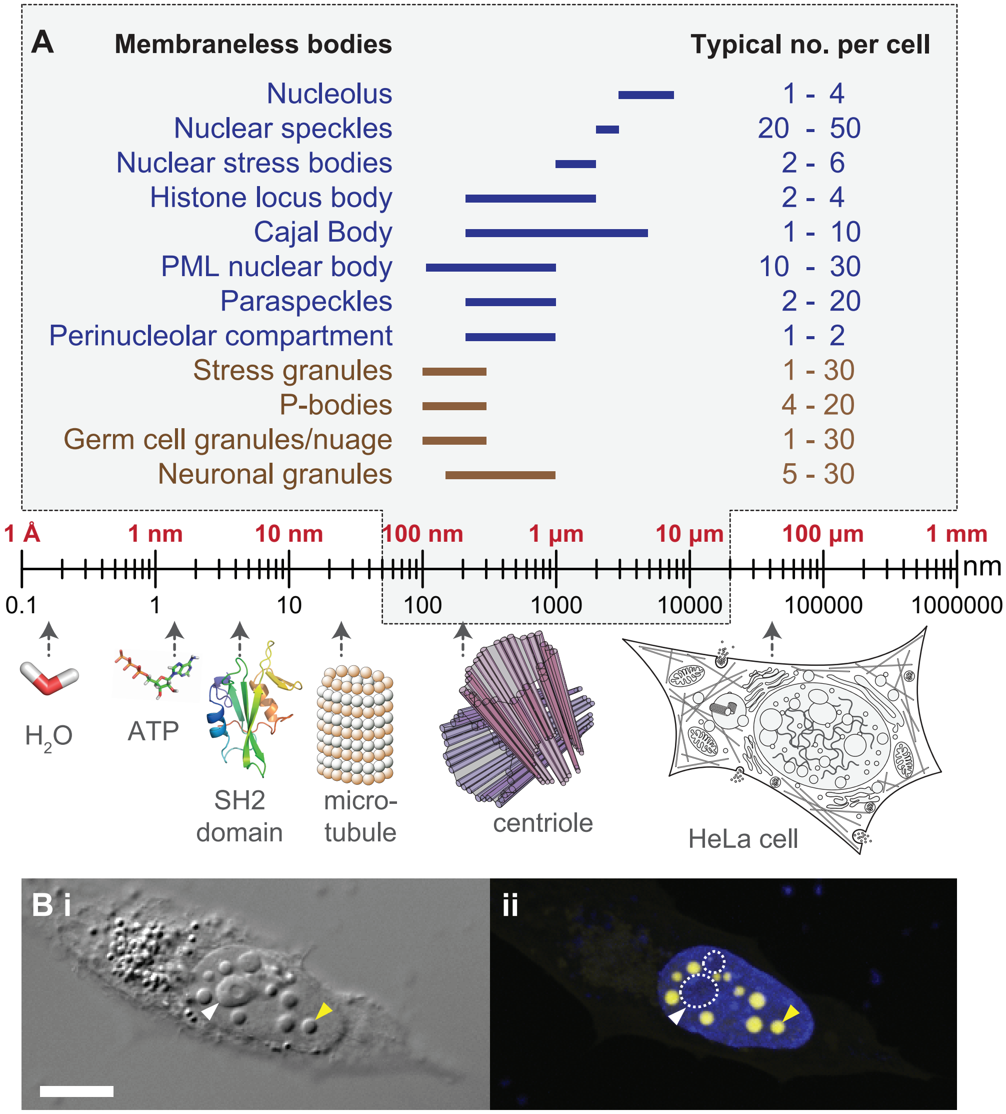

Figure S1 related to Figure 1: A Table and schematic showing the relation between the relative sizes and number of membraneless nuclear organelles commonly observed within cells compared to the sizes of other sub-cellular components. Table (gray) adapted from (Dundr and Misteli, 2010). **B.** Differential interference contrast (DIC, i) and corresponding extended focus fluorescence intensity (ii) images of a HeLa cell expressing Ddx4^{YFP}. Ddx4^{YFP} forms dense, spherical organelles (yellow arrow head) in the nucleus. The nucleolus is highlighted with white arrowheads and dashed white line (ii). Hoechst stain (blue) was used to stain chromatin within the nucleus. Scale bar 10 µm.

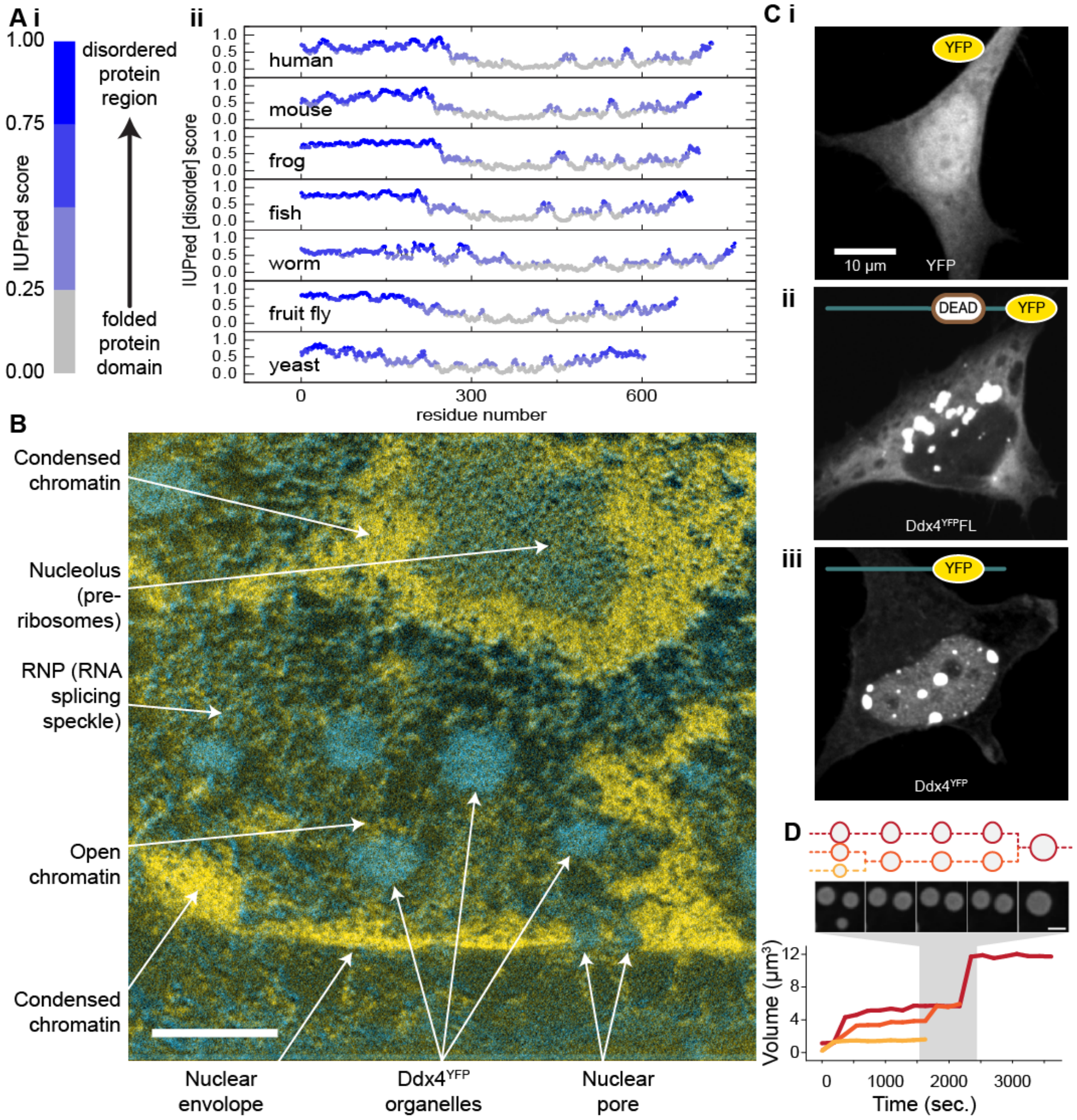

Figure S2 related to Figure 2 – Properties of isolated Ddx4 domains. A. IUPred predictions for Ddx4 orthologs. **B.** Phosphorus (yellow) and nitrogen (cyan) ESI images of HeLa cells containing Ddx4^{YFP} organelles. Scale bar 0.5 µm. **C.** Extended focus images of fixed HeLa cells expressing YFP alone (i), Ddx4^{YFP}FL (ii), and Ddx4^{YFP} (iii). **D.** Coalescence of Ddx4^{YFP} organelles within the nucleus of a HeLa cell. Scale bar 2 µm.

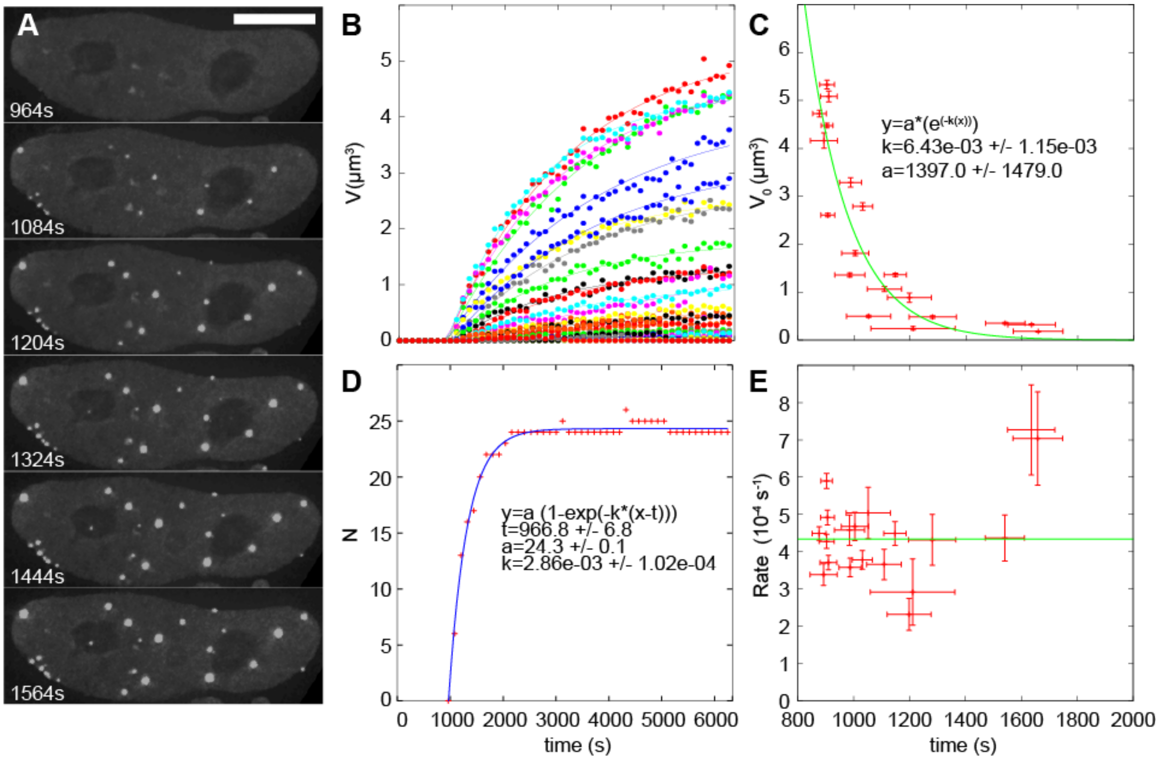

Figure S3 related to Figure 3: Kinetic analysis of growth of Ddx4^{YFP} organelles within the HeLa nucleus. A. Extended focus fluorescence image series of the initial growth of Ddx4^{YFP} organelles from a pool of dispersed material with a HeLa cell nucleus. Scale bar 10 μm. **B.** The volume of individual droplets of Ddx4^{YFP} within a single HeLa nucleus was followed with time. The growth curves were well described by an Avrami model of nucleated growth (Equation S6) where each droplet has an independent growth rate, *k*, steady-state volume, V_T and nucleation time *t'*. **C.** The variation in the steady-state volume of individual droplets varied with the time that they first appeared and decreased with time according to $\boldsymbol{V_T(t) = V_T^0 e^{-k_F t}}$ where k_F = 6.4 ± 1.2 x 10^{-3} s^{-1} . The extrapolated value for $V_T{}^0$ was 1397 μm^3. Error bars come from the standard error in the curve fitting (B). **D.** The number of unique droplets was found to increase such that $\boldsymbol{N(t) = N_0\left(1 - e^{-k_N(t-t_0)}\right)}$ where N_0 = 24.3 ± 0.1, k_N = 29 ± 1 x 10^{-4} s^{-1} and t_0 is the time for the appearance of the first droplet, 967 ± 7 s. **E.** The growth rates were found to cluster around a central value, though variation was observed between individual droplets such that the average and standard deviation rates were 4.5 and 1.2 x 10^{-4} s^{-1}. Error bars come from the standard error in the curve fitting (B).

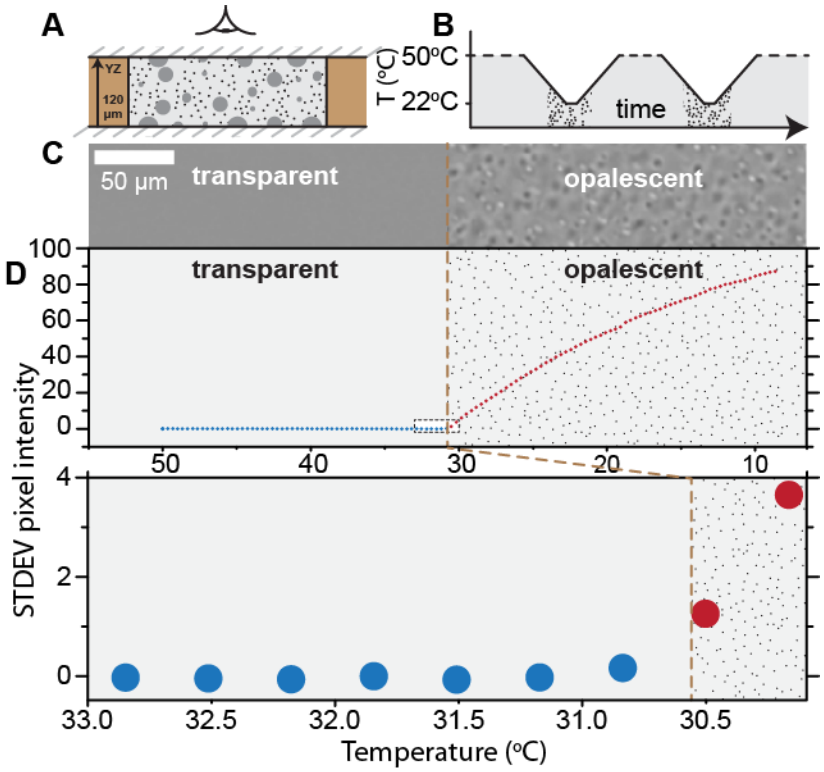

Figure S4 related to Figure 4– Estimation of the binodal phase transition temperature T_p. A. A schematic showing the arrangement of the sealed sample chamber on top of the THMS600 thermal stage with respected to the observer. Top and bottom coverslips (gray dashes) and SecureSeal imaging spacers (brown) were assembled to form a sealed sample chamber containing a protein solution **B.** Heating cycles showing that phase separation was induced by cooling, and reversed upon re-heating. **C.** Bright field images (XY-plane) of $Ddx4^{N1}$ above and below the transition temperature. **D.** Example of the determination of the $Ddx4^{N1}$ transition point by the change in standard deviation in pixel intensity with temperature (i) and close up showing that the transition was determined to be 30.5°C.

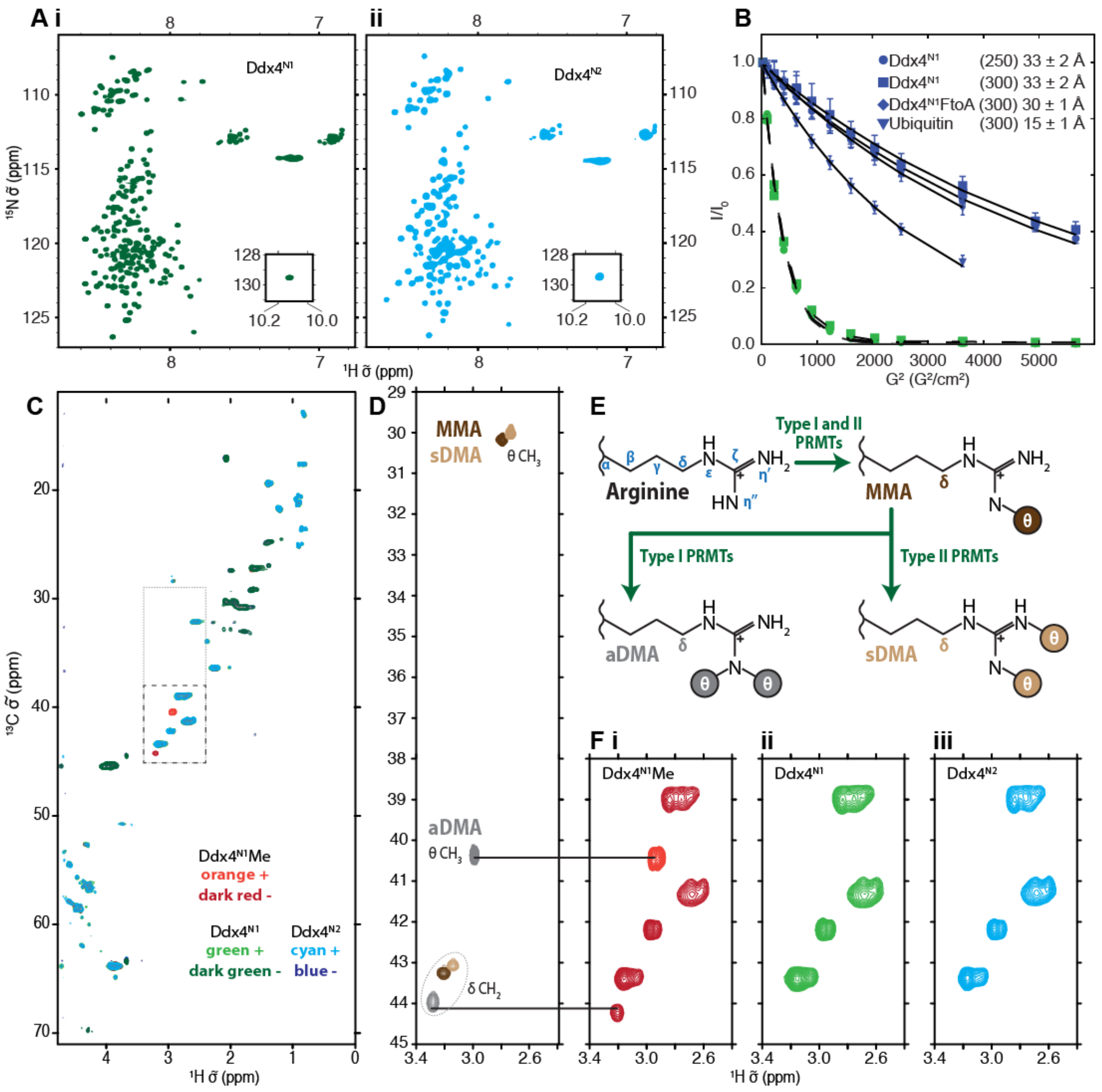

Figure S5 related to Figure 5 – NMR spectroscopy analysis of Ddx4^{N1}, Ddx4^{N2} and Ddx4^{N1}Me.

A. ^{15}N-^{1}H HSQC spectra of Ddx4^{N1} (i) and Ddx4^{N2} (ii). From the dispersion of chemical shifts both proteins are intrinsically disordered. **B.** The loss of intensity as a function of gradient field strength in a PFGSE experiments from various Ddx4 and Ubiquitin constructs with NaCl concentrations in mM in brackets and R_H values. The error bars indicate the standard deviation of the normalized intensity from individual peaks at each gradient strength. **C.** ^{13}C-^{1}H HSQC spectra of Ddx4^{N1} (greens), Ddx4^{N2} (blues) and Ddx4^{N1}Me (reds). Positive contours indicate CH_3/CH group and negative contours indicate CH_2 groups. The peak positions and intensities are identical, apart from the appearance of an additional two resonances in the box indicated upon methylation. **D.** NMR spectra of symmetric dimethylated arginine (sDMA, light brown), asymmetric dimethylated arginine (aDMA, grey) and mono-methylated arginine (MMA, dark brown). The three different chemical modifications are

clearly distinguished by NMR spectroscopy. **E.** The locations of the three different modifications. **F.** Comparison of the spectra of Ddx4^{N1}Me (red), Ddx4^{N1} (green) and Ddx4^{N2} (blue). The two additional resonances observed in the methylated form are consistent only with the aDMA form, and were assigned using recently published values for θ CH_3 and δ CH_2 groups from short peptides (Theillet et al., 2012). The observation that the resonances from the $^{\theta}CH_3$ groups of up to six aDMA residues are completely overlapped indicates that all such methyl groups experience relatively similar chemical environments, consistent with the disordered nature of the N-terminus of Ddx4.

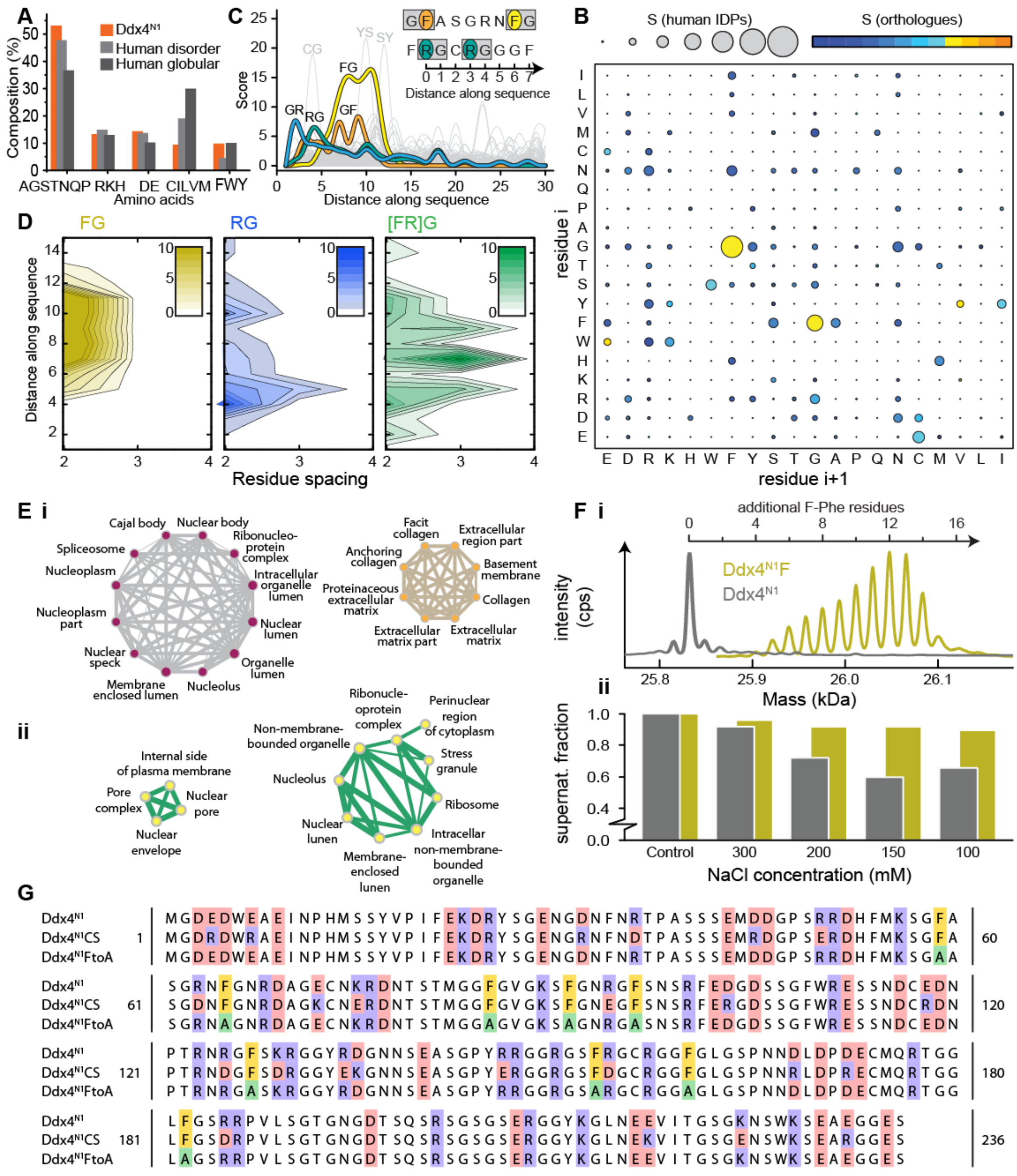

Figure S6 related to Figure 6: Statistical analysis of the sequence-based features that enable Ddx4^{N1} to form droplets.

A. Residue types in Ddx4^{N1} compared to globular and disordered portions of the human proteome. **B.** Frequency and statistical significance of pairs of amino acids in the disordered regions of human Ddx4 and its orthologs over a background signal from the proteomes of these species. 46 organisms were considered, from which 68 Ddx4 orthologs were identified. The size of the dots indicates the over-representation of a given pair in Ddx4 orthologs over those in the remaining intrinsically disordered parts of the proteomes of the 46 organisms. Colour represents the conservation of a given

amino acid pairing in the disordered regions of the Ddx4 ortholog family where warmer colours indicate a higher significance. The GF and FG pairing is both overrepresented when compared to the background proteome (larger size) and is highly conserved within the Ddx4 family (warmer colour). **C.** The separation between pairs of dipetides within Ddx4 disordered regions, normalised by their background frequency. Significant trends were observed for both FG/FG and the RG/GR spacings, suggesting the presence of strong evolutionary pressure to maintain these distances within the disordered regions of Ddx4 proteins. **D.** Similarity maps showing the spacings of F-F, R-R and F-R (each followed or preceded by a G) residues, extended to 3 and 4 repeats. **E.** The gene ontology (GO) terms for the top 10% of identified sequences were analysed and represented here as a network map from the human (i) and yeast (ii) genomes. The terms that occur are shown in text, where the size of the node indicates how likely the term was to appear in the dataset, over the background. The weight of the lines indicates the number of times that a sequence contains both GO terms. **F.** Fluorination of phenylalanine residues destabilises Ddx4 organelles. Incorporation of DL-3-flourophenylalanine (F-Phe) into $Ddx4^{N1}$ ($Ddx4^{N1}$F), measured by mass spectrometry (i), and its effect on phase-separation as monitored by remaining soluble protein following centrifugation (ii). **G.** Alignment of the amino acid sequences of $Ddx4^{N1}$, $Ddx4^{N1}$CS and $Ddx4^{N1}$FtoA. Residues involved in mutational strategies are highlighted.

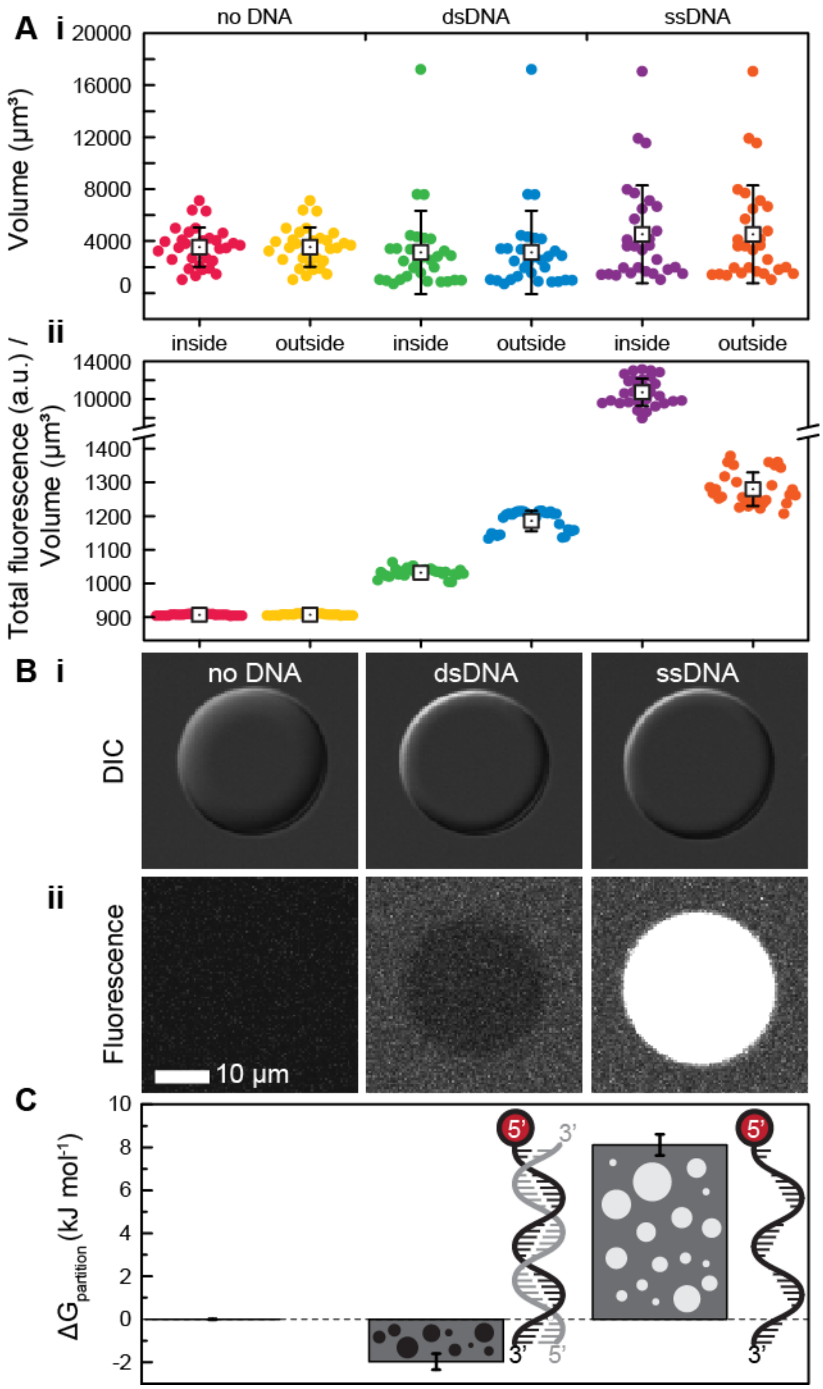

Figure S7 related to Figure 7: Ddx4^{N1} organelles both in isolation, and with double stranded and single stranded DNA of 32 bases in length. **A i).** Volumes of organelles and surrounding aqueous phase used in analysis. **ii)** Volumes normalised by total fluorescence to account for differences in ROI size. Error bars indicate one standard deviation. **B i)** DIC and **ii)** fluorescence images of a single representative organelle, acquired and processed identically. The fluorescence signal in the nucleic acid free sample was used as a baseline and subtracted from those containing ssDNA or dsDNA. **C.**

The partition free energy of the difference substances. The error bar of the empty organelle indicates the experimental uncertainty inherent to the experimental setup.

Supplementary movies:

Movie M1 related to Figure 1. Nucleation and growth of $Ddx4^{YFP}$ organelles. Scale bar 10 μm. Time in seconds (top left of screen). Organelles can be seen to spontaneously appear inside a HeLa cell nucleus.

Movie M2 related to Figure 2. Cold shock inducing rapid formation of $Ddx4^{YFP}$ organelles. Scale bar 10 μm. Time in minutes (top left of screen).

Movies M3 and M4 related to Figure 2. Osmotic shock causing rapid dissolution and condensation of $Ddx4^{YFP}$ organelles. Scale bar 10 μm. Time in minutes (top left of screen).

Supplementary Experimental Procedures

S1) Protein expression and purification

Genes for Ddx4 mutants ($Ddx4^{YFP}$, $Ddx4^{N1}$, $Ddx4^{N2}$, $Ddx4^{N1}$FtoA and $Ddx4^{N1}$CS) were synthesized by GenScript and sub-cloned. The recombinant Ddx4 proteins were expressed from IPTG-inducible plasmids (unless otherwise stated a modified pETM-30 vector containing the pGEX-2T-TEV site and pProEx multiple cloning site) in *E. coli* BL21(DE3) cells overnight at 20°C. Cell pellets were suspended in buffer (50 mM Tris pH 8.0, 500 mM NaCl, 5 mM DTT) and lysed by homogenization. Proteins were purified by affinity chromatography (GST-4b beads; GE Healthcare Life Sciences), the tag was removed with TEV protease, eluted and further purified and buffer-exchanged by size-exclusion chromatography into storage buffer (20 mM Tris pH 8.0, 300 mM NaCl, 5 mM TCEP). Purified proteins were centrifugally concentrated, typically to 300-500 μM, flash-frozen in liquid nitrogen and stored at -80°C.

Methylated Ddx4, $Ddx4^{N1}$Me, was produced by co-transforming competent *E. coli* BL21(DE3) cells with IPTG-inducible plasmids containing $Ddx4^{N1}$ (residues 1-236; kanamycin resistance marker) and PRMT1 (ampicillin-resistance marker). Colonies containing both plasmids were selected on agar plates containing both kanamycin and ampicillin before expression and purification as previously described. As recombinant PRMT1 did not contain a TEV-cleavable site it remained bound to GST beads when the methylated Ddx4 protein was eluted.

Isotopically enriched Ddx4 proteins were grown in M9 minimal media containing $^{15}NH_4Cl$ as the major nitrogen source for ^{15}N labeling and D-[^{1}H-^{13}C] glucose as the major carbon source for ^{13}C labeling. Nitrogen and carbon isotopes were purchased from Cambridge Isotope Labs. Incorporation of DL-3-flourophenylalanine Ddx4 ($Ddx4^{N1}$F) was produced by growing Ddx4 proteins in M9 minimal media supplemented with 0.1% $^{14}NH_4Cl$ and 0.3% D-[^{1}H-^{12}C] glucose as the sole sources of nitrogen and carbon. Labeling with 3-fluorophenylalanine was achieved by allowing cell cultures at 37°C to reach an OD_{600} of 0.8, whereupon 1 g/L glyphosate, 75 mg/L L-tryptophan, and 75 mg/L L-tyrosine was added. Once cell cultures reached an OD_{600} of 1.0 (after approximately 1 h), 150 mg/L DL-3-fluorophenylalanine was added and expression was induced with the addition of IPTG at 20°C. Purification was performed as described above. The mass of purified proteins was confirmed by electrospray ionization mass spectrometry.

For functional assays DNA coupled to the fluorescent dye Atto647N at the 5' end through a dT-C6 linker (marked as **X**) was synthesized. The sequence of this oligo (referred to as *oligo) was **X**TTTTTCCTAGAGAGTAGAGCCTGCTTCGTGG. Unlabelled sense and antisense versions of *oligo were also synthesized. Single and double stranded DNA (ssDNA and dsDNA, respectively) was prepared by

mixing *oligo with its sense or antisense strand at 1:1 molar ratio. The *oligo mixtures were then heated at 95°C for 3 min and cooled to 40°C over 45 minutes to allow labelled and unlabelled oligos to anneal.

HeLa Cell culture

HeLa cells were cultured on 35 mm glass-bottomed MatTek dishes or 25 mm glass coverslips in growth media (high glucose DMEM containing 20 mM HEPES pH 7.4, 10% FBS and antibiotics at 37°C and 5% CO_2). Ddx4 constructs ($Ddx4^{YFP}$, $Ddx4^{YFP}$FL, $Ddx4^{YFP}$FtoA and $Ddx4^{YFP}$CS) were expressed in HeLa cells from pcDNA 3.1+ (Invitrogen) plasmids by transient transfection utilizing the Effectene (Qiagen) or polyethylenimine (PEI) methods. Transfections were carried out according to the manufacturers instructions and used 0.5 – 1 μg plasmid DNA per MatTek dish or coverslip.

Live cell imaging

Live cell imaging experiments (Figure 2 A-C, S3 A-E and movies M1-M4) were performed on a Leica DMIRE2 inverted microscope equipped with a PZ-2000 XYZ series automated stage with Piezo Z-axis top plate (Applied Scientific Instrumentation) and Hamamatsu C10600-10B (ORCA-R2) camera. Cells under observation were live. Microscope hardware, image acquisition and analysis were controlled with Volocity software. Samples were observed with wide field illumination using a YFP filter set (excitation filter = 500 nm, emission filter = 535 nm) and a Leica HCX PL APO 40x oil immersion objective, numerical aperture (NA) 1.3.

Growth of $Ddx4^{YFP}$ organelles was sampled every 2 minutes for a total of 54 time points. At every time point 41 Z-slices (0.4 μm step size) were captured, each with an exposure time of 13 ms at bin 1, and with a bit depth of 12 (gray values from 0 - 4095). For the duration of the experiment the cell under observation remained in the center of the Z-stack. After acquisition, out of focus light was reduced using a point spread function (PSF), calculated for the optics of the system. The PSF had a Z-spacing of 0.05 μm, lateral spacing in X-Y of 0.067 μm, medium refractive index of 1.52, NA of 1.3 and emission wavelength of 535 nm. Automated corrections for photo-bleaching, sample movement and contrast enhancement were performed using Volocity software. $Ddx4^{YFP}$ organelles were identified as regions with >6 standard deviations in pixel intensity higher than the mean of the field of view (a rectangle encompassing the whole cell). This definition was used to determine the volume of $Ddx4^{YFP}$ organelles from deconvolved image stacks. Individual $Ddx4^{YFP}$ organelles were tracked from one frame to the next using the automated tracking function within Volocity.

Temperature and tonicity-responses of $Ddx4^{YFP}$ organelles were measured in HeLa cells *in situ* on the microscope. To vary the temperature of the cell between the second and third time point, growth media was rapidly aspirated, and replaced with 2 ml growth media, pre-cooled on ice. Image capture,

deconvolution, and image thresholding for volumetric measurements was the same as described above except the exposure time during acquisition was 15 ms per Z-slice.

The effect of tonicity on Ddx4^{YFP} organelles was assayed using the osmotic shock method. Tonicity was changed by aspirating growth media (and setting it aside at 37°C) before replacing it with 2 ml of ddH_2O that had been pre-warmed to 37°C. Once Ddx4^{YFP} organelles had dissolved, the ddH_2O was aspirated and replaced with the original growth media (at 37°C). Stacks of 76 Z-slices (0.4 μm step size, 3 ms exposure time) were acquired every minute at bin 2. Image capture, deconvolution and image thresholding for volumetric measurements was performed as described previously.

In vitro droplet preparation for microscopy

Small volumes of Ddx4^{N1} and Ddx4^{YFP} dense liquid phase were prepared *in vitro* using the vapor-diffusion hanging drop method. This entailed a droplet containing purified protein and buffer equilibrating by vapor diffusion with a larger reservoir containing the buffer alone. Hanging droplets were supported on siliconized coverslips (22 mm diameter, 0.22 mm thickness) from Hampton Research, sealed to the well with Vaseline. To initiate rapid phase separation, 1.5 μl of Ddx4^{N1} or Ddx4^{N1}Me was deposited on a coverslip, diluted 1:1 with 0 mM NaCl buffer (20 mM Tris pH 8.0, 5 mM TCEP), and equilibrated over a 150 mM NaCl reservoir solution. Once equilibrated (>30 min at 22°C), coverslips were taken off the well, and excess Vaseline removed with a 200 μl pipette tip. The droplet was then gently dispersed onto a microscope slide, and sealed in place with the remaining Vaseline.

S2) *In vitro* and live cell imaging

Confocal fluorescence microscopy

Confocal fluorescence microscopy was used to observe Ddx4^{YFP} in living and fixed HeLa cells and for measurement of the diffusion properties of purified Ddx4 protein *in vitro*. These experiments were performed using an Olympus IX81 inverted microscope equipped with 60x (NA 1.3) and 30x (NA 1.05) silicon immersion objectives. Microscope hardware and laser optics were controlled by FLUOVIEW FV1000 software.

Imaging fixed HeLa cells

HeLa cells expressing YFP, Ddx4^{YFP}, Ddx4^{YFP}FL, Ddx4^{YFP}FtoA and Ddx4^{YFP}CS were grown on MatTek dishes or 25 mm glass coverslips, and fixed with 4% paraformaldehyde (PFA) in phosphate buffered saline (PBS), for 5 minutes at 37°C. Cells were then washed three times with PBS to remove excess PFA. Next, cells were permeabilised with 0.5% TritonX-100 (in PBS) for 10 minutes, and again washed three times with PBS. Nuclei were visualized with Hoechst or DAPI stain. Cells were washed a further two times with PBS to remove excess Hoechst/DAPI stain and imaged using an Olympus

IX81 inverted microscope with a 60x (NA 1.3) silicon immersion objective. Hoechst/DAPI dye was excited with a 405 nm laser and YFP was excited with a 515 nm laser. Hoechst/DAPI and YFP fluorescence were detected at 461 and 527 nm respectively. Differential interference contrast (DIC) images were collected using illumination from the 405 nm laser.

For immunofluorescence experiments, HeLa cells expressing $Ddx4^{YFP}$ were grown on 25 mm diameter #1.5 glass coverslips (Warner Instruments). Fixation and permeabilisation of samples was performed as above. Cells were then blocked with goat serum (5% in PBS) for one hour at room temperature before antibody staining. Primary antibodies were diluted to between 1:10 and 1:100 (in PBS containing 5% goat serum) before use. Nucleoli and PML bodies were simultaneously labelled using mouse B23 (Santa Cruz sc-56622) and rabbit PML (Abcam ab-72137) antibodies, respectively. Nuclear speckles and Cajal bodies were simultaneously labelled using mouse SC-35 hybridoma (ATCC 1023768), and rabbit coilin (Santa Cruz sc-32860) antibodies, respectively. Following incubation at $4^{o}C$ overnight, excess primary antibodies were removed by washing the cells three times with PBS (five minutes per wash).

Cells were then incubated with Cy3 goat anti-mouse, and Cy5 goat anti-rabbit, secondary antibodies (diluted 1:400 and 1:200 in PBS, respectively) for 1 hour at room temperature. Excess secondary antibodies were removed by washing with PBS, as above. Nuclei were visualized with DAPI stain. Coverslips were then mounted on microscope slides using glycerol/*n*-propyl gallate mounting medium, sealed with nail varnish, and imaged using an Olympus IX81 inverted microscope equipped with a 60x (NA 1.3) silicon immersion objective. DAPI, YFP, Cy3 and Cy5 dyes were excited with 405 nm, 515 nm, 559 nm and 635 nm lasers, respectively. 26 Z-slices (0.4 μm spacing, 12.5 μs $pixel^{-1}$ scanning speed, 12 bit depth) were captured for each channel.

Fluorescence-recovery after photo-bleaching (FRAP)

FRAP experiments on $Ddx4^{YFP}$ organelles in live HeLa cells were performed at $37^{o}C$ and 5% CO_2 in a live cell chamber (Precision Plastics Ltd) mounted on an Olympus IX81 inverted microscope with a 60x (NA 1.3) silicon immersion objective. Photo-bleaching of a 1.5 μm $Ddx4^{YFP}$ organelle was achieved using a 515 nm laser with a bleaching time of 1 second. Fluorescence emission was monitored at 527 nm. Images (150 x 112 pixels, 12 bit depth) were captured at 218 ms intervals with a scanning speed of 2 μs $pixel^{-1}$ for 100 time points.

In vitro FRAP experiments were performed on samples of the dense phase of Ddx4, prepared using the hanging drop method. $hDdx4^{N1}$, free CFP and $hDdx4^{YFP}$ (all in storage buffer) were first mixed to produce a sample containing the three proteins at individual concentrations of 292.5, 8.1 and 8.1 μM respectively. This mixture was then diluted 1:1 with 0 mM NaCl buffer (20 mM Tris pH 8 at RT; 5 mM

TCEP) and equilibrated as a hanging drop over a well containing 150mM NaCl buffer for 48 hours. 30 minutes prior to the FRAP experiment the cover slip supporting the hanging droplet was removed from the well, excess Vaseline removed with a yellow (200 µL) pipette tip, and the cover slip placed on a microscope slide such that the hanging droplet was dispersed across its surface and sealed in place with the remaining Vaseline. *In vitro* FRAP was performed on a 10 µm diameter droplet of dense-phase protein using a 515 nm laser (YFP channel) and a 440 nm laser (CFP channel) to simultaneously bleach both fluorophores. Laser power (transmissivity) for the bleach period was 100% for 0.2 seconds and 1% for acquisition. Fluorescence emission was monitored at 527 nm (YFP channel) and 476 nm (CFP channel). Images (512 x 512 pixels, 12 bit depth) were captured at both emission wavelengths with a scanning speed of 2 µs pixel^{-1} at 3 second intervals for a total of 360 time points. The data were analysed using to Fick's law of Diffusion. In one dimension, the number of particles at a distance x from the source at time t will be given by

$$n(x,t) = n_0 Erfc\left(\frac{x}{2\sqrt{Dt}}\right) \quad [S1]$$

where Erfc is the complimentary error function. If we bleach a droplet with a known beam size, the time required for a particle at the center of the droplet to reach half that of the outside, then the diffusion coefficient *D* is given by:

$$\frac{1}{t_{1/2}}\left(\frac{x}{2Erfc^{-1}1/2}\right)^2 = D \quad [S2]$$

where Erfc^{-1}(0.5)~0.4769 and x is the radius of the beam. Recovery of the fluorescence of free protein, outside of organelles both in cells and *in vitro* was too fast for our measurements. We estimated a decay rate based on 99% recovery within the measurement time. The diffusion measurements we obtained in this way were comparable to the diffusion rates of freely diffusing protein measured using NMR (vide infra, figure S5). Diffusion of protein in organelle droplets both *in vivo* and *in vitro* were two orders of magnitude lower than the value obtained both for freely diffusing protein.

Electron Spectroscopic Imaging (ESI) microscopy

Samples were fixed and processed for EM as previously described (Ahmed et al., 2008). Briefly, Ddx4^{YFP} structures were identified using correlative LM/ESI by indirect immunofluorescence labeling of YFP after paraformaldehyde fixation and triton permeabilization. Samples were post-fixed with glutaraldehyde, dehydrated in an ethanol series, and embedded in Quetol 651 resin before ultra-thin sectioning with a Leica microtome. Samples were placed on copper finder grids to identify structures of interest and carbon coated with a 3-5 nm carbon film to improve sample stability. Nitrogen ratio maps were collected at 200 kV on a Tecnai transmission electron microscope of Ddx4

nuclear foci at energy offsets of 383 and 416 eV using a GATAN energy filter. To analyze the relationship between the Ddx4^{YFP} nuclear foci and neighbouring structures, such as chromatin, we collected phosphorus ratio maps at energy offsets of 120 and 155 eV. Images were processed with ImageJ and Photoshop to demonstrate the relationship between chromatin, nuclear bodies, and Ddx4^{YFP} structures. Protein-based structures are pseudo-coloured blue, and chromatin structure are pseudo-coloured in yellow. Nitrogen levels were normalised to zero in chromatin-rich regions, so that only the protein-dense domains that are not chromatin-associated are visualized. Data analyses were preformed on non-normalised, unfiltered images.

S3) NMR Spectroscopy

All experiments were performed on Varian INOVA spectrometers (11.7 and 18.8 T) equipped with room-temperature triple resonance probes. NMR spectra were processed using the NMRpipe suite of programs (Delaglio et al., 1995) and visualized in Sparky (Goddard and Kneller, 2004). ^{15}N-^{1}H sensitivity enhanced HSQC spectra of 150 μM Ddx4^{N1} were recorded at 20°C at a field strength of 18.8 T in 20 mM sodium phosphate, 400 mM NaCl, 5mM DTT, and 10% D_20 at pH 6.0. A typical experiment employed acquisition times of (40, 64) ms (t1, t2) with a pre-scan delay of 1s, 100 increments and 16 scans per increment giving a total experiment time of 1 hour. ^{13}C-^{1}H constant time HSQC spectra of 200 uM ^{13}C enriched Ddx4^{N1}, Ddx4^{N2} and Ddx4^{N1}Me were recorded at 25°C at a field strength of 18.8 T in 20mM Tris, 300 mM NaCl, 0.5 mM TCEP and 10% D_2O at pH 8. A typical experiment employed acquisition times of (30, 64) ms (t1, t2) with a pre-scan delay of 1.5s, 218 increments with 8 scans per increment giving a total experiment time of 1 hour 34 minutes. ^{13}C-^{1}H natural abundance constant time HSQC spectra of 10mM methyl arginine standards, aDMA, sDMA and MMA were recorded in 10% D_2O at 25°C. Typically, acquisition times of (30, 64) ms (t1, t2) with a pre-scan delay of 1s, with 128 increments and 8 scans per transient giving a total experiment time of 38 minutes.

Pulsed field gradient (PFG) diffusion experiments were performed at 37°C at 11.7 T on 130 μM samples of Ddx4^{N1} and Ddx4^{N1}FtoA in 10mM Tris, 250mM NaCl, 5mM TCEP, 0.04% dioxane and 10% D_20. A 1D ^{1}H pulse gradient stimulated echo longitudinal encode-decode (PG-SLED) experiments with a watergate solvent suppression was employed (Wilkins et al., 1999), with 512 transients averaged per 1D, and 12 linearly spaced gradient strengths with a total experimental time of 6 hours. Experiments were carried out with dioxane used as an internal standard with an effective hydrodynamic radius ($R_{h,ref}$) of 2.12 Å. Decay profiles were analyzed using an in-house written MATLAB program, where the signal I is expected to decay according to $I = Ae^{-dG^2}$ where G is the gradient strength and d and A are constants. The hydrodynamic radius of the protein $R_{h,prot}$ can be obtained from:

$$R_{h,prot} = \frac{d_{ref}}{d_{prot}}\left(R_{h,ref}\right)$$

[S3]

Where d_{ref} and d_{prot} are the decay rates for dioxane and the protein, respectively. Each chemical shift was individually interrogated and analysed to give a value for d_{prot} providing four or more gradient points had signal that was more than twice the height of the maximum noise. The decay rates were then averaged, and the standard deviation used to provide an uncertainty estimate.

S4) Mass Spectrometry

Electrospray ionization mass spectrometry (ESI-MS)

Electrospray ionization mass spectrometry (ESI-MS) analyses were conducted using a QStar XL quadrupole time-of-flight mass spectrometer (AB Sciex, Concord, ON) equipped with an Ionspray source and a modified HSID interface (Ionics, Bolton, ON). Online desalting of protein samples was accomplished using a size exclusion medium (Sephadex, GE Healthcare Biosciences, Piscataway USA) at a flow rate of 250 µL min^{-1}. The mobile phase was composed of one-to-one mixture of methanol/aqueous 0.1% formic acid. Protein mass spectra were processed using the Bayesian Protein Reconstruction algorithm implemented in the BioAnalyst 1.1.5 software package (AB Sciex, Concord, ON). The AIMS Mass Spectrometry Laboratory in the Department of Chemistry at the University of Toronto performed ESI-MS.

Fragmentation mapping of Ddx4^{N1}Me methylation sites

For reduction and alkylation, 40 µL of 10 mM DTT in 100 mM NH_4HCO_3 was added to the Ddx4^{N1}Me sample solution and incubated for 1 hour at 56°C before being cooled to room temperature. 20 µL of 55 mM iodoacetamine in 100 mM NH_4HCO_3 was subsequently added before incubated at room temperature for 45 minutes in the dark. Trypsin digestion was performed in 2 mL of 50 mM NH_4HCO_3 with 25 µg sequencing grade trypsin (Roche Diagnostic Cat# 11418475001) at a final concentration of 12.5 ng µL^{-1}. Digestion buffer was then added to the sample solution to reach protein to enzyme ratio of 100:1 and incubated overnight at 37°C. For digestion with endoproteinase GluC, protein samples were lyophilized after reduction and alkylation and reconstituted in GluC reaction buffer (50mM Tris-HCl, 0.5 mM Glu-Glu). GluC (New England Biolabs Cat# P8100S) was reconstituted by the addition of 50 µL Millipore water and added to the sample solution at a ratio of 20:1 enzyme:protein and incubated overnight at 25°C.

Digested sample solutions were lyophilized and reconstituted in 0.1% formic acid for LCMS/MS analysis. The digested peptides were loaded onto a 150 µm ID pre-column (Magic C18, Michrom Biosciences) at 4 µl min^{-1} and separated over a 75 µm ID analytical column packed into an emitter tip containing the same packing material. The peptides were eluted over 60 min. at 300 nl min^{-1} using a

0 to 40% acetonitrile gradient in 0.1% formic acid using an EASY n-LC nano-chromatography pump (Proxeon Biosystems, Odense Denmark). The peptides were eluted into an LTQ-Orbitrap hybrid mass spectrometer (Thermo-Fisher, Bremen, Germany) operated in a data dependent mode. MS was acquired at 60,000 FWHM resolution in the FTMS and MS/MS was carried out in the linear ion trap. 6 MS/MS scans were obtained per MS cycle. The raw data were searched using Mascot 2.3.02 (Matrix Sciences, London UK). The search result was analyzed using Scaffold 3.4.3 (Proteome Software Inc, Portland, US).

S5) Kinetic analysis and interpretation of growth of Ddx4^{YFP} organelles in the HeLa nucleus

Ddx4^{YFP} was transfected into HeLa cells 24 hours prior to live cell imaging, A brightly fluorescing cell, containing only diffuse Ddx4^{YFP}, was followed over time as described above. After a period of approximately 1000 s, droplets were observed to form within the nucleus and grow over time (see Supplementary Video M1, Supplementary Figure S3). A relatively small number of droplets were observed to appear in any given instant, rather than many appearing simultaensouly, suggesting that the growth mechanism was one of nucleated growth rather than spinodal decomposition or secondary nucleation.

In order to test this, we followed the volumes of individual droplets within a single HeLa cell nucleus as a function of time. As described in the text, the total volume occupied by droplets was followed as a function of time (Figure 1D). The resulting values were well approximated by the Johnson-Mehl-Avrami-Komogorov (JMAK) equation (Avrami, 1939; Johnson and Mehl, 1939; Kolmogorov, 1937) (Figure 1D), commonly used to describe nucleated phenomena:

$$V(t) = V_T\left(1 - e^{-gt^a}\right) \qquad \text{[S4]}$$

where a is termed the Avrami exponent, g is a characteristic rate and V_T is the total droplet volume that the system will tend towards at long times. The data were found to be well described by this theory, yielding an Avrami exponent of a = 1.01 ± 0.01, g = 3.7 ± 0. 1 x 10^{-4} s^{-1} and V_T = 42.8 ± 0.1 μm^3 (Figure 1D). In the case of spherical droplets growing, the Avrami coefficient would be expected to be on the order 4. The lower value observed here is consistent with a model where the quantity of free material is limiting.

To test this, we sought to derive expressions to describe the growth of the ensemble of droplets. Following the approach of Avrami, we note that the total volume of the system V=V_F+V_T, the sum of droplet volume V_T and free volume V_F. In terms of concentrations, the total moles of Ddx4 is given by $N = C_0V$ where C_0 is the initial concentration of free protein. In addition, $N_T = C_TV_T$, where C_T is the concentration of protein inside a drop, a constant related to the droplet density $\rho = M_WC_T$ and M_W is

the molar mass of the constituent molecule. Finally, the concentration of free protein will be $N_F = C_F V_F$. Through conservation of mass, the total number of moles will be $C_0 V = C_F V_F + C_T V_T$, making the approximation that the system is closed. As droplets form, the concentration of free monomers will fall, as will the volume of available space, as the droplet volume increases. The concentration of proteins in the droplets, the total volume available to a droplet and the initial available concentration will be constants. Following the lead of Avrami, the increase in the number of moles in the droplets will be equal to the product of the increase in infinite dilution multiplied by the number of free moles $dN_T = dN_C(N - N_T)$. Dividing by N, we can express the quantities in terms of mole fractions, x such that $dx = dx_C(1 - x)$.

If we make the assumption that the maximum rate of formation of droplet is linear in time such that $dx_C = kdt$, and if we integrate noting that $x(0)=0$ then we obtain the result in an exponential form $x(t) = (1 - e^{-kt})$. Expanding the mole fraction, we arrive at our final result for the growth of an individual droplet:

$$V_D = \frac{C_0}{C_D} V_T \left(1 - e^{-kt}\right) = \frac{C_0 M_W}{\rho} V_T = V_D^* \left(1 - e^{-kt}\right) \qquad [S5]$$

Thus each drop will be expected to expand until either the concentration of source material is depleted or it coalesces with another droplet from an adjacent volume element, leading to a steady-state volume of V_D^* for the droplet in question. In the experiments in the cell, the concentration of droplets was relatively low and few coalescence events were observed. Nucleation events are random, and so if droplet appearance and growth occurs at a nucleation time t' relative to the observed time t, then the final expression for growth of each droplet becomes:

$$V(t,t') = V_0(t')\left(1 - e^{-k(t-t')}\right) \qquad [S6]$$

where k is the characteristic growth rate and $V_0(t')$ is the steady-state volume. This equation was found to well describe the data (Supplementary Figure S3B) with all droplets having a similar growth rates (Supplementary Figure S3E).

Three constants, the total available volume, the initial free protein concentration and the protein concentration inside the droplets specify the steady-state volume. Empirically, the steady-state volume V_D^* was found to decrease exponentially with time (Supplementary Figure S3C), suggesting that the free protein concentration varies between successively nucleated droplets such that $C_F = C_0 e^{-k_F t}$, consistent with the falling concentration of free $Ddx4^{YFP}$ that would be expected to accompany droplet growth.

The number of individual droplets was found to increase hyperbolically with time. Such a situation would be expected for a nucleated process where the limiting step is the unimolecular conversion of

a monomer to an unfavourable conformation. If nucleus formation is reversible, and that the concentration of available nucleation sites is proportional to the excess concentration C_{ex} such that nucleus formation can only happen above a threshold, then

$$\frac{dC_{ex}}{dt} = -k_N^+ C_{ex} + k_N^- N \quad [S7]$$

The excess concentration is given by $C_{ex}(t) = (C_{ex}^0 - C_{ex}^\infty)e^{-k_N t} + C_{ex}^\infty$, where $k_N = k_N^+ + k_N^-$ and so

$$N = N_0(1 - e^{-k_N t}) \quad [S8]$$

where $N_0 = k_N^+/(k_N^+ + k_N^-)$. This equation is found to be in excellent empirical accord with the data (Supplementary Figure S3D). The nucleation rate is given by the time derivative:

$$\frac{dN}{dt} = N_0 k_N e^{-k_N t} \quad [S9]$$

Taken together, we can derive an expression for the total volume of the droplets that can be compared to the Avrami equation. The total volume occupied by droplets will be given by

$$V_T = \sum N_i V_i = \int_0^t \frac{dN}{dt} V_D^*(t')\left(1 - e^{-k(t-t')}\right)dt' \quad [S10]$$

where in the second step we replace the sum with an integral over the history of all the particles to the current time. Substituting in the expression for V_D^* and dN/dt and integrating yields

$$V_T = \frac{C_0 M_W N_0 k_N V}{\rho(k_N + k_F - k)(k_N + k_F)}\left(k\left(1 - e^{-(K_N + k_F)t}\right) + (k_N + k_F)\left(1 - e^{-kt}\right)\right) \quad [S11]$$

We note that as the values of the rates are comparable, and so this equation is essentially indistinguishable from the JMAK equation S4 with the fitting parameters obtained from the data.

Overall, the droplets appear to be in competition with each other for source material, as both the steady-state volume (Supplementary Figure S3C) and nucleation rates (Supplementary Figure S3D) decrease with time. The characteristic growth rate was found to be largely independent of time, as expected for nucleated growth (Supplementary Figure S3E). We conclude that the formation of $Ddx4^{YFP}$ organelles follow kinetics expected of a primary nucleation mechanism and that the expansion of the droplets is limited by the quantity of free $Ddx4^{YFP}$. In the cell, it is likely that the total concentration will be time dependent, reflecting factors such as translation rates.

S6) *In vitro* measurements of T_P

Experimental arrangement

Recombinant $Ddx4^{N1}$ and $Ddx4^{N1}Me$ binodal phase transition points were measured using a THMS600 thermal stage controlled by a CI94 control unit and LinkSys32 software. The THMS600 unit was mounted on an Olympus BX61 upright microscope equipped with a Hamamatsu 1394 ORCA-ERA camera. Microscope hardware and image acquisition protocols were controlled using Volocity software. Samples were observed in bright field with a 10x (NA 0.4) air immersion objective.

The THMS600 thermal stage was cooled using nitrogen gas flowing through a 10ft copper (refrigerator) coil immersed in a box of dry ice coupled to and from the nitrogen supply by thick Tygon tubing. Sample chambers were constructed using two 18 mm diameter coverslips (#1; 0.16-0.19 mm thick) sandwiching a SecureSeal imaging spacer (Sigma, 9 mm internal diameter) containing 9 µL protein sample (Supplementary Figure S4A). Sealed sample chambers were positioned in the center of the THMS600 silver heating block and initially held at 50°C for 1 minute followed by a linear cooling ramp of 2°C min^{-1} to 10°C. For samples where the phase transition temperature occurred above 40°C, the initial holding temperature was 65°C for 1 minute. For transitions below 18°C the final temperature was set to 5°C.

Stacks of 25 Z-slices (10 µm step size, 4 ms exposure time per slice) aligned in Z with respect to the middle of the sample chamber were captured every 10 seconds using Olympus Focus Drive hardware. Images were acquired at bin2 (672 x 512 pixels) with a 12 bit camera chip sensitivity (0-4095 range in pixel intensity). Image stacks were processed and analyzed with Volocity software. Initial image stacks were 672 x 512 x 25 pixels (i.e. ± 120 µm in Z from the center of the sample chamber). Stacks were cropped to 120 µm thick (13 Z-slices) to correspond to the approximate thickness of the imaging spacer (and hence internal height of the sample chamber). A region of 191 x 183 x 13 pixels free of dust/dirt was then selected for further analysis. The mean and standard deviation in pixel intensity was calculated for each 191 x 183 x 13 pixel data set.

Determination of the Phase Transition (T_p) boundary

A linear baseline of the standard deviation of pixel intensity was taken from the pre-transition hold temperature (typically 50°C). The phase transition temperature for each run was taken as the point where the pixel intensity deviated from the linear baseline by more than three times the standard error of the regression estimate at a 95% confidence level (Supplementary Figure S4D):

$$S_{yo}^2 = S_y^2 \left\{ 1 + \frac{1}{N} + \frac{(x_0 - \overline{x})^2}{S_{xx}} \right\} \quad [S12]$$

In cases where the baseline displayed curvature, a smaller region in the XY plane (71 x 60 x 13 pixels) was selected for analysis. Selecting a smaller area was found not to lower the accuracy of determination of T_p.

Analysis and interpretation of T_P phase diagram

The free energy change on two separate species mixing, according to Flory-Huggins theory is given by:

$$\Delta G_{mix} = G_{mixed} - G_{unmixed} = RT\left(n_1 \ln\phi_1 + n_2 \ln\phi_2 + n_1\phi_2 \frac{\chi}{R} \right) \quad \text{[S13]}$$

Where R is the gas constant, T is the thermodynamic temperature, Φ are the volume fractions of species 1 and 2 and χ, the Flory-Huggins interaction constant reflects additional interactions between the two species. The logarithmic terms evaluate the change in translational entropy of the two species from a lattice model. The volume fraction is given by:

$$\phi_1 = \frac{n_1 N_1}{n_1 N_1 + n_2 N_2} \quad \text{[S14]}$$

Where n_i is the number of molecules and N_i is a measure of the size of species *i* and $\Phi_1+\Phi_2=1$. We can express the volume fraction in terms of the protein and free water concentrations as $\phi_1 = N_1[Ddx4]/[H_2O]$, where $1/[H_2O] = V_1 N_A$ where N_A is Avogadro's constant and V_1 is the volume of a single water molecule. In what follows, water is species 2 and $N_2=1$, and Ddx4 is species 1, and so $N_1=236$, the number of residues in $Ddx4^{N1}$.

For polymers characterized by an upper critical solution temperature (UCST), at very low temperatures, the condensed phase is favoured at all solution compositions. As the temperature is increased, a spinodal point is reached where the condensed phase is no longer the most stable. Above this temperature, the condensed phase co-exists with a mixed state. As temperature is raised further, a binodal point is reached, above which the dispersed state is the most stable. Just below this temperature, condensed droplets are expected to appear in solution, leading to this temperature originally termed the 'cloud point'. This point is determined experimentally in our work, and a suitable expression will be derived. On mixing, two phases are created. When a small amount of material 1, *dv*, is allowed to pass from phase B to phase A, the free energy of both are affected such that:

$$dG_{rxn} = \left(\frac{dG_{mix}^{A}}{dn_1} \right)_{n_2} dv - \left(\frac{dG_{mix}^{B}}{dn_1} \right)_{n_2} dv \quad \text{[S15]}$$

The condition for equilibrium is that $dG_{rxn}/dv = 0$. Noting that the chemical potential of component *i* is $\Delta\mu_i = \left(d\Delta G/dn_i\right)_j$, it follows that the equilibrium condition is given by the twin conditions $\Delta\mu_1^A = \Delta\mu_1^B$, and $\Delta\mu_2^A = \Delta\mu_2^B$. The chemical potential of species 1 and 2 are obtained from equations S13 and S14:

$$\frac{\Delta\mu_1}{RT} = \ln\phi_1 + \phi_2\left(1 - \frac{N_1}{N_2}\right) + \frac{\chi}{R} N_1 \phi_2^2 \quad \text{[S16]}$$

$$\frac{\Delta\mu_2}{RT} = \ln\phi_2 + \phi_1\left(1 - \frac{N_2}{N_1}\right) + \frac{\chi}{R}N_2\phi_1^2$$ [S17]

These can both be expressed in terms of a single parameter *Φ*, the volume fraction of species 1. The equilibrium condition for a given value of chi is characterized by the mole fractions of species 1 in the two phases, Φ_A and Φ_B. In the case of N_1=N_2, a closed form expression can be derived. When this is not the case the equations can be solved numerically. In the case of dilute polymer solutions, the Flory-Huggins interaction parameter χ will be expected to be independent of Φ and contain enthalpic and entropic terms:

$$\chi = \frac{\Delta G}{T} = \frac{\Delta H}{T} - \Delta S$$ [S18]

Where *ΔH* and *ΔS* are the residual temperature independent entropy and enthalpy associated with the changing interactions that accompany the phase transition, and *T* is the thermodynamic temperature. Numerically solving equation equations S16 and S17 subject to the equilibrium conditions for a given value of *ΔH* and *ΔS* enables T_P, the temperature for equilibrium to be determined.

The calculated curves for T_P varying with Ddx4 concentration were found to be in excellent agreement with the experimental (Figure 4A). Numerical fitting of the equation enables the interaction parameters, *ΔS* and *ΔH* to be determined (Figure 4B and C). It is interesting to note that the agreement between the model and the data, and the scaling of *ΔH* and *ΔS* (Figure 4B and C) is not a function of the constants $[H_2O]$ and N_1. The specific values obtained for *ΔH* and *ΔS* however do have some dependence on the choice of these two values.

Variance of entropy and enthalpy interaction parameters with salt concentration

Both enthalpy and entropy parameters were found to vary significantly with salt concentration (Figure 4B and C). In the condensed phase, we would expect a combination of both ionic and non-ionic interactions between chains. Increasing the salt concentration would be expected to screen the ionic component according to the following potential for two single charge groups:

$$U = \frac{e^2 N_a}{4\pi\epsilon_0\epsilon_r r} e^{-r/r_d}$$ [S19]

where *e* is the charge on the electron, N_a is Avogadro's constant, ε_0 is the permittivity of free space, ε_r is the relative permittivity, or dielectric of the medium, *r* is the equilibrium spacing, r_d is the Debye or interaction length given by $r_d = \sqrt{\epsilon_0\epsilon_r RT/\left(F^2 I\right)}$, and *I* is the concentration dependent part of ionic strength, $I = [NaCl]$.

Taken together, the enthalpy would be expected to scale as $\Delta H = \Delta H_0 + U$. This model gives an excellent description of the enthalpy parameter (Figure 4B), revealing a relative permittivity within the condense phase of 45 ± 13, an interaction spacing of 13 ± 2 Å, and a charge independent contribution ΔH_0 that is close to zero, -0.058 ± 0.137 kJ mol^{-1}. At low salt (50mM) the contribution to the enthalpy is predominantly ionic (U = 1 kJ mol^{-1}). At 300 mM salt, this interaction is almost entirely screened, and there is no longer an enthalpic preference for the chains to remain within droplets.

The entropic contribution to the interaction parameter was also observed to have a relatively weak dependence on salt, with a relatively modest 7% difference between the highest and lowest values (Figure 4C). Empirically, the scaling can be described by:

$$\Delta S = \Delta S_0 + B_0 R \log[NaCl] \qquad [S20]$$

Where the residual entropy ΔS_0 was determined to be -6.9 ± 0.1 J mol^{-1} K^{-1} and the coefficient B_0 was -0.128 ± 0.007 J mol^{-1} K^{-1}. The form of this scaling is interesting. At higher salt concentrations, the excess entropy is more negative, indicating that the condensed phase is favoured. The hydrodynamic radius of free Ddx4 was not observed to vary with increasing salt concentration, suggesting that in addition to having weaker interactions between chains, they also become more mobile.

Phase transition propensity

The effect of ionic strength on the phase transition propensity of Ddx4^{N1}F in comparison to Ddx4^{N1} was investigated. Samples of 400 μM Ddx4^{N1}F and Ddx4^{N1} were prepared in buffers containing 50mM Tris pH 8.0, 300mM NaCl, and 2mM DTT. In order to induce phase separation, 20 μL samples were then rapidly diluted into buffers with 300, 200, 150, and 100mM NaCl with a final protein concentration of 140 μM and allowed to equilibrate for 24 h. The samples were then centrifuged at 16,000 rpm for 10 min, and 2 μL of the supernatant was taken for SDS-PAGE gel electrophoresis. Band intensities were quantified using Image Lab software (Bio-Rad) and normalised to a control sample containing 400 μM protein, 50mM Tris pH 8.0, 300mM NaCl and 2mM DTT that was rapidly diluted to 140 μM protein, 50mM Tris pH 8.0, 300mM NaCl and 2mM DTT and a sample immediately taken for SDS-PAGE gel electrophoresis.

S7) Statistical analysis of the sequence features that give rise to organelle formation

Datasets

As a training set we used all proteins in metazoans that were orthologous to Ddx4. Specifically we extracted from eggNOG (Powell et al., 2012) the orthologous group meNOG12075 (DEAD (Asp-Glu-

Ala-Asp) box polypeptide 4) comprising 68 proteins from 46 species. As a background set we used all the sequences in the proteomes of these 46 species. When applying our strategy to discover new proteins with features similar to Ddx4 and to assess whether these include proteins with the potential to form biologically relevant non-membrane-bound organelles we use the human proteome extracted from UniprotKB (The UniProt Consortium, 2012) and redundancy reduced at 40% identity level using cd-hit (Li and Godzik, 2006). We used IUPred (Dosztanyi et al., 2005) (cutoff of 0.3, setting long, in order to exclude disordered regions shorter than 20-30 residues long, and structural domain regions, while still including as many of the disordered regions as possible) to extract the disordered regions of the sequences in these proteins. All the following analyses were performed on the disordered regions of these datasets.

Sliding window method for calculating net charge index

In order to investigate patterns of charged residues within protein sequences, we define the "net charge" index, I, within a 10 residue window to be $I = RK - ED$ where RK and ED is the ratio of the counts of the specified residues within a 10 residue window to that in all IDP regions in the human proteome (Figure 6A).

Calculation of residue pair preferences

The frequency of all observed pairs in the disordered regions of our positive dataset (meNOG12075) is calculated as the 'observed' frequency, and our background set as the 'expected' frequency. We then normalise these frequencies, using the probability of observing these residue pairs by random chance, based on the set's sequence amino acid composition. Finally we calculate the significance of the observed values, as the ratio of the observed normalised frequencies compared to the expected ones. This is represented in Supplementary Figure S6B, where the size of the dots represents the normalised frequency of amino acid pairs in the disordered regions of the Ddx4 human protein and the colour represents the significance of observation of the particular pair in the Ddx4 orthologous set.

Similarity 'fingerprint' generation

The inter-residue spacing of all possible dipeptides was calculated. We scanned each sequence and upon an occurrence of our residue of interest e.g. R flanked by G, we calculated the frequency of observing the next such residues in each distance from 1 to 30. The frequency was then normalised by the probability of observing this pair by chance. The resulting matrices were divided by similar matrices derived from the background set. These represent the profiles of blocks containing our residue pairs of interest in the Ddx4 orthologs and how these differ compared to the background set (Supplementary Figure 6C).

Sequence scanning

We used the matrices generated from the [FR]G blocks, the RG and the FG blocks with the hypothesis that these features of Ddx4 will lead to the identification to more similar proteins that are also able to form phase separations or gel-like structures within the cell. For each sequence we used a sliding window of length 30 and scored them based on the matrices generated previously, keeping only the hits with score >0. We performed this on the human proteome, after we reduced its redundancy by 40% using cd-hit in order to avoid bias when studying their functional enrichment (Li and Godzik, 2006). We then look at the hits with at least one hit with score greater than 2 to see the over-represented GO terms. We further scanned in a similar fashion (after extracting only the disordered regions) the Baker's yeast (*Saccharomyces cerevisiae*) proteome and the *E. coli* proteome (strain K12).

Functional enrichment analysis

Functional enrichment analyses were performed using DAVID and applying and EASE score cutoff of 0.05 to the results. (Da Wei Huang and Lempicki, 2008; Sherman and Lempicki, 2009). The results were represented using the 'Enrichment Map' plugin in Cytoscape and a p-value cutoff of 0.05, an overlap coefficient of 0.6 and a FDR Q-value of 0.1 (Shannon et al., 2003).

S8) DNA uptake into organelles (nucleic acid partitioning)

Organelles were prepared as for the hanging drop experiments by mixing 1.5 µl Ddx4^{N1} protein (325 µM in 300 mM NaCl buffer) with 1.5 µl of a 0 mM NaCl buffer containing either no DNA or 2 µM total ssDNA or dsDNA, such that the salt dilution resulted in immediate organelle formation and a final concentration of 1 µM total fluorophore per sample. DIC and laser confocal images were obtained using an Olympus IX81 inverted microscope equipped with a 30x (NA 1.05) silicon immersion objective. Fluorescence excitation was achieved with a 635nm laser. Fluorescence emission between 650 – 750 nm was collected using the same objective. Stacks of 23 Z-slices (each 1024x1024 pixels, acquired with 4 µs pixel^{-1} scanning speed, 1 µm step size, 1% laser power) centered in Z with respect to the middle of the sample were captured for 3 fields of view containing Ddx4^{N1} droplets. Microscope hardware and image acquisition was controlled with FLUOVIEW software. Image analysis was performed with Volocity software. Inspection of Z-stacks revealed that the samples were 9 µm thick and were therefore initially cropped in Z to 9 slices before further analysis. Next, 60 regions of interest (ROIs; 10 inside Ddx4^{N1} droplets and 10 outside Ddx4^{N1} droplets per field of view) were selected and the total emission, *E*, was computed and normalised by ROI volume (Supplementary Figure S7A). Residual average fluorescence from the sample containing no DNA was then subtracted from the ssDNA and dsDNA-containing samples and the average and standard deviation (overbar) in total fluorescence calculated. The partition coefficient was defined as:

$$K_{partition} = \frac{[DNA]^{outside}}{[DNA]^{inside}} = \frac{\overline{E}^{outside}_{+DNA} - \overline{E}^{outside}_{empty}}{\overline{E}^{inside}_{+DNA} - \overline{E}^{inside}_{empty}} \qquad [S21]$$

The partition coefficient was converted into a free energy via $\Delta G_{partition} = -RTlnK_{partition}$.

Supplementary references

Ahmed, K., Li, R., and Bazett-Jones, D.P. (2008). Electron spectroscopic imaging of the nuclear landscape. In The Nucleus (Springer), pp. 415-423.

Avrami, M. (1939). Kinetics of phase change. I. General theory. J. Chem. Phys. *7*, 1103.

Da Wei Huang, B.T.S., and Lempicki, R.A. (2008). Systematic and integrative analysis of large gene lists using DAVID bioinformatics resources. Nat. Protoc. *4*, 44-57.

Delaglio, F., Grzesiek, S., Vuister, G.W., Zhu, G., Pfeifer, J., and Bax, A. (1995). NMRPipe: a multidimensional spectral processing system based on UNIX pipes. J. Biomol. NMR *6*, 277-293.

Dosztanyi, Z., Csizmok, V., Tompa, P., and Simon, I. (2005). The pairwise energy content estimated from amino acid composition discriminates between folded and intrinsically unstructured proteins. J. Mol. Biol. *347*, 827-839.

Dundr, M., and Misteli, T. (2010). Biogenesis of nuclear bodies. Cold Spring Harb Perspect Biol *2*, a000711.

Goddard, T., and Kneller, D. (2004). SPARKY 3. University of California, San Francisco.

Johnson, W.A., and Mehl, R.F. (1939). Reaction kinetics in processes of nucleation and growth. Trans. Aime *135*, 396-415.

Kolmogorov, A.N. (1937). On the statistical theory of the crystallization of metals. Bull. Acad. Sci. USSR, Math. Ser *1*, 355-359.

Li, W., and Godzik, A. (2006). Cd-hit: a fast program for clustering and comparing large sets of protein or nucleotide sequences. Bioinformatics *22*, 1658-1659.

Powell, S., Szklarczyk, D., Trachana, K., Roth, A., Kuhn, M., Muller, J., Arnold, R., Rattei, T., Letunic, I., and Doerks, T. (2012). eggNOG v3. 0: orthologous groups covering 1133 organisms at 41 different taxonomic ranges. Nucleic Acids Res. *40*, D284-D289.

Shannon, P., Markiel, A., Ozier, O., Baliga, N.S., Wang, J.T., Ramage, D., Amin, N., Schwikowski, B., and Ideker, T. (2003). Cytoscape: a software environment for integrated models of biomolecular interaction networks. Genome Res. *13*, 2498-2504.

Sherman, B.T., and Lempicki, R.A. (2009). Bioinformatics enrichment tools: paths toward the comprehensive functional analysis of large gene lists. Nucleic Acids Res. *37*, 1-13.

The UniProt Consortium (2012). Reorganizing the protein space at the Universal Protein Resource (UniProt). Nucleic Acids Res. *40*, D71-75.

Theillet, F.X., Smet-Nocca, C., Liokatis, S., Thongwichian, R., Kosten, J., Yoon, M.K., Kriwacki, R.W., Landrieu, I., Lippens, G., and Selenko, P. (2012). Cell signaling, post-translational protein modifications and NMR spectroscopy. J Biomol NMR *54*, 217-236.

Wilkins, D.K., Grimshaw, S.B., Receveur, V., Dobson, C.M., Jones, J.A., and Smith, L.J. (1999). Hydrodynamic radii of native and denatured proteins measured by pulse field gradient NMR techniques. Biochemistry *38*, 16424-16431.
