# Supplementary figures and images for: Phase Transition of a Disordered Nuage Protein Generates Environmentally Responsive Membraneless Organelles
Source: Mol Cell. 2015 Mar 5;57(5):936–47. doi: 10.1016/j.molcel.2015.01.013 (PMC4352761; doi:10.1016/j.molcel.2015.01.013)

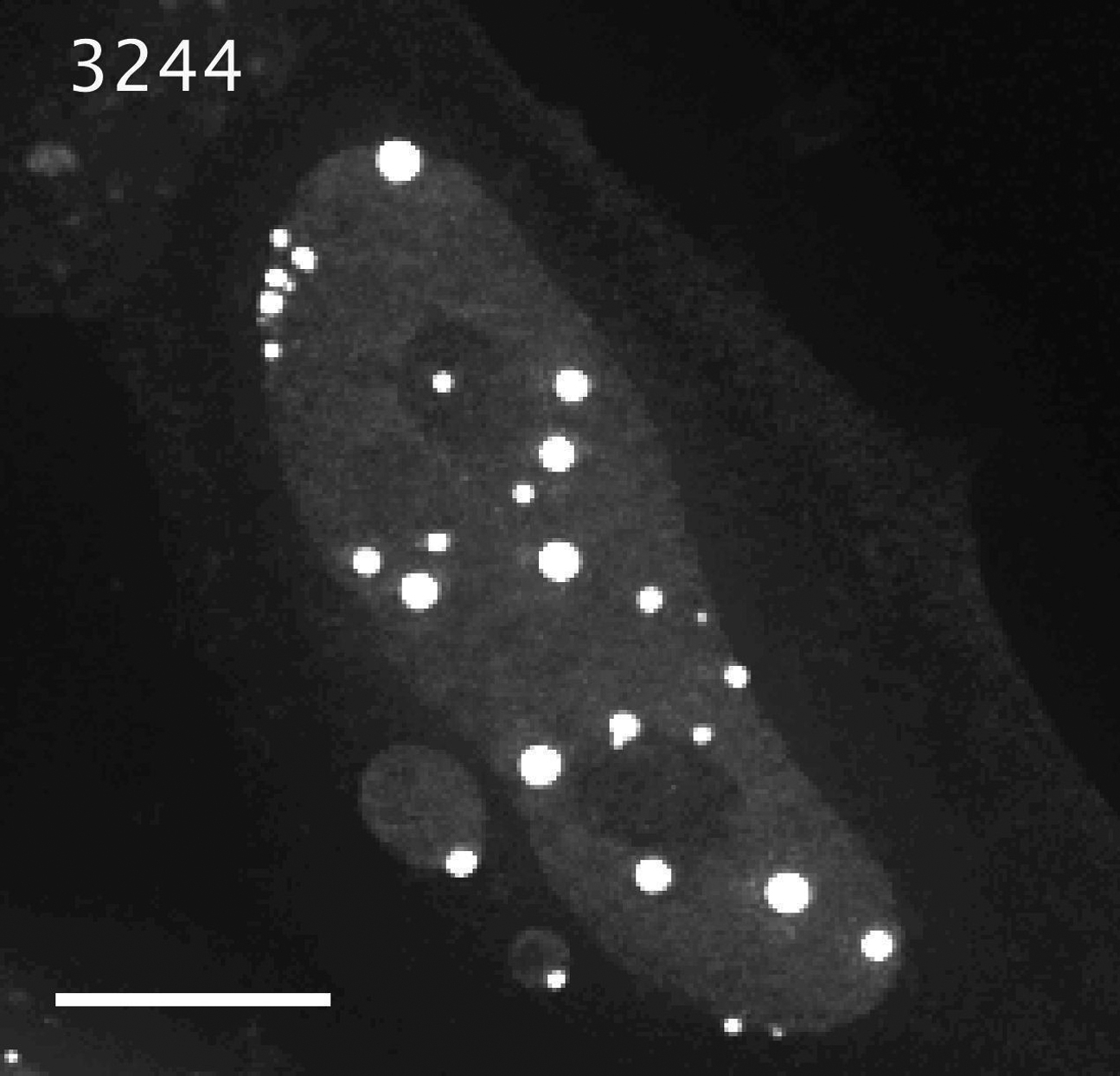

Supplement: Movie S1. Nucleation and Growth of Ddx4YFP Organelles, Related to Figure 1 — Scale bar, 10 μm. Time in seconds (top left of screen). Organelles can be seen to spontaneously appear inside HeLa cell nucleus. [file mmc2.jpg]

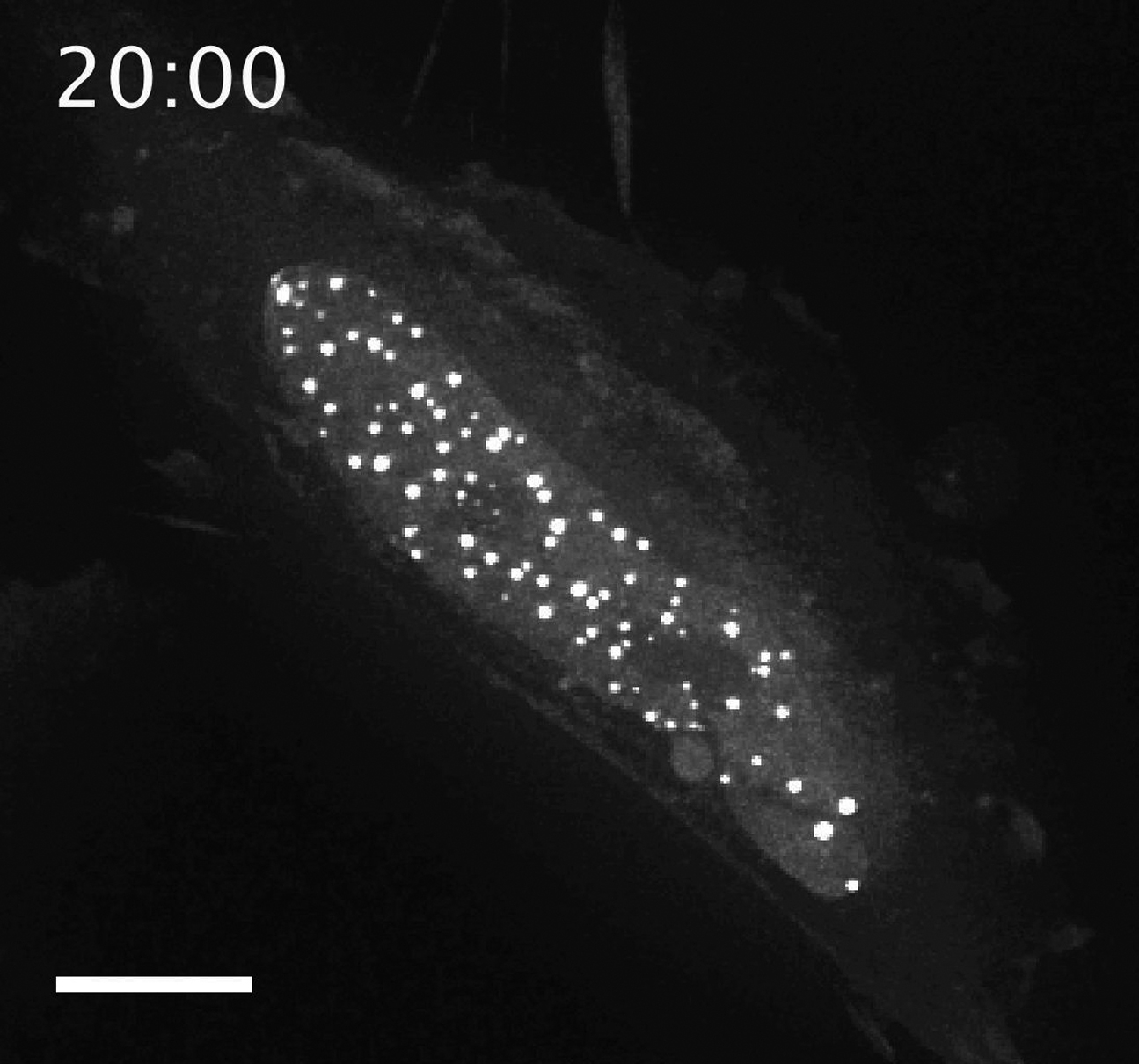

Supplement: Movie S2. Cold Shock Inducing Rapid Formation of Ddx4YFP Organelles, Related to Figure 2 — Scale bar, 10 μm. Time in minutes (top left of screen). [file mmc3.jpg]

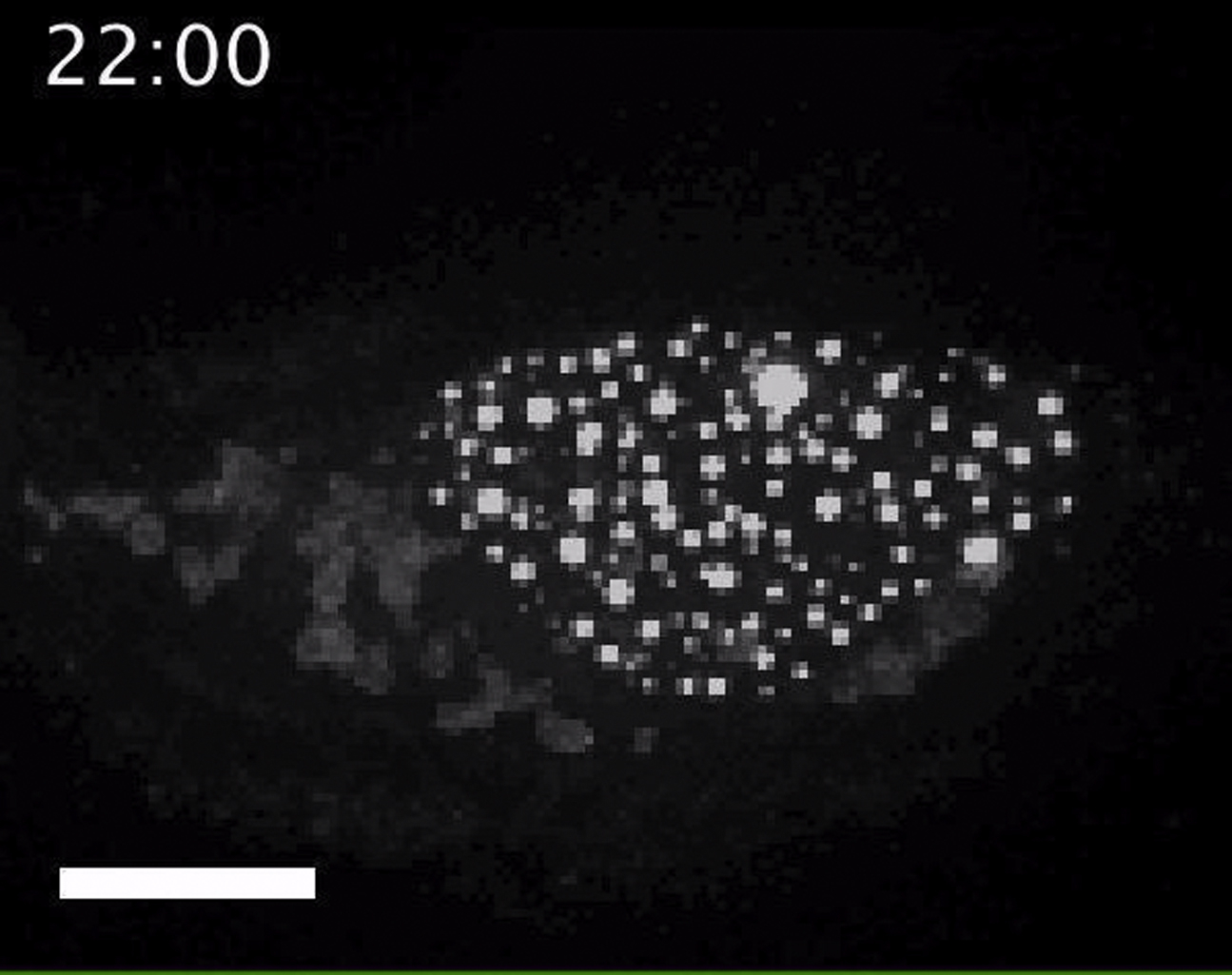

Supplement: Movie S3. Osmotic Shock Causing Rapid Dissolution and Condensation of Ddx4YFP Organelles, Related to Figure 2 — Scale bar, 10 μm. Time in minutes (top left of screen). [file mmc4.jpg]

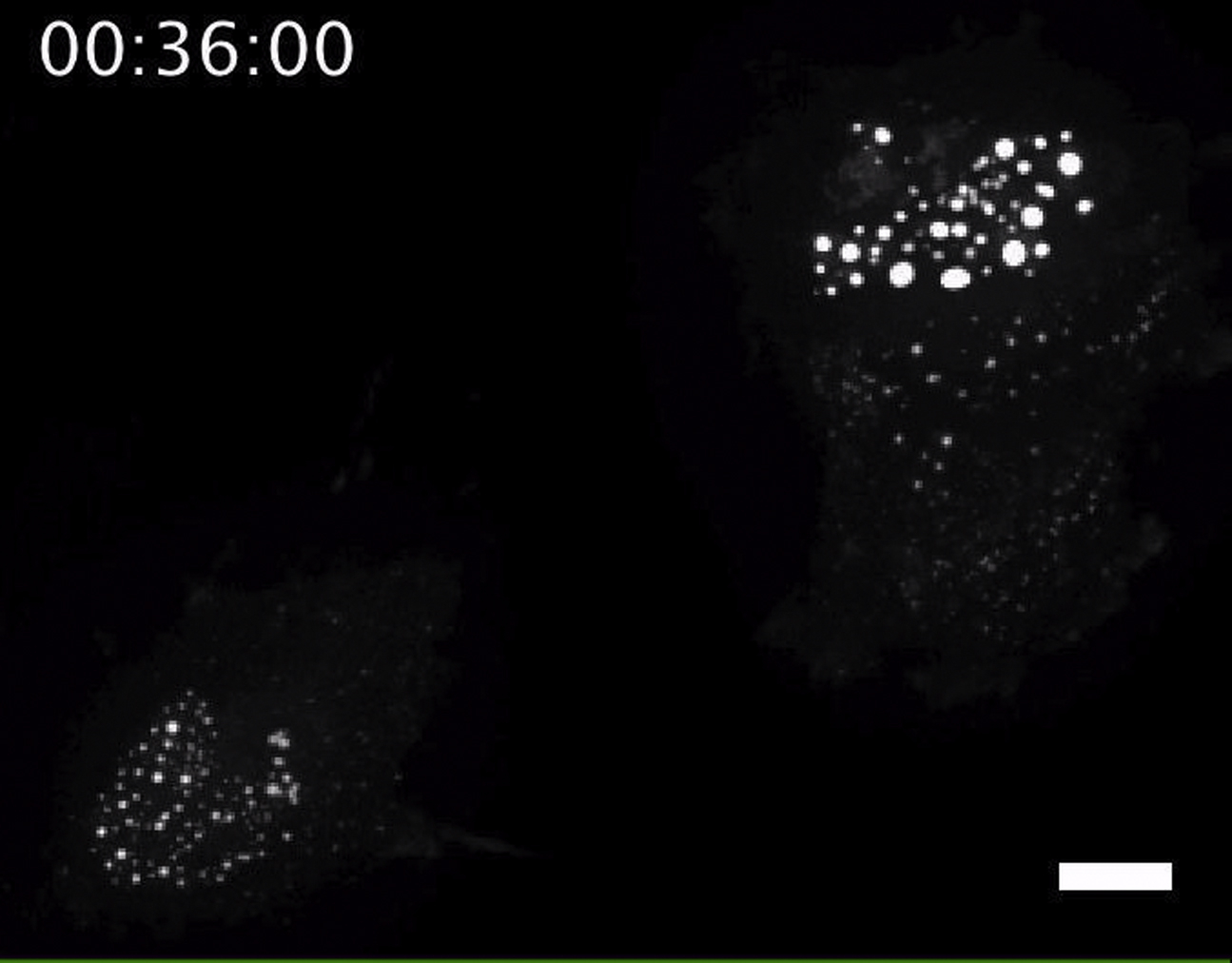

Supplement: Movie S4. Osmotic Shock Causing Rapid Dissolution and Condensation of Ddx4YFP Organelles, Related to Figure 2 — Scale bar, 10 μm. Time in minutes (top left of screen). [file mmc5.jpg]
