# Supplementary material for: Phase Transition of a Disordered Nuage Protein Generates Environmentally Responsive Membraneless Organelles
Source: Mol Cell. 2015 Mar 5;57(5):936–47. doi: 10.1016/j.molcel.2015.01.013 (PMC4352761; doi:10.1016/j.molcel.2015.01.013)
Supplement: Document S2. Article plus Supplemental Information [file mmc6.pdf]

Molecular Cell

Article

Phase Transition of a Disordered Nuage Protein Generates Environmentally Responsive Membraneless Organelles

Graphical Abstract

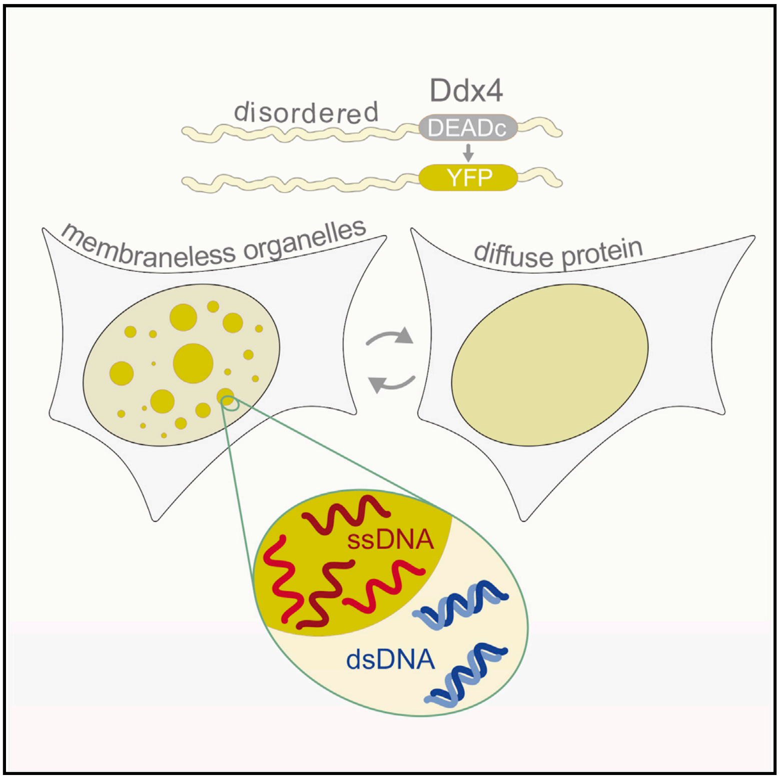

Highlights

- Intrinsically disordered N terminus of Ddx4 forms organelles in cells and in vitro
- Phase transition to form organelles is driven by electrostatic interactions
- Methylation, ionic strength, and temperature changes can dissolve the organelles
- Sequence determinants of formation are common in membraneless organelle proteins

Authors

Timothy J. Nott, Evangelia Petsalaki, ..., Julie D. Forman-Kay, Andrew J. Baldwin

Correspondence

forman@sickkids.ca (J.D.F.-K.),
andrew.baldwin@chem.ox.ac.uk (A.J.B.)

In Brief

Nott et al. demonstrate that a single protein constituent can reversibly form membraneless organelles both in vitro and in cells. The bodies provide an alternative solvent environment that concentrates single-stranded, but exclude double-stranded DNA. We propose that phase separation of disordered proteins is a general mechanism for forming regulated, membraneless organelles.

Nott et al., 2015, Molecular Cell *57*, 936–947
March 5, 2015 ©2015 The Authors
http://dx.doi.org/10.1016/j.molcel.2015.01.013

CellPress

Molecular Cell
Article

Phase Transition of a Disordered Nuage Protein Generates Environmentally Responsive Membraneless Organelles

Timothy J. Nott,[1,2] Evangelia Petsalaki,[1] Patrick Farber,[3] Dylan Jervis,[4] Eden Fussner,[1] Anne Plochowietz,[5] Timothy D. Craggs,[5] David P. Bazett-Jones,[3,6] Tony Pawson,[1,7] Julie D. Forman-Kay,[3,6,*] and Andrew J. Baldwin[2,*]
[1]Lunenfeld-Tanenbaum Research Institute, Mount Sinai Hospital, Toronto, ON M5G 1X5, Canada
[2]Physical and Theoretical Chemistry Laboratory, University of Oxford, Oxford OX1 3QZ, UK
[3]Research Institute, Hospital for Sick Children, 686 Bay Street, Toronto, ON M5G 0A4, Canada
[4]Department of Physics, University of Toronto, 60 St. George Street, Toronto, ON M5S 1A7, Canada
[5]Clarendon Laboratory, University of Oxford, Oxford OX1 3PU, UK
[6]Department of Biochemistry, University of Toronto, 1 King's College Circle, Toronto, ON M5S 1A8, Canada
[7]Recently deceased
*Correspondence: forman@sickkids.ca (J.D.F.-K.), andrew.baldwin@chem.ox.ac.uk (A.J.B.)
http://dx.doi.org/10.1016/j.molcel.2015.01.013
This is an open access article under the CC BY license (http://creativecommons.org/licenses/by/4.0/).

SUMMARY

Cells chemically isolate molecules in compartments to both facilitate and regulate their interactions. In addition to membrane-encapsulated compartments, cells can form proteinaceous and membraneless organelles, including nucleoli, Cajal and PML bodies, and stress granules. The principles that determine when and why these structures form have remained elusive. Here, we demonstrate that the disordered tails of Ddx4, a primary constituent of nuage or germ granules, form phase-separated organelles both in live cells and in vitro. These bodies are stabilized by patterned electrostatic interactions that are highly sensitive to temperature, ionic strength, arginine methylation, and splicing. Sequence determinants are used to identify proteins found in both membraneless organelles and cell adhesion. Moreover, the bodies provide an alternative solvent environment that can concentrate single-stranded DNA but largely exclude double-stranded DNA. We propose that phase separation of disordered proteins containing weakly interacting blocks is a general mechanism for forming regulated, membraneless organelles.

INTRODUCTION

Biochemical reactions in the cell frequently have mutually exclusive solution requirements, leading to a need to keep them spatially separated. Membrane encapsulation is a commonly used strategy in roles ranging from controlling the flow of genetic information, via the nucleus and the ER, to maintaining the isolated acidic environment within a lysosome. An alternative strategy involves the formation of membraneless, proteinaceous organelles, including the prominent nucleolus (Montgomery, 1898), PML bodies (de Thé et al., 1990; Melnick and Licht, 1999), Cajal bodies (Cajal, 1903) and nuclear speckles (Cajal, 1910) in the nucleus, and P bodies and both stress and germ granules in the cytoplasm. These cellular structures have been described as coacervates (Hyman and Brangwynne, 2011; Wilson, 1899) and are optically resolvable as spherical micron-sized droplets. The absence of a surrounding membrane enables these organelles to rapidly assemble or dissolve following changes in the cell's environment and in response to intracellular signals, critical for cellular integrity and homeostasis (Dundr and Misteli, 2010) (Figure S1).

A striking feature of membraneless organelles is that their largely proteinaceous interior partially excludes the bulk aqueous phase (Brangwynne, 2011; Hyman and Brangwynne, 2011). Such organelles behave as liquid droplets. Fluorescence recovery after photobleaching (FRAP) experiments interrogating organelles such as the nucleolus and Cajal bodies indicate that their constituent molecules internally diffuse rapidly (Phair and Misteli, 2000), and P-granules, the worm analog of mammalian nuage or germ granules, condense from a pool of diffuse constituents following specific biological cues (Brangwynne et al., 2009). Moreover, spherical nucleoli of the amphibian oocyte have been observed to coalesce when in close contact and show a size distribution that obeys a simple power law, indicating the formation of liquid droplets (Brangwynne et al., 2011). On a residue level, sequences of low complexity, such as repeated RG, QN, and YG repeats, are important for forming RNA granules, stress granules and P bodies (Decker et al., 2007; Kato et al., 2012; Sun et al., 2011). An understanding of the interactions that stabilize such structures and regulate their biogenesis, as well as a rationale for their biochemical function, has remained elusive.

To address these questions, we have studied a dominant protein constituent of a membraneless organelle as a model. Ddx4 proteins are essential for the assembly and maintenance of the related nuage in mammals, P-granules in worms, and pole plasm and polar granules in flies (Liang et al., 1994).

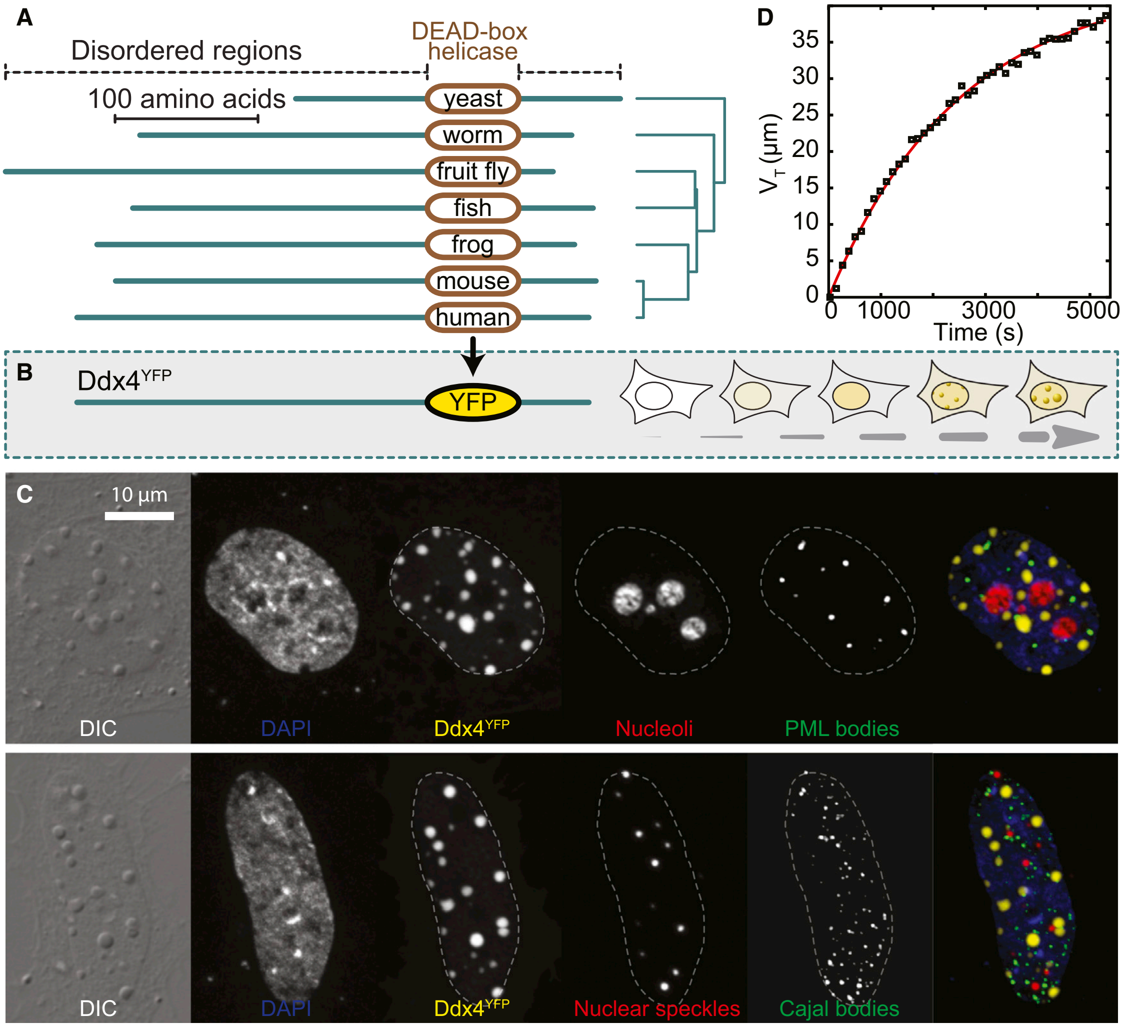

Figure 1. Ddx4 Spontaneously Self-Assembles to Form Organelles in Live Cells

(A) Evolutionary relationships between the disordered regions of Ddx4 homologs and their domain architectures. Disordered regions (green) and locations of DEAD-box helicase domains (brown) are indicated.

(B) Schematic showing the DEAD-box helicase domain of Ddx4 replaced with YFP before being transfected into HeLa cells. $Ddx4^{YFP}$ organelles appear over time.

(C) Differential interference contrast (DIC) and corresponding extended focus fluorescence intensity images of a HeLa cell expressing $Ddx4^{YFP}$. $Ddx4^{YFP}$ forms dense, spherical organelles in the nucleus. Cells were stained with antibodies to visualize nucleoli, PML bodies, nuclear speckles, and Cajal bodies as indicated, revealing that Ddx4 organelles are entirely distinct from these other bodies.

(D) The variation in total droplet volume with time is explained by the Avrami equation for nucleated growth (Supplemental Experimental Procedures Section 5). The time is measured from the appearance of the first droplet.

This epigenetically crucial nuage/chromatoid body (CB) family of membraneless organelles hosts components of an RNAi pathway, guarding spermatocytes and spermatids against the deleterious activity of transposable elements (Kotaja and Sassone-Corsi, 2007). Typical of non-membrane encapsulated organelles, nuages are generally spherical and dynamically change in number, size, and composition over their lifecycle (Meikar et al., 2011), appearing first in the juxtanuclear cytoplasm of early spermatocytes, moving toward the base of the flagellum during spermatogenesis before finally dispersing. A primary constituent of nuage is Ddx4 (Kotaja et al., 2006). In addition to a central DEAD-box RNA helicase domain that uses ATP to unwind short RNA duplexes, Ddx4 has extended N and C termini that are predicted to be intrinsically disordered (Figures 1A and S2) (Forman-Kay and Mittag, 2013).

Here, we demonstrate that human Ddx4 and its isolated disordered N terminus spontaneously self-associate both in cells and in vitro into structures that are indistinguishable from the cellular Ddx4-organelles. The mechanism for this is a phase separation commonly encountered in polymer chemistry, and the interactions that hold the organelles together are primarily electrostatic in origin. Moreover, arginine methylation, alternative splicing, and changes in ionic strength and temperature under near-physiological conditions readily dissolve the Ddx4 bodies. Two highly

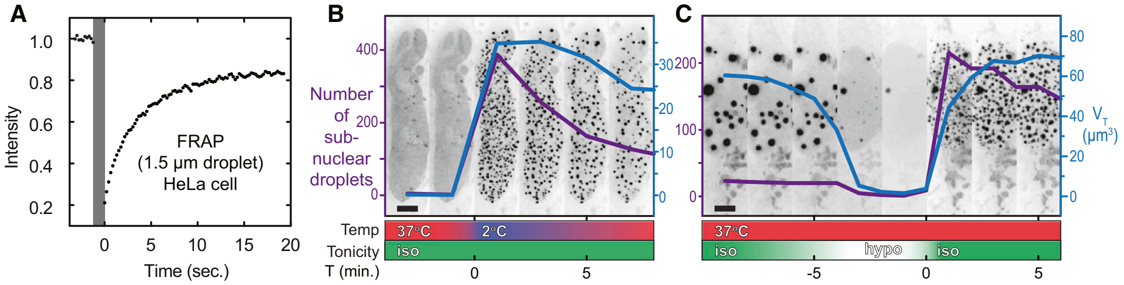

Figure 2. Ddx4^{YFP} Organelles Are Internally Mobile and Respond Rapidly to Changes in Environmental Temperature and Tonicity

(A) Fluorescence recovery after photobleaching (FRAP) of a Ddx4^{YFP} organelle in a live HeLa cell at 37°C. Sample bleaching is indicated with a gray bar. 50% of the fluorescence signal is recovered within approximately 2.5 s post-bleach, corresponding to a diffusion coefficient of $3 \pm 1 \times 10^{-13}$ m^2 s^{-1}.

(B) Cold shock induces condensation of sub-nuclear Ddx4^{YFP} droplets at low expression levels. Extended focus fluorescence intensity images showing the nucleus from a time series analysis of a HeLa cell expressing Ddx4^{YFP} undergoing cold shock. Images are shown at 2-min intervals. Prior to cold shock treatment, Ddx4^{YFP} had not reached the critical concentration for phase separation at 37°C and was diffuse in the nucleoplasm (first two frames). Rapid exchange of growth media at 37°C for media cooled on ice (time = 0) induced small Ddx4^{YFP} droplets to condense rapidly within the nucleus (purple line, number of droplets; blue line, total volume of droplets). Following cold shock, the number of Ddx4^{YFP} droplets decreased through a combination of coalescence and dissolution as the temperature rose. Scale bar, 5 µm (see Movie S2).

(C) Extended focus fluorescence intensity image slices showing a section of the nucleus from a time series analysis of a HeLa cell containing Ddx4^{YFP} droplets undergoing osmotic shock. Images are shown at 2-min intervals. Axis labels, data colors, and scale as in (B). See Movies S3 and S4.

conserved features in the sequence of Ddx4 that enable droplet formation are identified: repeating 8–10 residue blocks of alternating net charge and an over-representation of FG, GF, RG, and GR motifs within the positively charged blocks. These features are found to occur in a significant number of intrinsically disordered proteins associated with membraneless organelles. The interior of the organelles concentrate single-stranded DNA, yet largely exclude double-stranded DNA, suggesting that the bodies play a role in localizing nucleic acids. These findings lend insights into the role of intrinsically disordered proteins in the regulated spontaneous self-assembly of cellular membraneless organelles.

RESULTS

The Intrinsically Disordered Termini of Ddx4 Condense in the Nucleus of HeLa Cells to Form Organelles

In order to follow the Ddx4 disordered regions within a cell, we generated a mimic, Ddx4^{YFP}, in which the DEAD-box helicase is substituted by YFP, a fluorescent protein of similar dimensions and overall charge to the helicase domain (Figure 1A). This protein was transfected into HeLa cells, and both its expression and localization were monitored using fluorescence microscopy (Figures 1B and S2). At low expression levels, Ddx4^{YFP} was diffuse in both the nucleus and cytoplasm. As the intra-cellular concentration increased over time, dense micron-sized spherical bodies were observed to form in the nucleus that were similar in appearance to nuclear foci but physically distinct from other membraneless organelles such as the nucleoli (Figure 1C). A Ddx4 construct containing both YFP and the DEAD-box helicase domain similarly formed organelles, although they were observed to form in the cytoplasm (Figure S2C). The Ddx4^{YFP} bodies have the appearance and behavior of organelles by optical microscopy and can therefore be classified as such.

The maturation of Ddx4^{YFP} organelles was followed using time-lapsed live-cell imaging, where foci corresponding to spherical organelles of radius 0.1–1 µm could be readily identified (Movie S1). Rather than precipitating from solution in many locations at once, organelles were observed to appear individually. The growth of individual organelles within a single cell monitored over time conforms exceptionally well to that expected for Avrami nucleated particle growth (Fanfoni and Tomellini, 1998) (Figure 1D), consistent with the interpretation of the phenomenon as a phase separation involving condensation of Ddx4^{YFP} monomers into proteinaceous organelles. A more detailed analysis suggests that the number of droplets and their sizes are limited by the quantity of free monomer, a property that in principle can be closely regulated (Figure S3).

Ddx4^{YFP} Organelles Have a Dynamic Liquid-like Structure and Respond Rapidly to Changing Solution Conditions

The internal order within the organelles was assessed using fluorescence recovery after photobleaching (FRAP) measurements. The half time to recovery of the fluorescence signal of a photobleached body of diameter 1.5 µm took approximately 2.5 s at 37°C (Figure 2A), corresponding to an approximate diffusion coefficient of $3 \pm 1 \times 10^{-13}$ m^2 s^{-1}, a value two orders of magnitude lower than that measured for free globular proteins of a similar size as determined by both FRAP and NMR (Figure S5B). These self-diffusion rates are consistent with those of other non-membrane organelles, such as nuclear speckles and nucleoli (Phair and Misteli 2000). While the observed diffusion within the droplets is substantially slower than the motion of free protein, the interior is nevertheless highly mobile, consistent with weak interactions between Ddx4 proteins within the droplet.

To assess the internal structure of in situ Ddx4^{YFP} organelles and to determine if they contain, for example, fibrillar substructure, we employed electron spectroscopic imaging (ESI). This technique enables the visualization of nitrogen and phosphorous structures at the approximate resolution of 30 atoms per pixel (60 Å × 60 Å) without the use of contrast-enhancing reagents

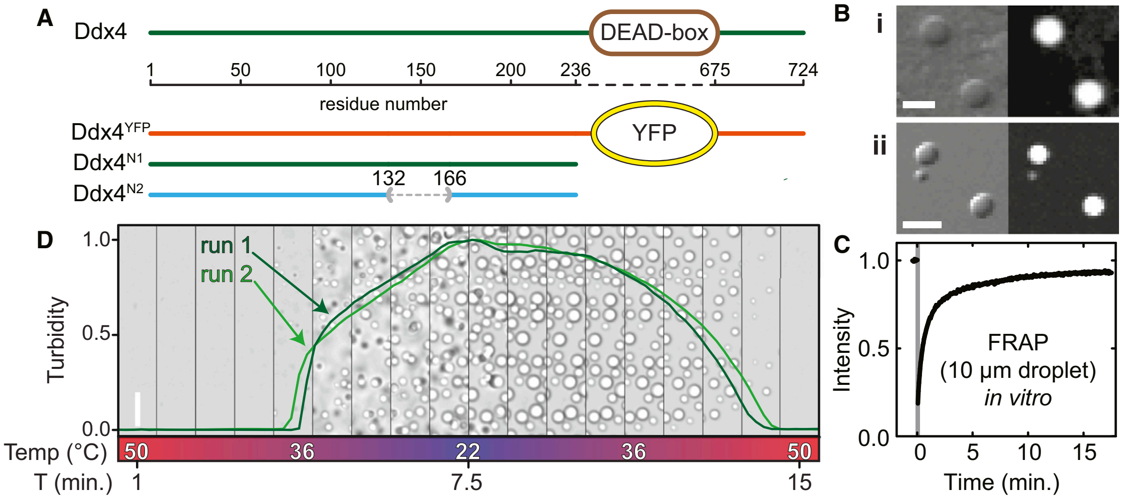

Figure 3. The N Terminus of Ddx4 Reversibly Forms Organelles In Vitro

(A) Schematic showing the relationship between constructs of Ddx4 and the wild-type protein. Ddx4^{N1} (residues 1–236) and Ddx4^{N2} contain only the disordered N terminus.

(B) DIC (left) and YFP fluorescence (right) images of (i) Ddx4^{YFP} organelles inside HeLa cells (scale bar, 2 μm) and (ii) 60:1 Ddx4^{N1}:Ddx4^{YFP} organelles formed in vitro at 150 mM NaCl (scale bar, 10 μm).

(C) FRAP curve of a 10 μm diameter droplet containing Ddx4^{N1} and recombinant, purified Ddx4^{YFP} at a molar ratio of 60:1 in 150 mM NaCl buffer at 20°C. The bleach period is indicated with the gray bar. 50% of the fluorescence signal is recovered after approximately 1 min, corresponding to a diffusion coefficient of $4 \pm 1 \times 10^{-13}$ m^2 s^{-1}.

(D) Time series analysis of bright-field microscopy images of Ddx4^{N1} (202 μM protein, 200 mM NaCl) with varying temperature, shown at 50 s intervals (scale bar, 50 μm). At 50°C, the sample was monophasic with low turbidity. Temperature was linearly decreased (4°C min^{-1}) from 50°C to 22°C. At 36°C, the turbidity of the sample rapidly increased concomitant with the emergence of an incipient dense phase containing concentrated Ddx4^{N1}. After holding at 22°C for 1 min, the sample was reheated to 50°C. At approximately 45°C during reheating, the condensed phase was completely dissolved and the turbidity of the solution returned to its initial turbidity. The thermal cycle was repeated with the same sample in situ (light green line), revealing that the changes in the droplet are fully reversible.

required for conventional TEM experiments. At this resolution, there was no significant variation in density through the organelles, as would be expected if the structure were based on amyloid fibrils (Kato et al., 2012; Kim et al., 2013). The organelles are clearly partitioned from surrounding nuclear structures including chromatin domains and other native nuclear bodies (Figures 1C and S2B), a finding consistent with the liquid droplet-like nature suggested by FRAP measurements (Figure 2A).

Ddx4^{YFP} organelles were exposed to cellular environments varying in both temperature and tonicity to elucidate factors that dictate their stability. Cells transfected with Ddx4^{YFP} at low expression levels where no organelles were observed were subjected to cold shock by rapidly transferring them from 37°C to 2°C. This caused immediate condensation of Ddx4^{YFP} organelles (Figure 2B and Movie S2). As the temperature of the cells was subsequently allowed to rise, the number (purple) and total volume (blue) of organelles decreased as Ddx4^{YFP} protein dissociated from organelles and dispersed into the nucleoplasm. Similarly osmotic shock, induced by a rapid change from isotonic (150 mM ionic strength) to hypotonic conditions (~150 μM ionic strength) caused the rapid dissolution of Ddx4^{YFP} droplets. These immediately reformed upon return to isotonic conditions (Figure 2C and Movies S3 and S4). These data reveal that the disordered termini of Ddx4 mediate self-organization into macroscopic fluxional spherical structures in live cells that can be classified as organelles, which can respond rapidly to changes in the cellular environment.

Ddx4 Disordered Regions Reversibly Form Structures In Vitro that Are Indistinguishable from Those Formed in Cells

To gain insight into the transitions observed in cells, we expressed and purified Ddx4^{YFP} recombinantly. In addition, two further polypeptides were also produced, corresponding to the N-terminal disordered region (residues 1–236), Ddx4^{N1}, and residues 132–166 exchanged for a single aspartate, Ddx4^{N2}, corresponding to a naturally occurring splice variant (Figure 3A). The properties of the dispersed Ddx4^{N1} and Ddx4^{N2} were interrogated using solution-state NMR. The narrow range of amide proton chemical shifts observed in a $^{1}H/^{15}N$ HSQC spectra of the two proteins (Figure S5A) confirmed that they are intrinsically disordered. The hydrodynamic radii (R_h) of the constructs, determined using pulsed field gradient NMR experiments, were found to fall between 29 and 33 Å ($6.3–7.1 \times 10^{-10}$ m^2s^{-1} Figure S5B). This size is closer to that predicted for a folded protein of this length (23 Å) rather than an unfolded protein (50 Å) of this length (Marsh and Forman-Kay, 2010; Wilkins et al., 1999), indicating that the Ddx4 N-terminal intrinsically disordered region maintains transient tertiary contacts that keep the protein compact (Dedmon et al., 2005).

Under near-physiological conditions of ionic strength 150 mM, 37°C, solutions of 100 μM Ddx4^{N1} and Ddx4^{YFP} rapidly became turbid. When droplets in the turbid phase were imaged, their morphologies and (qualitatively) their distribution of particle size and time dependence mirrored that seen within cells,

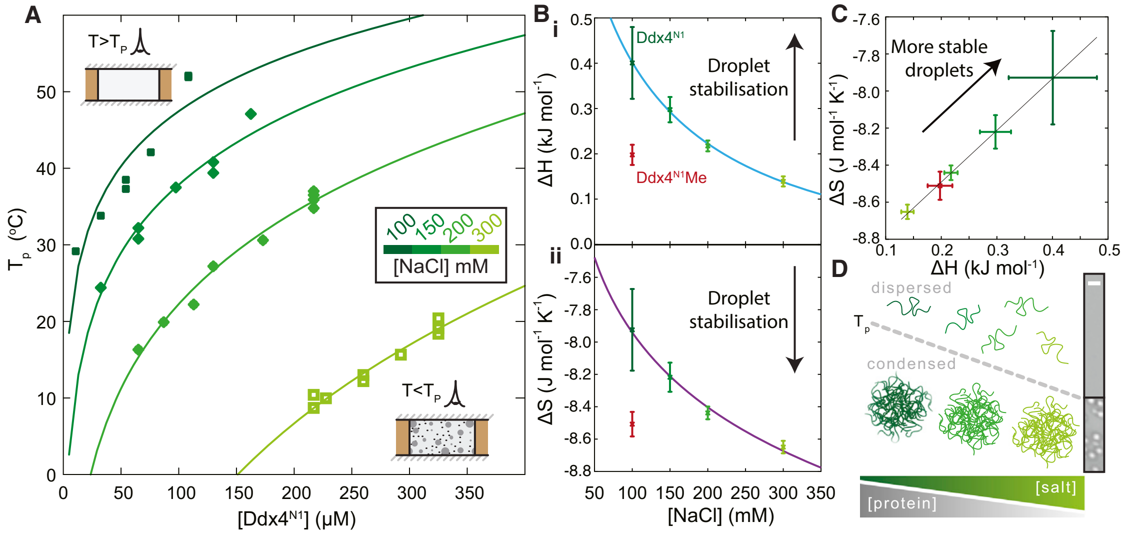

Figure 4. Quantitative Analysis and Interpretation of the Ddx4^{N1} Phase Transition

(A) The temperature at which the phase transition is observed, T_P, was determined as a function of protein concentration and ionic strength at pH 8. At a given ionic strength, the Flory-Huggins model of polymer phase separation quantitatively describes each curve. This yields two fitting parameters, the enthalpy and entropy changes of the transition, which report on the microscopic interactions between molecules.

(B) The interaction parameters varied in a predictable way with increasing salt. The enthalpic contribution to the interaction parameter (i) was found to decrease as a function of increasing NaCl. This is quantitatively explained by fitting the curve to a screened coulomb potential (light blue, Equation S19). The non-ionic component of the enthalpy is close to zero, -0.058 ± 0.137 kJ mol^{-1}, the relative permittivity within the condense phase was 45 ± 13, and the average spacing between opposite charges is 13 ± 2 Å. The entropic contribution to the interaction parameter (ii) decreases slightly with increasing salt, fitted to Equation S20. The error bars represent the SE in the fitted parameters (Figure 4A).

(C) The entropy and enthalpy values are correlated, suggesting that when the interactions are destabilized at higher salt, the chains in the interior of the droplet become more mobile. The error bars represent the SE in the fitted parameters (Figure 4A).

(D) Schematic representation of dissolution of the Ddx4 condensed phase and expansion of the monomer in the disperse phase through increasing ionic strength or temperature. Ddx4^{N1} protein chains depicted as green lines. Transition point (T_p) is indicated with a dashed gray line. The ionic interactions within the droplets are attenuated with increasing salt, as is the residual structure within the protein in the dispersed phase. Corresponding bright-field images are shown on the right. Scale bar, 10 μm.

indicating that we can readily recapitulate the organelles in vitro (Figure 3B). By contrast, under identical conditions, the splicing variant Ddx4^{N2} remained soluble, revealing that alternative splicing can regulate the formation of organelles.

As the droplets formed in vitro appeared identical in form to those observed in cells, FRAP experiments were performed to determine if they also have similar physical characteristics and internal structure. Using Ddx4^{YFP} as a tracer in droplets otherwise composed of Ddx4^{N1} (molar ratio of 1:60), 50% of fluorescence signal intensity was recovered after approximately 1 min following photobleaching of a 10 μm diameter dense-phase droplet (Figure 3C) corresponding to a diffusion coefficient of $4 \pm 1 \times 10^{-13}$ m^2 s^{-1}. Within experimental uncertainty, this is identical to the value observed directly within live cells ($3 \pm 1 \times 10^{-13}$ m^2 s^{-1}; Figure 2A), indicating that the internal structure and internal dynamics of the organelles formed both in vivo and in vitro are highly similar.

Since Ddx4^{YFP} organelles could be induced in cells by cold shock (Figure 2B), the in vitro organelles were subject to thermal perturbation. Using bright-field microscopy and a thermal stage, a fully dispersed solution of Ddx4^{N1} (pH 8.0) at 50°C was cooled at 4°C min^{-1} to 22°C. At 36°C, the solution became turbid and droplets were observed to condense (Figure 3D). After equilibration at 22°C for 1 min, the sample was reheated to 50°C. As the temperature was raised, the droplets were observed to dissolve. Multiple cycles were repeated, revealing that the process is reversible (Figure 3D). In both respects, the thermal cycle was highly similar to that observed within cells.

Ddx4^{N1} Organelles Are Stabilized Predominantly by Electrostatic Interactions

In the case where molecular chains attract each other, polymer theory anticipates that, at high concentrations and low temperatures, they will phase separate and form condensed droplets suspended in solvent. By contrast, at high temperature, the translational entropy of the free polymer will dominate and the polymer will mix with solvent. As the temperature is lowered, a "bimodal" or "cloud point" is reached, T_P, where favorable interactions overcome the translational entropy loss, and droplets of pure polymer will condense via a nucleated mechanism, as quantitatively described by Flory-Huggins theory of phase separation (Flory, 1942; Huggins, 1942). We measured T_P for Ddx4^{N1} (Figure S4) as a function of protein concentration and ionic strength, enabling the construction of a phase diagram (Figure 4A, points). At all ionic strengths examined, T_P increases with increasing Ddx4^{N1} concentration in a manner that is well predicted by Flory-Huggins theory (Figure 4A, solid lines). The transition temperatures were found to decrease as ionic strength

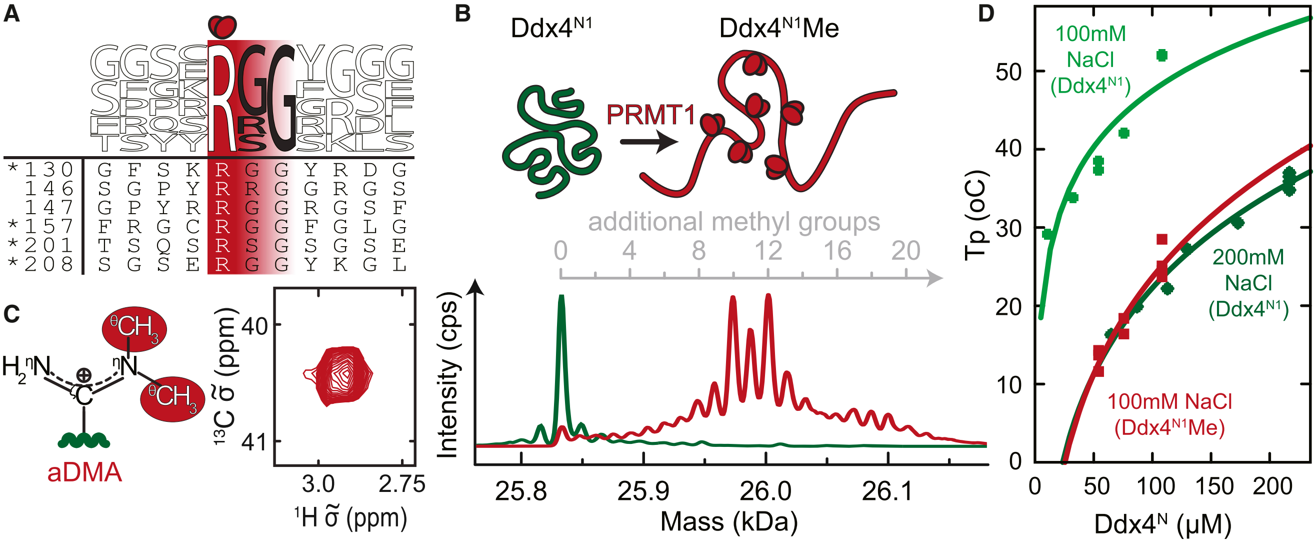

Figure 5. Post-Translational Modification by Arginine Methylation Alters the Phase Transition of Ddx4^{N1}

(A) Sequence logo (weblogo.berkeley.edu) depicting the amino acid motifs surrounding arginine residues of Ddx4^{N1} predominantly targeted by PRMT1. Arginine residues to be converted to aDMA are highlighted in dark red and with two small ellipses. The amino acid numbers of the modified arginine residues are shown within their respective sequence contexts. Asterisks highlight aDMA sites identified in Ddx4^{N1}Me with 95% probability (Scaffold score) from a combination of trypsin and GluC digestion of recombinant, purified Ddx4^{N1}Me. aDMA at sites 146 and 147 was identified at ~65% probability (Scaffold score).

(B) Schematic and mass reconstruction of +TOF MS spectra of Ddx4^{N1} (green; 25.833 kDa) and Ddx4^{N1}Me (dark red). In the latter, a series of peaks was observed between 1 and 20 methyl additions. The major peaks indicate complete aDMA modification at 5 and 6 sites, respectively.

(C) A schematic of aDMA together with an insert showing the ^{1}H-^{13}C HSQC NMR spectrum of the $^{\theta}CH_3$ of Ddx4^{N1}Me. The chemical shifts of the methyl groups verify that the modification is aDMA (see Figure S5).

(D) The phase-transition temperatures of Ddx4^{N1}Me (dark red) are shifted compared to the unmodified form under the same conditions (light green). Modification with aDMA at a mixture of 5–6 aDMA sites reduces the transition temperature by 25°C, an effect on the phase transition comparable to increasing the ionic strength by 100 mM.

was increased, indicating that the interactions between the protein molecules within the condensed phase have a strong electrostatic component. Flory-Huggins theory (Flory, 1942; Huggins, 1942) was found to quantitatively explain the scaling of T_P with increasing protein concentration, enabling characterization of how the enthalpic and entropic interaction terms vary with ionic strength (Figure 4A).

The scaling of the enthalpic part of the free energy can be quantitatively explained by assuming that the individual interactions between protein molecules within the droplet can be represented by a screened coulombic (electrostatic) potential (Shammas et al., 2011) such that increasing salt predictably attenuates the interaction (Equation S19). Fitting this model to the data (Figure 4Bi) yields an average separation between interacting charges of 13 ± 2 Å and the relative permittivity, or dielectric, of the droplet of 45 ± 13. This value is smaller than that of bulk water (80) (Figure 4Bi), suggesting that electrostatic interactions will be less screened than in bulk water. It is interesting to compare this value to those of the hydrophobic interior of folded proteins (4), of the interior of a lipid bilayer (2–4), and of polar organic solvents such as acetonitrile (38) and DMSO (47). The favorable interactions are effectively entirely screened at high salt, indicating that electrostatic forces are responsible for droplet stability.

The entropic part of the free energy was found to favor droplet formation under all conditions, suggesting that the residual order in either the dispersed protein or its associated solvent molecules is substantially reduced upon condensation into droplets. Both entropy and enthalpy changes are correlated (Figure 4C) tentatively, suggesting that as interactions are weakened with salt the interior becomes more mobile. Taken together, we can conclude that the protein droplets are held together primarily by electrostatic interactions. These findings reveal that the responsiveness of organelles in the cell to changing environmental conditions (Figure 4D) stems from the microscopic interactions between individual protein chains.

Arginine Methylation Destabilizes Ddx4^{N1} Organelles

Ddx4 and other nuage proteins are regulated through the methylation of multiple arginine sites (Chen et al., 2009; Kirino et al., 2010). In the case of the Piwi proteins, arginine methylation generates binding sites for Tudor domain-containing binding partners, thereby co-localizing them with the nuage (Liu et al., 2010). In mammalian cells, the enzyme PRMT1 catalyzes the addition of two methyl groups to one of the guanidine nitrogen atoms of the arginine side chain in predominantly RGG motifs (Wooderchak et al., 2008), converting it to asymmetric dimethyl arginine (aDMA) (Tang et al., 2000) (Figure S5E). Ddx4^{N1} is post-translationally modified at multiple sites by PRMT1 in vivo (Kirino et al., 2010) and contains six predicted methylation sites (Figure 5A). By co-expressing Ddx4^{N1} with PRMT1 in *E. coli*, between 1 and 20 methyl groups were added, with the majority of

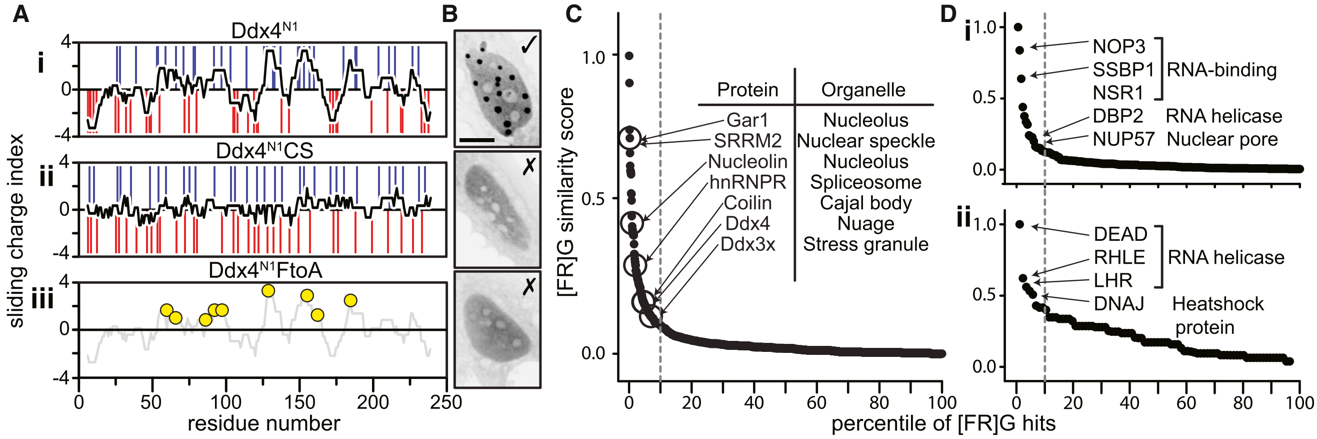

Figure 6. The Sequence Features that Enable Droplet Formation by Ddx4 and Their Distribution within the Human Genome

(A) Sliding net charge (10 amino acid window, black) is shown for (i) Ddx4^{N1} and (ii) a charge-scrambled mutant, Ddx4^{N1}CS, obtained by swapping the positions of positive residues (blue bars) and negative residues (red bars) to minimize any persistence of blocks of charge. (iii) A mutant where nine phenylalanine residues, whose placement was highly conserved, were mutated to alanine (Ddx4^{N1}FtoA, see Figure S6). The positions of the nine phenylalanine residues (yellow circles) mutated to alanine are indicated.

(B) Representative fluorescence images from cell imaging experiments reveal that Ddx4^{N1}CS and Ddx4^{N1}FtoA do not form organelles in cells under physiological conditions. Residual HeLa nucleoli are still observed as fluorescence-depleted regions within the cell nucleus.

(C) The human genome was surveyed for sequences with similar physical properties to the Ddx4 disordered termini. 1,556 sequences out of 14,198 were identified to have [F/R]G spacings in their sequence that are similar to the Ddx4 ortholog family. The top 10% of these are indicated (dotted line). A significant number of proteins associated with forming non-membrane organelles were present in this group.

(D) Similar plots from the yeast (i) and *E. coli* (ii) genomes revealing a number of proteins closely associated with nucleic acid biochemistry.

chains containing 10 and 12 additional methyl groups (Figure 5B). Solution-state NMR confirmed that the dominant modification was aDMA (Figures 5C and S5), and the locations of the sites were confirmed by proteolytic cleavage and fragmentation mass spectrometry. Taken together, the majority of protein molecules had either 5 or 6 aDMA-modified arginine residues in the predicted sites (Figure S5). Remarkably, methylation of this type significantly destabilized the droplets, lowering the transition temperature by 25°C (Figure 5D). The extent of the destabilization of the droplets is the equivalent of adding 100 mM of additional salt to Ddx4^{N1}. Post-translational modification is therefore revealed to be a mechanism through which droplet formation can be attenuated under physiological conditions and an effective method to regulate distinct subcellular microenvironments.

Patterns of Charged Residues Are Required for Organelle Formation

The propensity for polypeptides to spontaneously phase separate under physiological conditions is not a property common to all intrinsically disordered proteins. To identify which features within the sequence confer this ability, we analyzed the disordered tails of orthologous Ddx4 proteins to understand what distinguishes them from other intrinsically disordered proteins. Droplet formation both in cells and in vitro was highly sensitive to ionic strength, indicating that the charged residues confer significant stability to the droplets. While the number of hydrophobic residues is lower in Ddx4 proteins than the values for an "average" intrinsically disordered protein (Figure S6A), the proportion of charged residues in Ddx4 orthologs (25%) is very close to the average number of charged residues in all IDPs surveyed (26.1%), revealing that the predictors are more complex.

A notable feature of Ddx4 disordered termini is that they arrange their charged residues into clustered blocks of net positive and negative charge (Figure 6Ai), resembling a block copolymer. The clusters persist for approximately 8–10 residues in length and tend to contain 3–8 similarly charged residues. To determine the physical importance of this charge patterning, we produced a Ddx4 variant, Ddx4^{N1}CS, with the same overall net charge, but in which the blocks were scrambled (Figure S6G). In Ddx4^{N1}CS, the regions of opposing charge are removed while simultaneously maintaining the same overall isoelectric point (PI), amino acid composition, and positions of all other residues (Figure 6Aii). This construct was unable to form organelles in vitro under near-physiological conditions. When expressed in cells in the charge-scrambled form of Ddx4^{YFP}, the protein accumulated to high concentrations without forming organelle-like structures (Figure 6Bii), revealing the importance of charge patterning in organelle formation.

Repeated FG and RG Spacing in Ddx4 N Termini Suggest Cation-Pi Interactions Contribute to Droplet Stability

To ascertain specific sequence features that contribute to droplet stability, we looked for over-representation of amino acid pairs in the disordered regions of Ddx4 orthologs when compared to their background proteomes (Supplemental Experimental Procedures). We found that both GF and FG groupings were both significantly over-represented in human Ddx4 and a common feature of the Ddx4 family of orthologs (Figure S6B, dot size and color, respectively). Closer inspection of the linear sequences of Ddx4 proteins revealed that FG and GF motifs

were clustered within positively charged blocks, typically close to arginine residues in the form of either RG or GR dipeptides. We undertook an analysis to determine the relative locations of these residues within the Ddx4 disordered termini to ascertain whether the spacing between these repeats was statistically significant. To conduct this analysis, we first measured the sequence distance between all F-F, R-F, F-R, and R-R residues within the Ddx4 ortholog families, including only F/R residues that are immediately followed or preceded by a G. For example, FGxxxxGR would be recorded as an F-R spacing of 7 residues (Figure S6C). The counts were then normalized to those of the background sequences to ensure that any occurrence is a significant property of the Ddx4 family and not an intrinsic property of disordered proteins. Strikingly, the test revealed a significant statistical trend for FG and GF pairs to be spaced by 8–11 residues apart in Ddx4 disordered termini and RG and GR pairs to be spaced 4 residues apart (Figure S6C). Similar patterns were observed in the F-R distances. When the analysis was extended to include groups of three or more dipeptide repeats, similar, but more pronounced, trends were observed in the spacing of multiple [F/R]G dipeptides (Figure S6D). Taken together, it would appear that there has been evolutionary pressure acting on Ddx4 that holds the relative spacing of [F/R]G pairs within a well-defined window.

Of the 14 F residues within the sequence, this method identified ten within $Ddx4^{N1}$ as having their relative positions conserved by evolution with respect to R and other F residues. Of these, nine were present within positively charged blocks (Figure 6Aiii). To test the physical significance of these residues to droplet formation, a construct was produced where these nine residues were mutated to alanine ($Ddx4^{N1}$FtoA; Figure 6Aiii). Notably, $Ddx4^{N1}$FtoA was unable to induce droplet formation either in cells or in vitro (Figure 6Biii). Finally, the strength of the quadrupole in the aromatic ring of the phenylalanine residues was reduced by enrichment with 3-fluorophenylalanine, $Ddx4^{N1}$F (Figure S6Fi). The organelles were significantly destabilized (Figure S6Fii), confirming the importance of these aromatic residues and suggesting that cation-pi interactions are required for organelle formation.

Sequence Determinants of Ddx4 Droplet Formation Are Found in Other Organelle-Forming Proteins

The statistical map of FG and RG proximities can be considered a "fingerprint" of organelle-forming features of Ddx4 (Figure S6D). Using this as a reference, we interrogated the human proteome (Yaffe et al., 2001), identifying 1,566 similar sequences (Figure 6C). After ranking the scores of the sequences, we identified a sharp increase in the scoring function that occurred for the top 10%. Interestingly, this group of 156 sequences included a number known to be primary constituents of non-membrane encapsulated organelles, such as Nucleolin and Gar1 (nucleolus), Coilin (Cajal body), hnRNPs (splicing speckles), and Ddx3x (stress granules) (Figure 6C). These results strongly suggest that the sequence properties identified in Ddx4 enabling organelle formation via a phase separation mechanism are general features of organelle-forming proteins. Moreover, similar patterns were observed in a subset of proteins associated with RNA processing in both yeast and *E. coli* genomes, a function commonly localized to membraneless organelles (Figure 6D).

We performed an analysis of the gene ontology (GO) terms in the UniProt database for the top 10% of our ranked sequences. Significantly, these sequences were found to be generally localized in membraneless cellular compartments, such as nuclear bodies, nuclear speckles, the spliceosome, and nucleolus, and involved in associated biological processes, such as RNA processing, chromatin organization, and methylation in both human and yeast genomes (Figure S6E). Moreover, there were a large number of proteins related to cell adhesion found in the extracellular matrix. This observation is remarkable in light of the finding that several of these proteins have been observed to coacervate (Yeo et al., 2011). These results identify a class of intracellular proteins involved in organelle formation and cell-cell adhesion that contain blocks rich in FG/RG repeats and likely form phase-separated structures in vivo.

Ddx4 Droplets Differentially Solubilize Nucleic Acids

Ddx4-containing nuage and other membraneless organelles are frequently associated with nucleic acid biochemistry. Thus, we might expect them to differentially bind and concentrate various nucleic acids as well as other biomolecules. To test this, we prepared a 32 base single-stranded and 32 base-paired double-stranded DNA labeled with the fluorescent dye atto657N and mixed them with Ddx4 organelles. The relative fluorescence in the two phases indicates the relative concentration, and thus solubility, of the DNA in the two contrasting environments. While the double-stranded DNA was largely excluded from the droplets, the single-stranded DNA was concentrated significantly in the interior of the droplets (Figure 7). This result suggests that the interior of Ddx4 organelles provides a substantially different environment to the aqueous cellular interior in order to preferentially solubilize and concentrate certain types of biomolecules.

DISCUSSION

Ddx4 Organelles Are Phase-Separated Droplets

Membraneless organelles such as the nucleolus, nuclear speckles, Cajal bodies, P bodies, and stress granules share many properties (Brangwynne, 2011; Phair and Misteli, 2000). They are resolvable by microscopy, are generally spherical, are internally dynamic, and are composed of a well-defined set of proteins. The disordered N terminus of Ddx4, a primary component of nuage organelles, can self-associate to form bodies indistinguishable from these by optical microscopy, both in cells and in vitro. These condensed droplets have a liquid-like interior, consistent with maintenance of disorder and lack of observed discrete fibrillar or other ordered structure. Their mechanism of formation and thermodynamic properties reveal that these bodies are a condensed phase, distinct from the aqueous background, with the availability of free protein likely determining both the number of organelles and their size distribution through growth kinetics.

Consistent with this picture, droplet formation is readily reversible and responsive to changes in environmental conditions. Increasing the ionic strength or temperature, methylating

CellPress

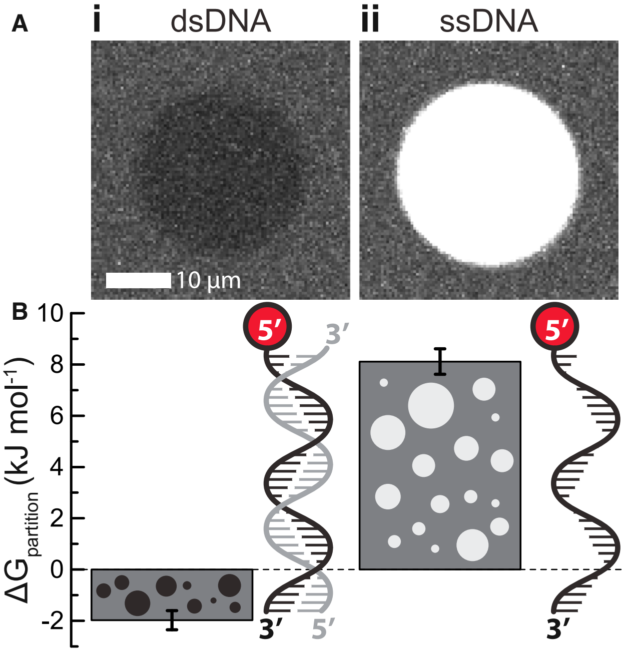

Figure 7. Proteinaceous Organelles Differentially Solubilize Nucleic Acids

(A) Ddx4^{N1} organelles were allowed to form under near-physiological conditions at a total concentration of 162.5 μM. (i) Double- and (ii) single-stranded 32-nt DNAs (dsDNA and ssDNA, respectively) tagged with atto647N were added at a concentration of 1 μM. In the case of dsDNA, the majority of the material was excluded from the droplets. The reverse effect was observed for ssDNA.

(B) The average and SD (error bar) confocal fluorescence emission intensities from both inside and outside the organelles were used to quantify the partition equilibrium coefficient and its corresponding free energy (Equation 1, Figure S7).

certain significant arginine residues, and disrupting the charged blocks by alternative splicing can lead to complete dissolution of the droplets. As the functions performed by these organelles are both cell-type specific and temporally regulated, these are means by which the droplets can be regulated in vivo. These organelles are effectively liquid droplets of condensed disordered protein that form an intra-cellular compartment that is distinct from the aqueous background (Brangwynne, 2009, 2011).

Ddx4 Organelles Are Held Together Primarily by Electrostatic Interactions

Intrinsically disordered proteins experience a higher rate of sequence alteration through evolution than globular proteins (Brown et al., 2011), yet we find two clear sequence features to provide the required electrostatic interactions. Clusters of opposing charge of length 8–10 residues are required for droplet formation together with both FG and RG pairs held in close sequence proximity. It is likely that the F/R residues are engaged in cation-pi interactions, as frequently observed in the context of folded proteins (Gallivan and Dougherty, 1999). Both increased levels of salt and arginine methylation would disrupt quadrupolar interactions, as we observe.

The specific placement of certain residue types has recently been identified as crucial for forming self-assembled structures with desirable physical properties. In the cases of assembly of EWS/FUS proteins, P bodies, and the nuclear pore complex, formation is facilitated by repeated occurrences of the dipeptides YG/RG, QN, and FG, respectively (Balagopal and Parker, 2009; Decker et al., 2007; Frey et al., 2006; Kato et al., 2012; Sun et al., 2011). While individual interactions are relatively weak, careful placement of many such multivalent interactions can lead to the self-assembly of otherwise disordered polypeptide chains (Li et al., 2012).

The Ddx4 sequences are found to conform to a "fingerprint" that consists of FG and RG groups arranged in a distinct pattern. A wider screening of the human, yeast, and *E. coli* genomes reveals that many intrinsically disordered proteins share this pattern, the majority of which are associated with non-membrane encapsulated organelles and extra-cellular adhesion proteins. This indicates that the sequence features we have identified for Ddx4 organelle formation may be more general for formation of membraneless organelles and that prokaryotic organisms may also utilize such proteinaceous bodies for compartmentalization.

Ddx4 Organelle Formation Is Distinct from Amyloid Formation

It is interesting to compare phase separation of Ddx4 proteins into organelles with the aggregation of proteins more generally into the amyloid fibrils that are associated with misfolding conditions, including Alzheimer's and Parkinson's diseases (Dobson, 2003). Their morphologies and internal features easily distinguish the two types of aggregates, as the former is spherical with internal mobility, whereas the latter are fibrillar with constituent monomers that adopt a precise structural arrangement with similar interactions (Baldwin et al., 2011; Fitzpatrick et al., 2013; Tycko, 2011). Both aggregation mechanisms require only that there is favorable free energy for monomer association and both processes are reversible (Baldwin et al., 2011). In the case of amyloid fibril formation, monomers interact predominantly by backbone hydrogen bond interactions between adjacent β sheets, a property likely to be generic to all polypeptide chains (Dobson, 2003). By contrast, the formation of Ddx4 organelles requires patterned electrostatic interactions, suggesting it is not likely to be so widespread. It would be highly undesirable to have uncontrolled protein association into micron-sized bodies and, as for amyloid formation (Monsellier and Chiti, 2007), the majority of the proteome has likely experienced negative selection to remove traces of sequence determinants where they are deleterious.

Ddx4 Organelles Are Likely Minimally Stable to Enable Regulation by PTMs

The folded structures of proteins have been recognized as being minimally stable, a property that facilitates the relative motion of domains and enables function (Bryngelson et al., 1995). For Ddx4, small changes at the level of individual chains can result in organelle dissolution under physiological conditions, indicating that they too are minimally stable, perhaps to prevent uncontrolled growth. This renders them susceptible to regulation

by small perturbations, for example by methylation of arginines by PRMT1, a modification observed in vivo (Chen et al., 2011). Another regulatory mechanism that exploits the finely tuned stability utilizes alternative splicing of Ddx4 to substitute residues 132–166 with a single aspartate, effectively removing an entire positively charged "block" from the sequence and rendering it unable to form organelles under physiological conditions.

Other membraneless organelles have been shown to have similarly finely tuned stabilities under physiological conditions. FUS is induced to self-associate and form granules in the cytoplasm following arginine methylation by PRMT1 (Yamaguchi and Kitajo, 2012). SR proteins enter nuclear speckles for engagement in RNA splicing in the event of phosphorylation, and phosphorylation of coilin is correlated with Cajal body formation (Hearst et al., 2009; Misteli et al., 1998). SUMOylation of PML is required for PML nuclear body formation, and de-SUMOylation allows constituent proteins to be released and bodies to be broken apart during mitosis (Dellaire et al., 2006a, 2006b). Our results together with these observations suggest that PTMs, by affecting both self-association and co-localization with other binding partners, provide a powerful mechanism of dynamic and responsive regulation of organelle formation.

Ddx4 Organelles Provide an Alternative Phase for Biochemical Processes

By exploiting differential intermolecular interactions between the bulk aqueous solvent and the interior of the droplets, Ddx4 organelles effectively offer a disordered protein phase as an alternative solvent environment for biomolecules. In this case, the organelle phase can largely exclude double-stranded DNA, yet concentrate single-stranded DNA, acting as a molecular filter. It is likely that Ddx4 binds to single-stranded DNA using the same cation-pi interactions that appear to drive self-association, as observed in the context of folded proteins mediating interactions with single-stranded nucleic acids (Gromiha et al., 2004; Morozova et al., 2006). Many membraneless organelles are associated with RNA processing functions, strongly suggesting that this unexpected property of the organelles is functionally relevant. It is a common strategy for an organic chemist to perform reactions in different solvents depending on the reaction required, and so it is interesting to consider a similar process occurring in vivo.

Conclusion

Here, we demonstrate that intrinsically disordered regions of Ddx4 reversibly phase separate to form droplets both in live cells and in intro via a mechanism encountered frequently in polymer chemistry. These organelles lack an internal structure and are highly fluid, effectively creating a separate solvent from the bulk aqueous environment of the cell, with unique biochemical properties. The interactions that stabilize the droplets are primarily electrostatic in origin and are readily modified by alternative splicing, arginine methylation, changes in ionic strength, and temperature, providing a means for organelle regulation. The sequence characteristics that enable Ddx4 droplet formation are found to be present in a large number of disordered proteins associated with membraneless organelles. Results strongly suggest that phase separation of specific disordered proteins to form organelles is a widespread phenomenon, providing an elegant and dynamically responsive strategy for biological compartmentalization.

EXPERIMENTAL PROCEDURES

Genes for Ddx4 proteins were synthesized by GenScript and expressed recombinantly in *E. coli*. Methylated $Ddx4^{N1}$, $Ddx4^{N1}Me$, was produced by co-expression with PRMT1. For NMR analysis, isotopically enriched samples were prepared by growing the appropriate sample in M9 media enriched in ^{15}N or ^{13}C reagents as required (Supplemental Experimental Procedures Section 1). HeLa cells were cultured on MatTek dishes and transfected with Ddx4 variants using the Effectene (QIAGEN) or polyethylenimine (PEI) methods. Cold shock, osmotic shock, and organelle growth experiments were performed on a Leica DMIRE2 inverted microscope equipped with a live cell chamber. Image z stacks recorded at each time point were de-convolved using an appropriate point-spread function, and automated corrections for photobleaching, sample movement, and contrast enhancement were performed using Volocity software. $Ddx4^{YFP}$ organelles were identified by having significant intensity in the microscopic images (>6 SD in pixel intensity more than the background) and were tracked using the Volocity software. The total organelle volume (Figure 1D) was fitted to the Avrami equation (Fanfoni and Tomellini, 1998) for nucleated growth (Supplemental Experimental Procedures Section 5).

FRAP experiments both in vitro and in vivo were performed in a live cell chamber mounted on an Olympus IX81 inverted microscope. The effects of bleaching at 515 nm on the emission intensity at 527 nm were followed, and the recovery of intensity was analyzed using the diffusion equations of Fick (Supplemental Experimental Procedures Section 2).

The transition temperature was measured using a Linkam THMS600 thermal stage mounted on an Olympus BX61 microscope. Sealed sample chambers containing protein solutions comprised coverslips sandwiching a SecureSeal imaging spacer (Sigma) and were mounted on the THMS600 silver heating/cooling block. The variance in the solution conditions was monitored with temperature. The data were analyzed using Flory-Huggins theory as described in the Supplemental Information to obtain the binodal phase temperature, giving estimates for ΔH and ΔS, the specific enthalpy and entropy changes induced by the interaction (Supplemental Experimental Procedures Section 6).

Statistical analysis of the Ddx4 family included 68 orthologous proteins from 46 species, with remaining intrinsically disordered regions of proteins in the genomes being used as a reference state. The sliding charge score was calculated as the net charge in a 10-residue window, normalized by the probability of finding that charge window within the reference proteins. Similarly, the spacing of FG/GF/RG/GR motifs was calculated as the significance of occurrence in the Ddx4 orthologous set, normalized by the significance of occurrence in the background set (Supplemental Experimental Procedures Section 7). Having defined a position-specific "fingerprint" of the arrangement of these motifs from $Ddx4^{N1}$, we used it to find similar proteins in human, yeast (*S. cerevisiae*), and *E. coli* (K12) genomes.

For DNA uptake measurements, double- and single-stranded DNA (see Supplemental Information) was coupled to Atto647N (Atto-Tec) through a dT-C6 linker. DIC and fluorescence images were obtained after preparing organelles as described for the T_P experiments, with a total protein concentration of 162.5 μM and nucleic acid concentration of 1 μm. The partition free energy was defined as

$$K_{partition} = \frac{[DNA]^{outside}}{[DNA]^{inside}} = \frac{\overline{E}^{outside}_{+DNA} - \overline{E}^{outside}_{empty}}{\overline{E}^{inside}_{+DNA} - \overline{E}^{inside}_{empty}}, \qquad \text{Equation 1}$$

where E is the average emission intensity in the specified phase after excitation at 635 nm (Supplemental Experimental Procedures Section 8).

SUPPLEMENTAL INFORMATION

Supplemental Information includes Supplemental Experimental Procedures, seven figures, and four movies and can be found with this article online at http://dx.doi.org/10.1016/j.molcel.2015.01.013.

ACKNOWLEDGMENTS

We are grateful to Harald Stöver for use of the thermal microscope stage, Lewis Kay and Ranjith Muhandiram for their NMR expertise, Sarang Kulkani for assistance with microscopy experiments, and Jonathan Doye. All provided insightful discussion. The work was funded through grants from the Canadian Institutes of Health Research (MOP-6849) and the Ontario Research Fund (T.P.), the Canadian Cancer Society Research Institute (J.D.F.-K.), and the BBSRC (A.J.B.). We dedicate this paper to the inspirational scientist and truly remarkable individual, Tony Pawson.

Received: January 23, 2014
Revised: May 12, 2014
Accepted: December 29, 2014
Published: March 5, 2015

REFERENCES

Balagopal, V., and Parker, R. (2009). Polysomes, P bodies and stress granules: states and fates of eukaryotic mRNAs. Curr. Opin. Cell Biol. *21*, 403–408.

Baldwin, A.J., Knowles, T.P., Tartaglia, G.G., Fitzpatrick, A.W., Devlin, G.L., Shammas, S.L., Waudby, C.A., Mossuto, M.F., Meehan, S., Gras, S.L., et al. (2011). Metastability of native proteins and the phenomenon of amyloid formation. J. Am. Chem. Soc. *133*, 14160–14163.

Brangwynne, C.P. (2011). Soft active aggregates: mechanics, dynamics and self-assembly of liquid-like intracellular protein bodies. Soft Matter *7*, 3052–3059.

Brangwynne, C.P., Eckmann, C.R., Courson, D.S., Rybarska, A., Hoege, C., Gharakhani, J., Jülicher, F., and Hyman, A.A. (2009). Germline P granules are liquid droplets that localize by controlled dissolution/condensation. Science *324*, 1729–1732.

Brangwynne, C.P., Mitchison, T.J., and Hyman, A.A. (2011). Active liquid-like behavior of nucleoli determines their size and shape in Xenopus laevis oocytes. Proc. Natl. Acad. Sci. USA *108*, 4334–4339.

Brown, C.J., Johnson, A.K., Dunker, A.K., and Daughdrill, G.W. (2011). Evolution and disorder. Curr. Opin. Struct. Biol. *21*, 441–446.

Bryngelson, J.D., Onuchic, J.N., Socci, N.D., and Wolynes, P.G. (1995). Funnels, pathways, and the energy landscape of protein folding: a synthesis. Proteins *21*, 167–195.

Cajal, S.R.y. (1903). Un sencillo metodo de coloracion seletiva del reticulo protoplasmatico y sus efectos en los diversos organos nerviosos de vertebrados e invertebrados. Trab. Lab. Invest. Biol. Univ. Madrid *2*, 129–221.

Cajal, S.R.y. (1910). El núcleo de las células piramidales del cerebro humano y de algunos mamíferos. Trab. Lab. Invest. Biol. Univ. Madrid *8*, 27–62.

Chen, C., Jin, J., James, D.A., Adams-Cioaba, M.A., Park, J.G., Guo, Y., Tenaglia, E., Xu, C., Gish, G., Min, J., and Pawson, T. (2009). Mouse Piwi interactome identifies binding mechanism of Tdrkh Tudor domain to arginine methylated Miwi. Proc. Natl. Acad. Sci. USA *106*, 20336–20341.

Chen, C., Nott, T.J., Jin, J., and Pawson, T. (2011). Deciphering arginine methylation: Tudor tells the tale. Nat. Rev. Mol. Cell Biol. *12*, 629–642.

de Thé, H., Chomienne, C., Lanotte, M., Degos, L., and Dejean, A. (1990). The t(15;17) translocation of acute promyelocytic leukaemia fuses the retinoic acid receptor alpha gene to a novel transcribed locus. Nature *347*, 558–561.

Decker, C.J., Teixeira, D., and Parker, R. (2007). Edc3p and a glutamine/asparagine-rich domain of Lsm4p function in processing body assembly in Saccharomyces cerevisiae. J. Cell Biol. *179*, 437–449.

Dedmon, M.M., Lindorff-Larsen, K., Christodoulou, J., Vendruscolo, M., and Dobson, C.M. (2005). Mapping long-range interactions in α-synuclein using spin-label NMR and ensemble molecular dynamics simulations. J. Am. Chem. Soc. *127*, 476–477.

Dellaire, G., Ching, R.W., Dehghani, H., Ren, Y., and Bazett-Jones, D.P. (2006a). The number of PML nuclear bodies increases in early S phase by a fission mechanism. J. Cell Sci. *119*, 1026–1033.

Dellaire, G., Eskiw, C.H., Dehghani, H., Ching, R.W., and Bazett-Jones, D.P. (2006b). Mitotic accumulations of PML protein contribute to the re-establishment of PML nuclear bodies in G1. J. Cell Sci. *119*, 1034–1042.

Dobson, C.M. (2003). Protein folding and misfolding. Nature *426*, 884–890.

Dundr, M., and Misteli, T. (2010). Biogenesis of nuclear bodies. Cold Spring Harb. Perspect. Biol. *2*, a000711.

Fanfoni, M., and Tomellini, M. (1998). The Johnson-Mehl-Avrami-Kohnogorov model: A brief review. Il Nuovo Cimento D *20*, 1171–1182.

Fitzpatrick, A.W., Debelouchina, G.T., Bayro, M.J., Clare, D.K., Caporini, M.A., Bajaj, V.S., Jaroniec, C.P., Wang, L., Ladizhansky, V., Müller, S.A., et al. (2013). Atomic structure and hierarchical assembly of a cross-β amyloid fibril. Proc. Natl. Acad. Sci. USA *110*, 5468–5473.

Flory, P.J. (1942). Thermodynamics of high polymer solutions. J. Chem. Phys. *10*, 51.

Forman-Kay, J.D., and Mittag, T. (2013). From sequence and forces to structure, function, and evolution of intrinsically disordered proteins. Structure *21*, 1492–1499.

Frey, S., Richter, R.P., and Görlich, D. (2006). FG-rich repeats of nuclear pore proteins form a three-dimensional meshwork with hydrogel-like properties. Science *314*, 815–817.

Gallivan, J.P., and Dougherty, D.A. (1999). Cation-π interactions in structural biology. Proc. Natl. Acad. Sci. USA *96*, 9459–9464.

Gromiha, M.M., Santhosh, C., and Ahmad, S. (2004). Structural analysis of cation-π interactions in DNA binding proteins. Int. J. Biol. Macromol. *34*, 203–211.

Hearst, S.M., Gilder, A.S., Negi, S.S., Davis, M.D., George, E.M., Whittom, A.A., Toyota, C.G., Husedzinovic, A., Gruss, O.J., and Hebert, M.D. (2009). Cajal-body formation correlates with differential coilin phosphorylation in primary and transformed cell lines. J. Cell Sci. *122*, 1872–1881.

Huggins, M.L. (1942). Some Properties of Solutions of Long-chain Compounds. J. Phys. Chem. *46*, 151–158.

Hyman, A.A., and Brangwynne, C.P. (2011). Beyond stereospecificity: liquids and mesoscale organization of cytoplasm. Dev. Cell *21*, 14–16.

Kato, M., Han, T.W., Xie, S., Shi, K., Du, X., Wu, L.C., Mirzaei, H., Goldsmith, E.J., Longgood, J., Pei, J., et al. (2012). Cell-free formation of RNA granules: low complexity sequence domains form dynamic fibers within hydrogels. Cell *149*, 753–767.

Kim, H.J., Kim, N.C., Wang, Y.-D., Scarborough, E.A., Moore, J., Diaz, Z., MacLea, K.S., Freibaum, B., Li, S., Molliex, A., et al. (2013). Mutations in prion-like domains in hnRNPA2B1 and hnRNPA1 cause multisystem proteinopathy and ALS. Nature *495*, 467–473.

Kirino, Y., Vourekas, A., Kim, N., de Lima Alves, F., Rappsilber, J., Klein, P.S., Jongens, T.A., and Mourelatos, Z. (2010). Arginine methylation of vasa protein is conserved across phyla. J. Biol. Chem. *285*, 8148–8154.

Kotaja, N., and Sassone-Corsi, P. (2007). The chromatoid body: a germ-cell-specific RNA-processing centre. Nat. Rev. Mol. Cell Biol. *8*, 85–90.

Kotaja, N., Bhattacharyya, S.N., Jaskiewicz, L., Kimmins, S., Parvinen, M., Filipowicz, W., and Sassone-Corsi, P. (2006). The chromatoid body of male germ cells: similarity with processing bodies and presence of Dicer and microRNA pathway components. Proc. Natl. Acad. Sci. USA *103*, 2647–2652.

Li, P., Banjade, S., Cheng, H.C., Kim, S., Chen, B., Guo, L., Llaguno, M., Hollingsworth, J.V., King, D.S., Banani, S.F., et al. (2012). Phase transitions in the assembly of multivalent signalling proteins. Nature *483*, 336–340.

Liang, L., Diehl-Jones, W., and Lasko, P. (1994). Localization of vasa protein to the Drosophila pole plasm is independent of its RNA-binding and helicase activities. Development *120*, 1201–1211.

Liu, K., Chen, C., Guo, Y., Lam, R., Bian, C., Xu, C., Zhao, D.Y., Jin, J., MacKenzie, F., Pawson, T., and Min, J. (2010). Structural basis for recognition of arginine methylated Piwi proteins by the extended Tudor domain. Proc. Natl. Acad. Sci. USA *107*, 18398–18403.

Marsh, J.A., and Forman-Kay, J.D. (2010). Sequence determinants of compaction in intrinsically disordered proteins. Biophys. J. *98*, 2383–2390.

Meikar, O., Da Ros, M., Korhonen, H., and Kotaja, N. (2011). Chromatoid body and small RNAs in male germ cells. Reproduction *142*, 195–209.

Melnick, A., and Licht, J.D. (1999). Deconstructing a disease: RARalpha, its fusion partners, and their roles in the pathogenesis of acute promyelocytic leukemia. Blood *93*, 3167–3215.

Misteli, T., Cáceres, J.F., Clement, J.Q., Krainer, A.R., Wilkinson, M.F., and Spector, D.L. (1998). Serine phosphorylation of SR proteins is required for their recruitment to sites of transcription in vivo. J. Cell Biol. *143*, 297–307.

Monsellier, E., and Chiti, F. (2007). Prevention of amyloid-like aggregation as a driving force of protein evolution. EMBO Rep. *8*, 737–742.

Montgomery, T.S. (1898). Comparative cytological studies, with especial regard to the morphology of the nucleolus. J. Morphol. *15*, 265–582.

Morozova, N., Allers, J., Myers, J., and Shamoo, Y. (2006). Protein-RNA interactions: exploring binding patterns with a three-dimensional superposition analysis of high resolution structures. Bioinformatics *22*, 2746–2752.

Phair, R.D., and Misteli, T. (2000). High mobility of proteins in the mammalian cell nucleus. Nature *404*, 604–609.

Shammas, S.L., Knowles, T.P., Baldwin, A.J., Macphee, C.E., Welland, M.E., Dobson, C.M., and Devlin, G.L. (2011). Perturbation of the stability of amyloid fibrils through alteration of electrostatic interactions. Biophys. J. *100*, 2783–2791.

Sun, Z., Diaz, Z., Fang, X., Hart, M.P., Chesi, A., Shorter, J., and Gitler, A.D. (2011). Molecular determinants and genetic modifiers of aggregation and toxicity for the ALS disease protein FUS/TLS. PLoS Biol. *9*, e1000614.

Tang, J., Frankel, A., Cook, R.J., Kim, S., Paik, W.K., Williams, K.R., Clarke, S., and Herschman, H.R. (2000). PRMT1 is the predominant type I protein arginine methyltransferase in mammalian cells. J. Biol. Chem. *275*, 7723–7730.

Tycko, R. (2011). Solid-state NMR studies of amyloid fibril structure. Annu. Rev. Phys. Chem. *62*, 279–299.

Wilkins, D.K., Grimshaw, S.B., Receveur, V., Dobson, C.M., Jones, J.A., and Smith, L.J. (1999). Hydrodynamic radii of native and denatured proteins measured by pulse field gradient NMR techniques. Biochemistry *38*, 16424–16431.

Wilson, E.B. (1899). The structure of protoplasm. Science *10*, 33–45.

Wooderchak, W.L., Zang, T., Zhou, Z.S., Acuña, M., Tahara, S.M., and Hevel, J.M. (2008). Substrate profiling of PRMT1 reveals amino acid sequences that extend beyond the “RGG” paradigm. Biochemistry *47*, 9456–9466.

Yaffe, M.B., Leparc, G.G., Lai, J., Obata, T., Volinia, S., and Cantley, L.C. (2001). A motif-based profile scanning approach for genome-wide prediction of signaling pathways. Nat. Biotechnol. *19*, 348–353.

Yamaguchi, A., and Kitajo, K. (2012). The effect of PRMT1-mediated arginine methylation on the subcellular localization, stress granules, and detergent-insoluble aggregates of FUS/TLS. PLoS ONE *7*, e49267.

Yeo, G.C., Keeley, F.W., and Weiss, A.S. (2011). Coacervation of tropoelastin. Adv. Colloid Interface Sci. *167*, 94–103.

Molecular Cell, Volume *57*

Supplemental Information

Phase Transition of a Disordered Nuage Protein Generates Environmentally Responsive

Membraneless Organelles

Timothy J. Nott, Evangelia Petsalaki, Patrick Farber, Dylan Jervis, Eden Fussner, Anne Plochowietz, Timothy D. Craggs, David P. Bazett-Jones, Tony Pawson, Julie D. Forman-Kay, and Andrew J. Baldwin

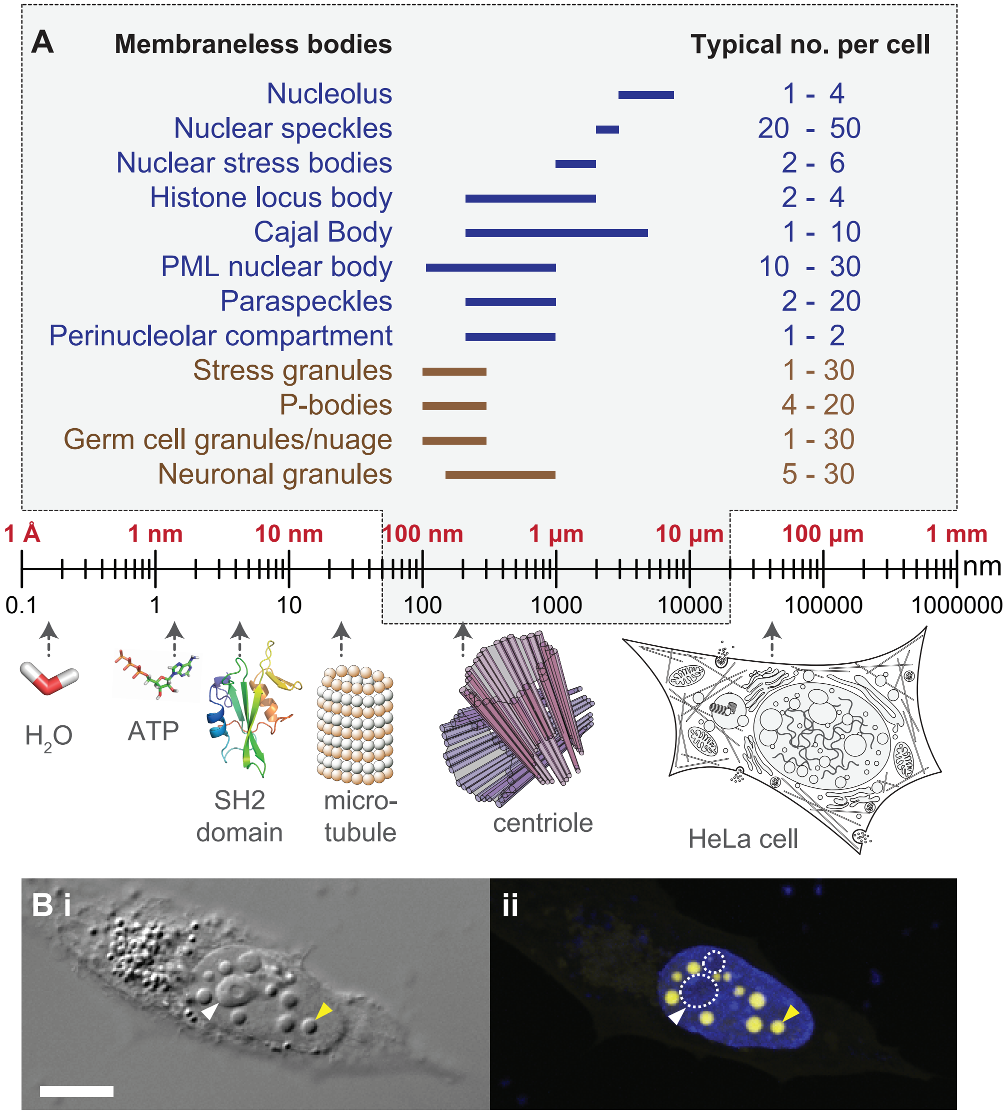

Figure S1 related to Figure 1: A Table and schematic showing the relation between the relative sizes and number of membraneless nuclear organelles commonly observed within cells compared to the sizes of other sub-cellular components. Table (gray) adapted from (Dundr and Misteli, 2010). **B.** Differential interference contrast (DIC, i) and corresponding extended focus fluorescence intensity (ii) images of a HeLa cell expressing Ddx4^{YFP}. Ddx4^{YFP} forms dense, spherical organelles (yellow arrow head) in the nucleus. The nucleolus is highlighted with white arrowheads and dashed white line (ii). Hoechst stain (blue) was used to stain chromatin within the nucleus. Scale bar 10 μm.

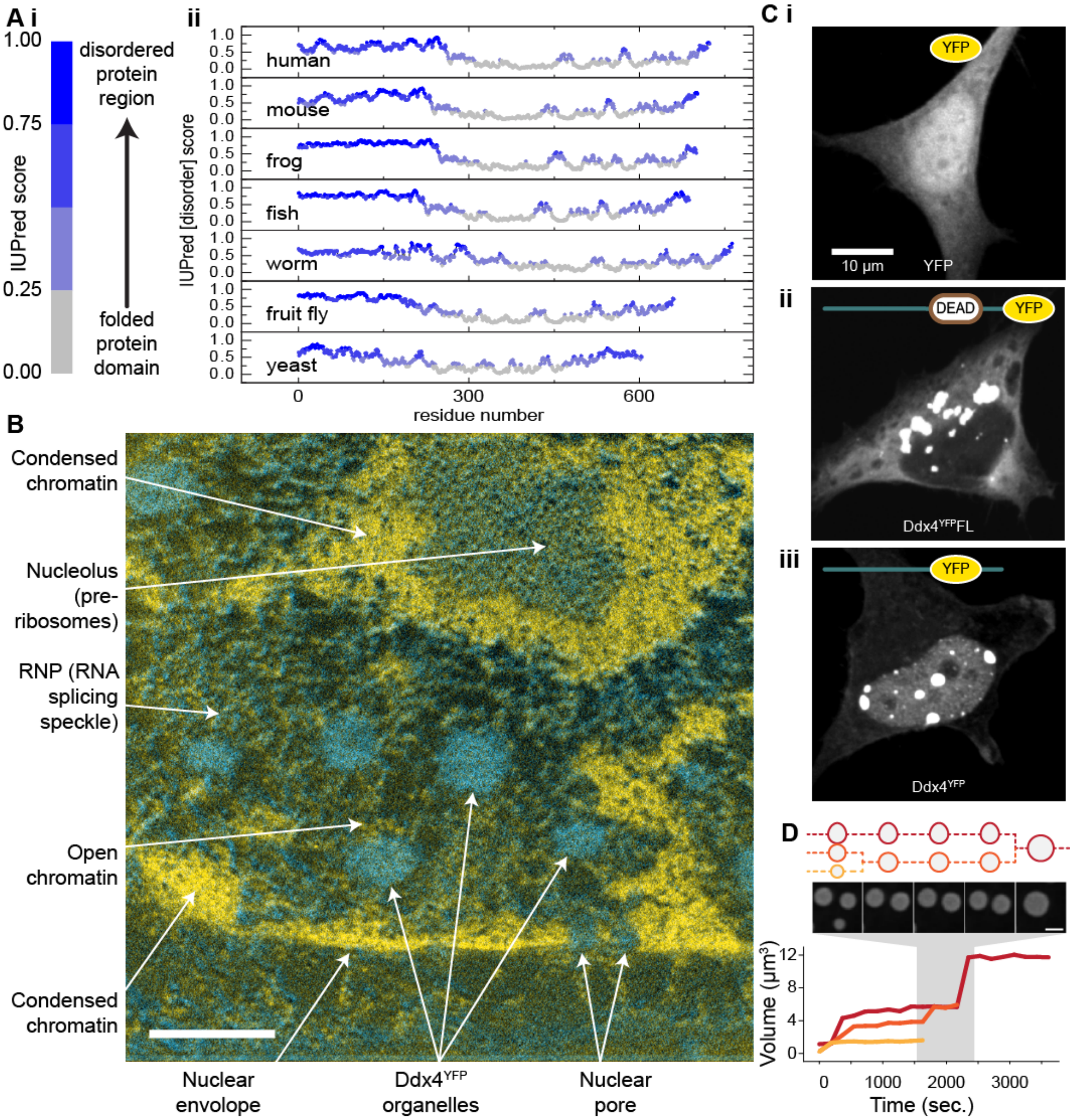

Figure S2 related to Figure 2 – Properties of isolated Ddx4 domains. A. IUPred predictions for Ddx4 orthologs. **B.** Phosphorus (yellow) and nitrogen (cyan) ESI images of HeLa cells containing Ddx4^{YFP} organelles. Scale bar 0.5 μm. **C.** Extended focus images of fixed HeLa cells expressing YFP alone (i), Ddx4^{YFP}FL (ii), and Ddx4^{YFP} (iii). **D.** Coalescence of Ddx4^{YFP} organelles within the nucleus of a HeLa cell. Scale bar 2 μm.

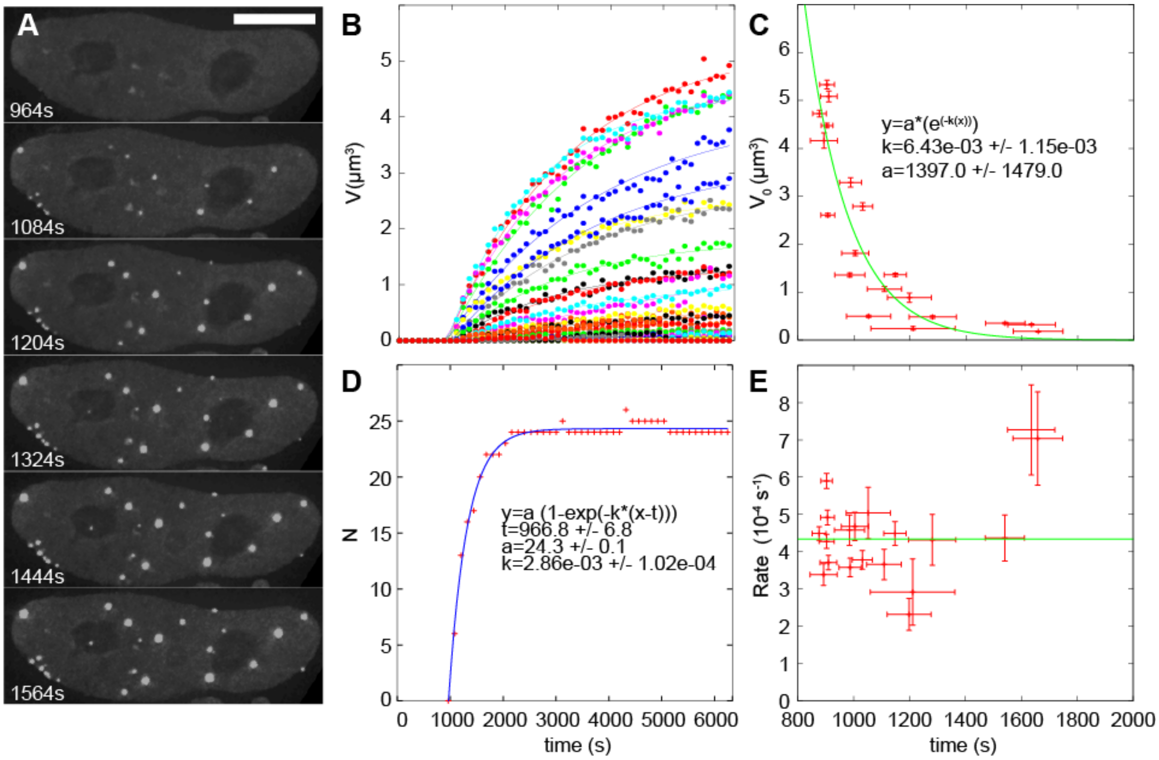

Figure S3 related to Figure 3: Kinetic analysis of growth of Ddx4^{YFP} organelles within the HeLa nucleus. A. Extended focus fluorescence image series of the initial growth of Ddx4^{YFP} organelles from a pool of dispersed material with a HeLa cell nucleus. Scale bar 10 μm. **B.** The volume of individual droplets of Ddx4^{YFP} within a single HeLa nucleus was followed with time. The growth curves were well described by an Avrami model of nucleated growth (Equation S6) where each droplet has an independent growth rate, *k*, steady-state volume, V_T and nucleation time *t'*. **C.** The variation in the steady-state volume of individual droplets varied with the time that they first appeared and decreased with time according to $\boldsymbol{V_T(t) = V_T^0 e^{-k_F t}}$ where k_F = 6.4 ± 1.2 x 10^{-3} s^{-1} . The extrapolated value for V_T^0 was 1397 μm^3. Error bars come from the standard error in the curve fitting (B). **D.** The number of unique droplets was found to increase such that $\boldsymbol{N(t) = N_0\left(1 - e^{-k_N(t-t_0)}\right)}$ where N_0 = 24.3 ± 0.1, k_N = 29 ± 1 x 10^{-4} s^{-1} and t_0 is the time for the appearance of the first droplet, 967 ± 7 s. **E.** The growth rates were found to cluster around a central value, though variation was observed between individual droplets such that the average and standard deviation rates were 4.5 and 1.2 x 10^{-4} s^{-1}. Error bars come from the standard error in the curve fitting (B).

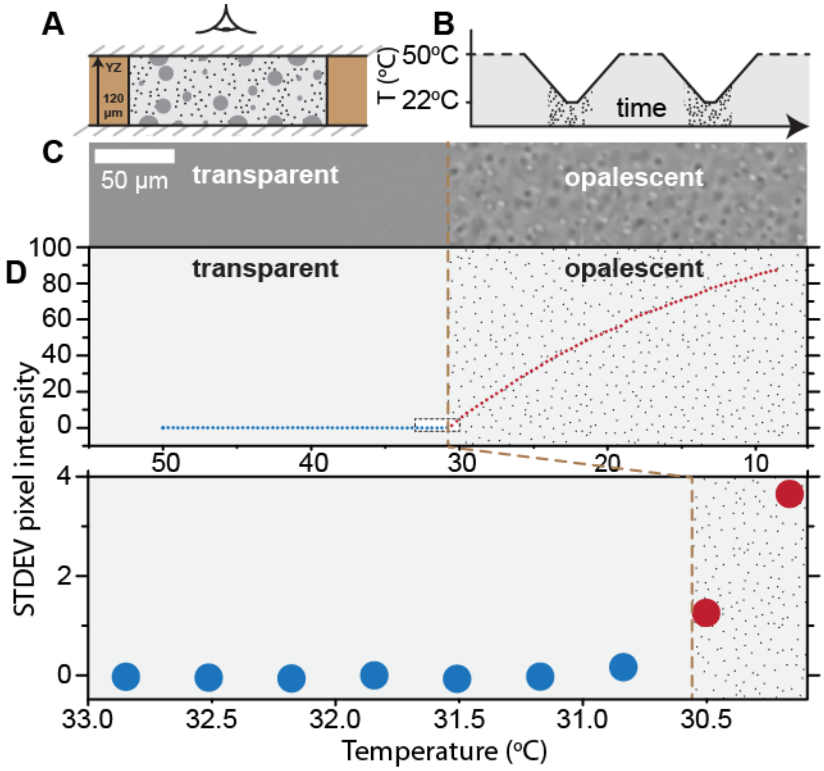

Figure S4 related to Figure 4– Estimation of the binodal phase transition temperature T_p. A. A schematic showing the arrangement of the sealed sample chamber on top of the THMS600 thermal stage with respected to the observer. Top and bottom coverslips (gray dashes) and SecureSeal imaging spacers (brown) were assembled to form a sealed sample chamber containing a protein solution **B.** Heating cycles showing that phase separation was induced by cooling, and reversed upon re-heating. **C.** Bright field images (XY-plane) of $Ddx4^{N1}$ above and below the transition temperature. **D.** Example of the determination of the $Ddx4^{N1}$ transition point by the change in standard deviation in pixel intensity with temperature (i) and close up showing that the transition was determined to be 30.5°C.

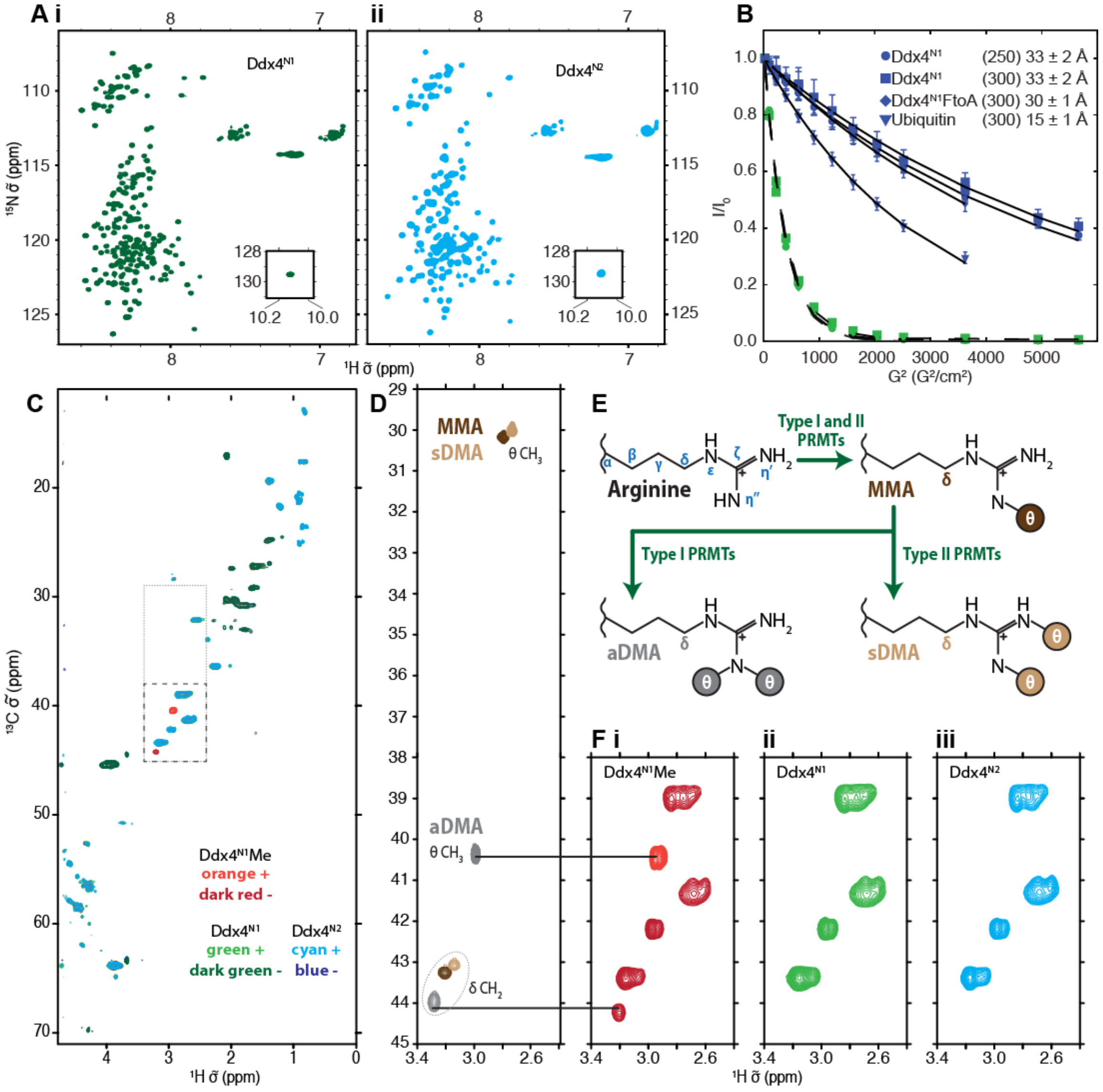

Figure S5 related to Figure 5 – NMR spectroscopy analysis of Ddx4^{N1}, Ddx4^{N2} and Ddx4^{N1}Me.

A. ^{15}N-^{1}H HSQC spectra of Ddx4^{N1} (i) and Ddx4^{N2} (ii). From the dispersion of chemical shifts both proteins are intrinsically disordered. **B.** The loss of intensity as a function of gradient field strength in a PFGSE experiments from various Ddx4 and Ubiquitin constructs with NaCl concentrations in mM in brackets and R_H values. The error bars indicate the standard deviation of the normalized intensity from individual peaks at each gradient strength. **C.** ^{13}C-^{1}H HSQC spectra of Ddx4^{N1} (greens), Ddx4^{N2} (blues) and Ddx4^{N1}Me (reds). Positive contours indicate CH_3/CH group and negative contours indicate CH_2 groups. The peak positions and intensities are identical, apart from the appearance of an additional two resonances in the box indicated upon methylation. **D.** NMR spectra of symmetric dimethylated arginine (sDMA, light brown), asymmetric dimethylated arginine (aDMA, grey) and mono-methylated arginine (MMA, dark brown). The three different chemical modifications are

clearly distinguished by NMR spectroscopy. **E.** The locations of the three different modifications. **F.** Comparison of the spectra of Ddx4^{N1}Me (red), Ddx4^{N1} (green) and Ddx4^{N2} (blue). The two additional resonances observed in the methylated form are consistent only with the aDMA form, and were assigned using recently published values for θ CH_3 and δ CH_2 groups from short peptides (Theillet et al., 2012). The observation that the resonances from the $^{\theta}CH_3$ groups of up to six aDMA residues are completely overlapped indicates that all such methyl groups experience relatively similar chemical environments, consistent with the disordered nature of the N-terminus of Ddx4.

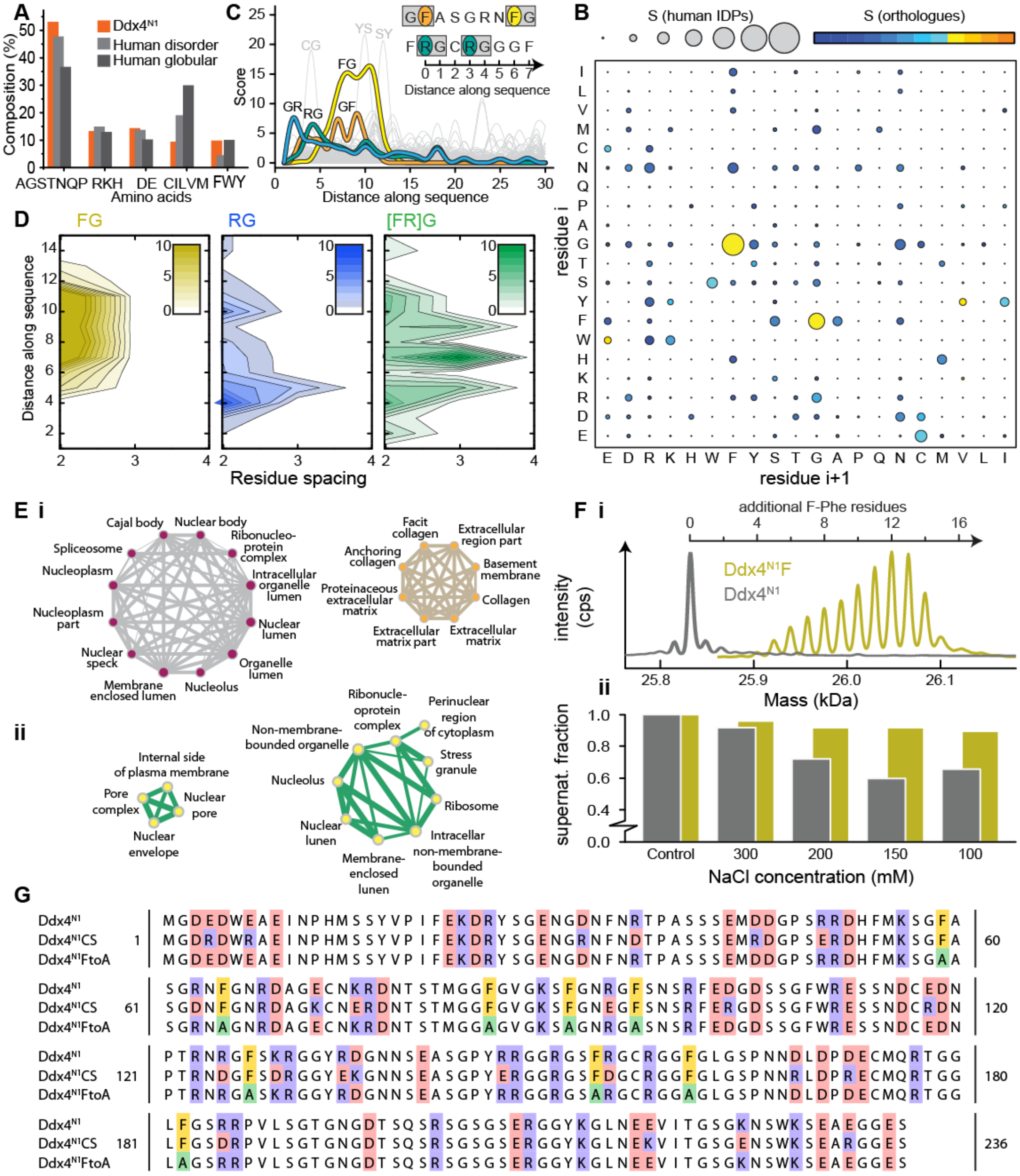

Figure S6 related to Figure 6: Statistical analysis of the sequence-based features that enable Ddx4^{N1} to form droplets.

A. Residue types in Ddx4^{N1} compared to globular and disordered portions of the human proteome. **B.** Frequency and statistical significance of pairs of amino acids in the disordered regions of human Ddx4 and its orthologs over a background signal from the proteomes of these species. 46 organisms were considered, from which 68 Ddx4 orthologs were identified. The size of the dots indicates the over-representation of a given pair in Ddx4 orthologs over those in the remaining intrinsically disordered parts of the proteomes of the 46 organisms. Colour represents the conservation of a given

amino acid pairing in the disordered regions of the Ddx4 ortholog family where warmer colours indicate a higher significance. The GF and FG pairing is both overrepresented when compared to the background proteome (larger size) and is highly conserved within the Ddx4 family (warmer colour). **C.** The separation between pairs of dipetides within Ddx4 disordered regions, normalised by their background frequency. Significant trends were observed for both FG/FG and the RG/GR spacings, suggesting the presence of strong evolutionary pressure to maintain these distances within the disordered regions of Ddx4 proteins. **D.** Similarity maps showing the spacings of F-F, R-R and F-R (each followed or preceded by a G) residues, extended to 3 and 4 repeats. **E.** The gene ontology (GO) terms for the top 10% of identified sequences were analysed and represented here as a network map from the human (i) and yeast (ii) genomes. The terms that occur are shown in text, where the size of the node indicates how likely the term was to appear in the dataset, over the background. The weight of the lines indicates the number of times that a sequence contains both GO terms. **F.** Fluorination of phenylalanine residues destabilises Ddx4 organelles. Incorporation of DL-3-flourophenylalanine (F-Phe) into $Ddx4^{N1}$ ($Ddx4^{N1}F$), measured by mass spectrometry (i), and its effect on phase-separation as monitored by remaining soluble protein following centrifugation (ii). **G.** Alignment of the amino acid sequences of $Ddx4^{N1}$, $Ddx4^{N1}CS$ and $Ddx4^{N1}FtoA$. Residues involved in mutational strategies are highlighted.

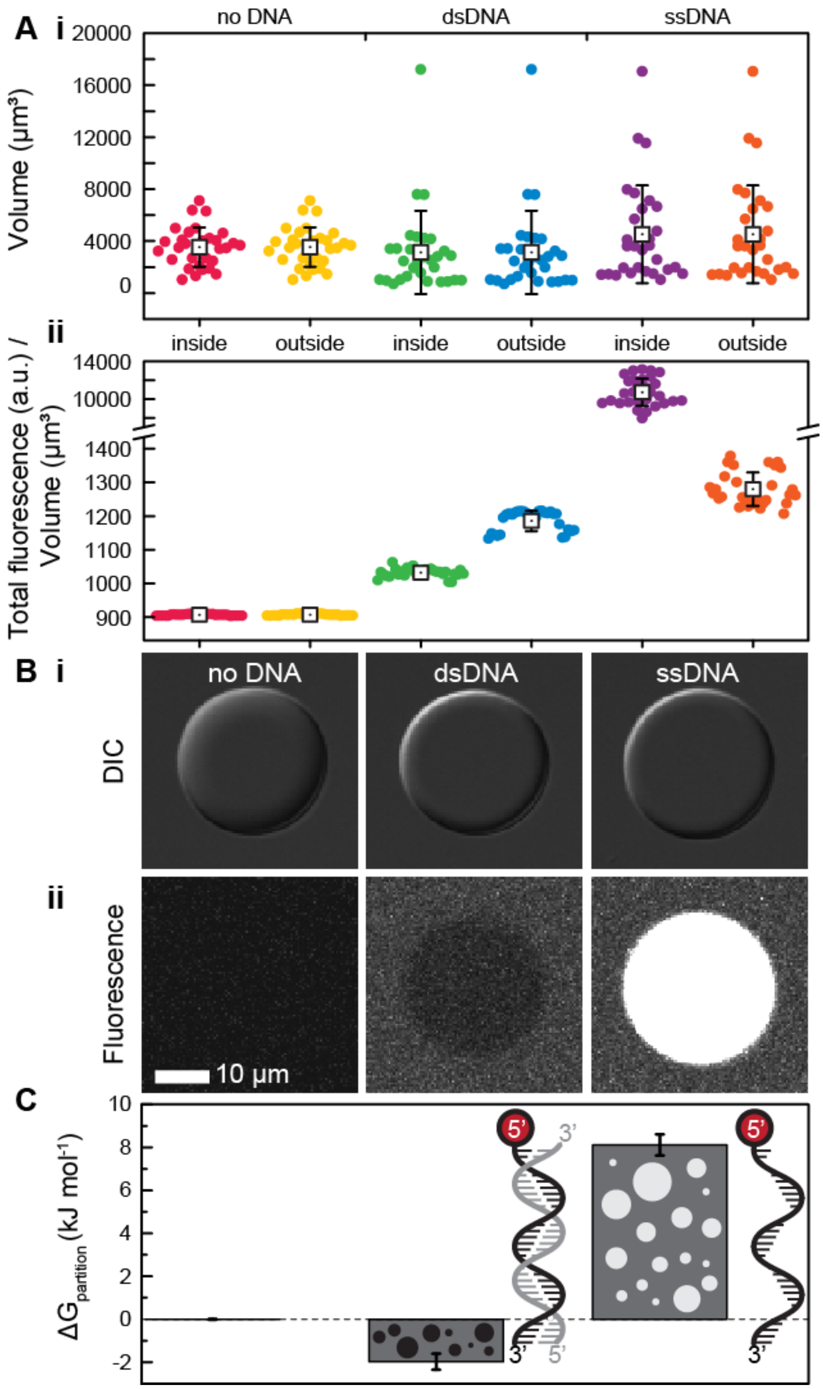

Figure S7 related to Figure 7: Ddx4^{N1} organelles both in isolation, and with double stranded and single stranded DNA of 32 bases in length. **A i).** Volumes of organelles and surrounding aqueous phase used in analysis. **ii)** Volumes normalised by total fluorescence to account for differences in ROI size. Error bars indicate one standard deviation. **B i)** DIC and **ii)** fluorescence images of a single representative organelle, acquired and processed identically. The fluorescence signal in the nucleic acid free sample was used as a baseline and subtracted from those containing ssDNA or dsDNA. **C.**

The partition free energy of the difference substances. The error bar of the empty organelle indicates the experimental uncertainty inherent to the experimental setup.

Supplementary movies:

Movie M1 related to Figure 1. Nucleation and growth of Ddx4^{YFP} organelles. Scale bar 10 µm. Time in seconds (top left of screen). Organelles can be seen to spontaneously appear inside a HeLa cell nucleus.

Movie M2 related to Figure 2. Cold shock inducing rapid formation of Ddx4^{YFP} organelles. Scale bar 10 µm. Time in minutes (top left of screen).

Movies M3 and M4 related to Figure 2. Osmotic shock causing rapid dissolution and condensation of Ddx4^{YFP} organelles. Scale bar 10 µm. Time in minutes (top left of screen).

Supplementary Experimental Procedures

S1) Protein expression and purification

Genes for Ddx4 mutants (Ddx4^{YFP}, Ddx4^{N1}, Ddx4^{N2}, Ddx4^{N1}FtoA and Ddx4^{N1}CS) were synthesized by GenScript and sub-cloned. The recombinant Ddx4 proteins were expressed from IPTG-inducible plasmids (unless otherwise stated a modified pETM-30 vector containing the pGEX-2T-TEV site and pProEx multiple cloning site) in *E. coli* BL21(DE3) cells overnight at 20°C. Cell pellets were suspended in buffer (50 mM Tris pH 8.0, 500 mM NaCl, 5 mM DTT) and lysed by homogenization. Proteins were purified by affinity chromatography (GST-4b beads; GE Healthcare Life Sciences), the tag was removed with TEV protease, eluted and further purified and buffer-exchanged by size-exclusion chromatography into storage buffer (20 mM Tris pH 8.0, 300 mM NaCl, 5 mM TCEP). Purified proteins were centrifugally concentrated, typically to 300-500 μM, flash-frozen in liquid nitrogen and stored at -80°C.

Methylated Ddx4, Ddx4^{N1}Me, was produced by co-transforming competent *E. coli* BL21(DE3) cells with IPTG-inducible plasmids containing Ddx4^{N1} (residues 1-236; kanamycin resistance marker) and PRMT1 (ampicillin-resistance marker). Colonies containing both plasmids were selected on agar plates containing both kanamycin and ampicillin before expression and purification as previously described. As recombinant PRMT1 did not contain a TEV-cleavable site it remained bound to GST beads when the methylated Ddx4 protein was eluted.

Isotopically enriched Ddx4 proteins were grown in M9 minimal media containing $^{15}NH_4Cl$ as the major nitrogen source for ^{15}N labeling and D-[^{1}H-^{13}C] glucose as the major carbon source for ^{13}C labeling. Nitrogen and carbon isotopes were purchased from Cambridge Isotope Labs. Incorporation of DL-3-flourophenylalanine Ddx4 (Ddx4^{N1}F) was produced by growing Ddx4 proteins in M9 minimal media supplemented with 0.1% $^{14}NH_4Cl$ and 0.3% D-[^{1}H-^{12}C] glucose as the sole sources of nitrogen and carbon. Labeling with 3-fluorophenylalanine was achieved by allowing cell cultures at 37°C to reach an OD_{600} of 0.8, whereupon 1 g/L glyphosate, 75 mg/L L-tryptophan, and 75 mg/L L-tyrosine was added. Once cell cultures reached an OD_{600} of 1.0 (after approximately 1 h), 150 mg/L DL-3-fluorophenylalanine was added and expression was induced with the addition of IPTG at 20°C. Purification was performed as described above. The mass of purified proteins was confirmed by electrospray ionization mass spectrometry.

For functional assays DNA coupled to the fluorescent dye Atto647N at the 5' end through a dT-C6 linker (marked as **X**) was synthesized. The sequence of this oligo (referred to as *oligo) was **X**TTTTTCCTAGAGAGTAGAGCCTGCTTCGTGG. Unlabelled sense and antisense versions of *oligo were also synthesized. Single and double stranded DNA (ssDNA and dsDNA, respectively) was prepared by

mixing *oligo with its sense or antisense strand at 1:1 molar ratio. The *oligo mixtures were then heated at 95^oC for 3 min and cooled to 40^oC over 45 minutes to allow labelled and unlabelled oligos to anneal.

HeLa Cell culture

HeLa cells were cultured on 35 mm glass-bottomed MatTek dishes or 25 mm glass coverslips in growth media (high glucose DMEM containing 20 mM HEPES pH 7.4, 10% FBS and antibiotics at 37^oC and 5% CO_2). Ddx4 constructs (Ddx4^{YFP}, Ddx4^{YFP}FL, Ddx4^{YFP}FtoA and Ddx4^{YFP}CS) were expressed in HeLa cells from pcDNA 3.1+ (Invitrogen) plasmids by transient transfection utilizing the Effectene (Qiagen) or polyethylenimine (PEI) methods. Transfections were carried out according to the manufacturers instructions and used 0.5 – 1 μg plasmid DNA per MatTek dish or coverslip.

Live cell imaging

Live cell imaging experiments (Figure 2 A-C, S3 A-E and movies M1-M4) were performed on a Leica DMIRE2 inverted microscope equipped with a PZ-2000 XYZ series automated stage with Piezo Z-axis top plate (Applied Scientific Instrumentation) and Hamamatsu C10600-10B (ORCA-R2) camera. Cells under observation were live. Microscope hardware, image acquisition and analysis were controlled with Volocity software. Samples were observed with wide field illumination using a YFP filter set (excitation filter = 500 nm, emission filter = 535 nm) and a Leica HCX PL APO 40x oil immersion objective, numerical aperture (NA) 1.3.

Growth of Ddx4^{YFP} organelles was sampled every 2 minutes for a total of 54 time points. At every time point 41 Z-slices (0.4 μm step size) were captured, each with an exposure time of 13 ms at bin 1, and with a bit depth of 12 (gray values from 0 - 4095). For the duration of the experiment the cell under observation remained in the center of the Z-stack. After acquisition, out of focus light was reduced using a point spread function (PSF), calculated for the optics of the system. The PSF had a Z-spacing of 0.05 μm, lateral spacing in X-Y of 0.067 μm, medium refractive index of 1.52, NA of 1.3 and emission wavelength of 535 nm. Automated corrections for photo-bleaching, sample movement and contrast enhancement were performed using Volocity software. Ddx4^{YFP} organelles were identified as regions with >6 standard deviations in pixel intensity higher than the mean of the field of view (a rectangle encompassing the whole cell). This definition was used to determine the volume of Ddx4^{YFP} organelles from deconvolved image stacks. Individual Ddx4^{YFP} organelles were tracked from one frame to the next using the automated tracking function within Volocity.

Temperature and tonicity-responses of Ddx4^{YFP} organelles were measured in HeLa cells *in situ* on the microscope. To vary the temperature of the cell between the second and third time point, growth media was rapidly aspirated, and replaced with 2 ml growth media, pre-cooled on ice. Image capture,

deconvolution, and image thresholding for volumetric measurements was the same as described above except the exposure time during acquisition was 15 ms per Z-slice.

The effect of tonicity on Ddx4^{YFP} organelles was assayed using the osmotic shock method. Tonicity was changed by aspirating growth media (and setting it aside at 37°C) before replacing it with 2 ml of ddH_2O that had been pre-warmed to 37°C. Once Ddx4^{YFP} organelles had dissolved, the ddH_2O was aspirated and replaced with the original growth media (at 37°C). Stacks of 76 Z-slices (0.4 μm step size, 3 ms exposure time) were acquired every minute at bin 2. Image capture, deconvolution and image thresholding for volumetric measurements was performed as described previously.

In vitro **droplet preparation for microscopy**

Small volumes of Ddx4^{N1} and Ddx4^{YFP} dense liquid phase were prepared *in vitro* using the vapor-diffusion hanging drop method. This entailed a droplet containing purified protein and buffer equilibrating by vapor diffusion with a larger reservoir containing the buffer alone. Hanging droplets were supported on siliconized coverslips (22 mm diameter, 0.22 mm thickness) from Hampton Research, sealed to the well with Vaseline. To initiate rapid phase separation, 1.5 μl of Ddx4^{N1} or Ddx4^{N1}Me was deposited on a coverslip, diluted 1:1 with 0 mM NaCl buffer (20 mM Tris pH 8.0, 5 mM TCEP), and equilibrated over a 150 mM NaCl reservoir solution. Once equilibrated (>30 min at 22°C), coverslips were taken off the well, and excess Vaseline removed with a 200 μl pipette tip. The droplet was then gently dispersed onto a microscope slide, and sealed in place with the remaining Vaseline.

S2) *In vitro* and live cell imaging

Confocal fluorescence microscopy

Confocal fluorescence microscopy was used to observe Ddx4^{YFP} in living and fixed HeLa cells and for measurement of the diffusion properties of purified Ddx4 protein *in vitro*. These experiments were performed using an Olympus IX81 inverted microscope equipped with 60x (NA 1.3) and 30x (NA 1.05) silicon immersion objectives. Microscope hardware and laser optics were controlled by FLUOVIEW FV1000 software.

Imaging fixed HeLa cells

HeLa cells expressing YFP, Ddx4^{YFP}, Ddx4^{YFP}FL, Ddx4^{YFP}FtoA and Ddx4^{YFP}CS were grown on MatTek dishes or 25 mm glass coverslips, and fixed with 4% paraformaldehyde (PFA) in phosphate buffered saline (PBS), for 5 minutes at 37°C. Cells were then washed three times with PBS to remove excess PFA. Next, cells were permeabilised with 0.5% TritonX-100 (in PBS) for 10 minutes, and again washed three times with PBS. Nuclei were visualized with Hoechst or DAPI stain. Cells were washed a further two times with PBS to remove excess Hoechst/DAPI stain and imaged using an Olympus

IX81 inverted microscope with a 60x (NA 1.3) silicon immersion objective. Hoechst/DAPI dye was excited with a 405 nm laser and YFP was excited with a 515 nm laser. Hoechst/DAPI and YFP fluorescence were detected at 461 and 527 nm respectively. Differential interference contrast (DIC) images were collected using illumination from the 405 nm laser.

For immunofluorescence experiments, HeLa cells expressing Ddx4^{YFP} were grown on 25 mm diameter #1.5 glass coverslips (Warner Instruments). Fixation and permeabilisation of samples was performed as above. Cells were then blocked with goat serum (5% in PBS) for one hour at room temperature before antibody staining. Primary antibodies were diluted to between 1:10 and 1:100 (in PBS containing 5% goat serum) before use. Nucleoli and PML bodies were simultaneously labelled using mouse B23 (Santa Cruz sc-56622) and rabbit PML (Abcam ab-72137) antibodies, respectively. Nuclear speckles and Cajal bodies were simultaneously labelled using mouse SC-35 hybridoma (ATCC 1023768), and rabbit coilin (Santa Cruz sc-32860) antibodies, respectively. Following incubation at 4°C overnight, excess primary antibodies were removed by washing the cells three times with PBS (five minutes per wash).

Cells were then incubated with Cy3 goat anti-mouse, and Cy5 goat anti-rabbit, secondary antibodies (diluted 1:400 and 1:200 in PBS, respectively) for 1 hour at room temperature. Excess secondary antibodies were removed by washing with PBS, as above. Nuclei were visualized with DAPI stain. Coverslips were then mounted on microscope slides using glycerol/*n*-propyl gallate mounting medium, sealed with nail varnish, and imaged using an Olympus IX81 inverted microscope equipped with a 60x (NA 1.3) silicon immersion objective. DAPI, YFP, Cy3 and Cy5 dyes were excited with 405 nm, 515 nm, 559 nm and 635 nm lasers, respectively. 26 Z-slices (0.4 μm spacing, 12.5 μs pixel^{-1} scanning speed, 12 bit depth) were captured for each channel.

Fluorescence-recovery after photo-bleaching (FRAP)

FRAP experiments on Ddx4^{YFP} organelles in live HeLa cells were performed at 37°C and 5% CO_2 in a live cell chamber (Precision Plastics Ltd) mounted on an Olympus IX81 inverted microscope with a 60x (NA 1.3) silicon immersion objective. Photo-bleaching of a 1.5 μm Ddx4^{YFP} organelle was achieved using a 515 nm laser with a bleaching time of 1 second. Fluorescence emission was monitored at 527 nm. Images (150 x 112 pixels, 12 bit depth) were captured at 218 ms intervals with a scanning speed of 2 μs pixel^{-1} for 100 time points.

In vitro FRAP experiments were performed on samples of the dense phase of Ddx4, prepared using the hanging drop method. hDdx4^{N1}, free CFP and hDdx4^{YFP} (all in storage buffer) were first mixed to produce a sample containing the three proteins at individual concentrations of 292.5, 8.1 and 8.1 μM respectively. This mixture was then diluted 1:1 with 0 mM NaCl buffer (20 mM Tris pH 8 at RT; 5 mM

TCEP) and equilibrated as a hanging drop over a well containing 150mM NaCl buffer for 48 hours. 30 minutes prior to the FRAP experiment the cover slip supporting the hanging droplet was removed from the well, excess Vaseline removed with a yellow (200 µL) pipette tip, and the cover slip placed on a microscope slide such that the hanging droplet was dispersed across its surface and sealed in place with the remaining Vaseline. *In vitro* FRAP was performed on a 10 µm diameter droplet of dense-phase protein using a 515 nm laser (YFP channel) and a 440 nm laser (CFP channel) to simultaneously bleach both fluorophores. Laser power (transmissivity) for the bleach period was 100% for 0.2 seconds and 1% for acquisition. Fluorescence emission was monitored at 527 nm (YFP channel) and 476 nm (CFP channel). Images (512 x 512 pixels, 12 bit depth) were captured at both emission wavelengths with a scanning speed of 2 µs pixel^{-1} at 3 second intervals for a total of 360 time points. The data were analysed using to Fick's law of Diffusion. In one dimension, the number of particles at a distance x from the source at time t will be given by

$$n(x,t) = n_0 Erfc\left(\frac{x}{2\sqrt{Dt}}\right) \quad [S1]$$

where Erfc is the complimentary error function. If we bleach a droplet with a known beam size, the time required for a particle at the center of the droplet to reach half that of the outside, then the diffusion coefficient *D* is given by:

$$\frac{1}{t_{1/2}}\left(\frac{x}{2Erfc^{-1}1/2}\right)^2 = D \quad [S2]$$

where Erfc^{-1}(0.5)~0.4769 and x is the radius of the beam. Recovery of the fluorescence of free protein, outside of organelles both in cells and *in vitro* was too fast for our measurements. We estimated a decay rate based on 99% recovery within the measurement time. The diffusion measurements we obtained in this way were comparable to the diffusion rates of freely diffusing protein measured using NMR (vide infra, figure S5). Diffusion of protein in organelle droplets both *in vivo* and *in vitro* were two orders of magnitude lower than the value obtained both for freely diffusing protein.

Electron Spectroscopic Imaging (ESI) microscopy

Samples were fixed and processed for EM as previously described (Ahmed et al., 2008). Briefly, Ddx4^{YFP} structures were identified using correlative LM/ESI by indirect immunofluorescence labeling of YFP after paraformaldehyde fixation and triton permeabilization. Samples were post-fixed with glutaraldehyde, dehydrated in an ethanol series, and embedded in Quetol 651 resin before ultra-thin sectioning with a Leica microtome. Samples were placed on copper finder grids to identify structures of interest and carbon coated with a 3-5 nm carbon film to improve sample stability. Nitrogen ratio maps were collected at 200 kV on a Tecnai transmission electron microscope of Ddx4

nuclear foci at energy offsets of 383 and 416 eV using a GATAN energy filter. To analyze the relationship between the $Ddx4^{YFP}$ nuclear foci and neighbouring structures, such as chromatin, we collected phosphorus ratio maps at energy offsets of 120 and 155 eV. Images were processed with ImageJ and Photoshop to demonstrate the relationship between chromatin, nuclear bodies, and $Ddx4^{YFP}$ structures. Protein-based structures are pseudo-coloured blue, and chromatin structure are pseudo-coloured in yellow. Nitrogen levels were normalised to zero in chromatin-rich regions, so that only the protein-dense domains that are not chromatin-associated are visualized. Data analyses were preformed on non-normalised, unfiltered images.

S3) NMR Spectroscopy

All experiments were performed on Varian INOVA spectrometers (11.7 and 18.8 T) equipped with room-temperature triple resonance probes. NMR spectra were processed using the NMRpipe suite of programs (Delaglio et al., 1995) and visualized in Sparky (Goddard and Kneller, 2004). ^{15}N-^{1}H sensitivity enhanced HSQC spectra of 150 μM $Ddx4^{N1}$ were recorded at 20°C at a field strength of 18.8 T in 20 mM sodium phosphate, 400 mM NaCl, 5mM DTT, and 10% $D_2 0$ at pH 6.0. A typical experiment employed acquisition times of (40, 64) ms (t1, t2) with a pre-scan delay of 1s, 100 increments and 16 scans per increment giving a total experiment time of 1 hour. ^{13}C-^{1}H constant time HSQC spectra of 200 uM ^{13}C enriched $Ddx4^{N1}$, $Ddx4^{N2}$ and $Ddx4^{N1}Me$ were recorded at 25°C at a field strength of 18.8 T in 20mM Tris, 300 mM NaCl, 0.5 mM TCEP and 10% D_2O at pH 8. A typical experiment employed acquisition times of (30, 64) ms (t1, t2) with a pre-scan delay of 1.5s, 218 increments with 8 scans per increment giving a total experiment time of 1 hour 34 minutes. ^{13}C-^{1}H natural abundance constant time HSQC spectra of 10mM methyl arginine standards, aDMA, sDMA and MMA were recorded in 10% D_2O at 25°C. Typically, acquisition times of (30, 64) ms (t1, t2) with a pre-scan delay of 1s, with 128 increments and 8 scans per transient giving a total experiment time of 38 minutes.

Pulsed field gradient (PFG) diffusion experiments were performed at 37°C at 11.7 T on 130 μM samples of $Ddx4^{N1}$ and $Ddx4^{N1}FtoA$ in 10mM Tris, 250mM NaCl, 5mM TCEP, 0.04% dioxane and 10% $D_2 0$. A 1D ^{1}H pulse gradient stimulated echo longitudinal encode-decode (PG-SLED) experiments with a watergate solvent suppression was employed (Wilkins et al., 1999), with 512 transients averaged per 1D, and 12 linearly spaced gradient strengths with a total experimental time of 6 hours. Experiments were carried out with dioxane used as an internal standard with an effective hydrodynamic radius ($R_{h,ref}$) of 2.12 Å. Decay profiles were analyzed using an in-house written MATLAB program, where the signal I is expected to decay according to $I = Ae^{-dG^2}$ where G is the gradient strength and d and A are constants. The hydrodynamic radius of the protein $R_{h,prot}$ can be obtained from:

$$R_{h,prot} = \frac{d_{ref}}{d_{prot}}\left(R_{h,ref}\right)$$

[S3]

Where d_{ref} and d_{prot} are the decay rates for dioxane and the protein, respectively. Each chemical shift was individually interrogated and analysed to give a value for d_{prot} providing four or more gradient points had signal that was more than twice the height of the maximum noise. The decay rates were then averaged, and the standard deviation used to provide an uncertainty estimate.

S4) Mass Spectrometry

Electrospray ionization mass spectrometry (ESI-MS)

Electrospray ionization mass spectrometry (ESI-MS) analyses were conducted using a QStar XL quadrupole time-of-flight mass spectrometer (AB Sciex, Concord, ON) equipped with an Ionspray source and a modified HSID interface (Ionics, Bolton, ON). Online desalting of protein samples was accomplished using a size exclusion medium (Sephadex, GE Healthcare Biosciences, Piscataway USA) at a flow rate of 250 µL min^{-1}. The mobile phase was composed of one-to-one mixture of methanol/aqueous 0.1% formic acid. Protein mass spectra were processed using the Bayesian Protein Reconstruction algorithm implemented in the BioAnalyst 1.1.5 software package (AB Sciex, Concord, ON). The AIMS Mass Spectrometry Laboratory in the Department of Chemistry at the University of Toronto performed ESI-MS.

Fragmentation mapping of Ddx4^{N1}Me methylation sites

For reduction and alkylation, 40 µL of 10 mM DTT in 100 mM NH_4HCO_3 was added to the Ddx4^{N1}Me sample solution and incubated for 1 hour at 56°C before being cooled to room temperature. 20 µL of 55 mM iodoacetamine in 100 mM NH_4HCO_3 was subsequently added before incubated at room temperature for 45 minutes in the dark. Trypsin digestion was performed in 2 mL of 50 mM NH_4HCO_3 with 25 µg sequencing grade trypsin (Roche Diagnostic Cat# 11418475001) at a final concentration of 12.5 ng µL^{-1}. Digestion buffer was then added to the sample solution to reach protein to enzyme ratio of 100:1 and incubated overnight at 37°C. For digestion with endoproteinase GluC, protein samples were lyophilized after reduction and alkylation and reconstituted in GluC reaction buffer (50mM Tris-HCl, 0.5 mM Glu-Glu). GluC (New England Biolabs Cat# P8100S) was reconstituted by the addition of 50 µL Millipore water and added to the sample solution at a ratio of 20:1 enzyme:protein and incubated overnight at 25°C.

Digested sample solutions were lyophilized and reconstituted in 0.1% formic acid for LCMS/MS analysis. The digested peptides were loaded onto a 150 µm ID pre-column (Magic C18, Michrom Biosciences) at 4 µl min^{-1} and separated over a 75 µm ID analytical column packed into an emitter tip containing the same packing material. The peptides were eluted over 60 min. at 300 nl min^{-1} using a

0 to 40% acetonitrile gradient in 0.1% formic acid using an EASY n-LC nano-chromatography pump (Proxeon Biosystems, Odense Denmark). The peptides were eluted into an LTQ-Orbitrap hybrid mass spectrometer (Thermo-Fisher, Bremen, Germany) operated in a data dependent mode. MS was acquired at 60,000 FWHM resolution in the FTMS and MS/MS was carried out in the linear ion trap. 6 MS/MS scans were obtained per MS cycle. The raw data were searched using Mascot 2.3.02 (Matrix Sciences, London UK). The search result was analyzed using Scaffold 3.4.3 (Proteome Software Inc, Portland, US).

S5) Kinetic analysis and interpretation of growth of Ddx4^{YFP} organelles in the HeLa nucleus

Ddx4^{YFP} was transfected into HeLa cells 24 hours prior to live cell imaging, A brightly fluorescing cell, containing only diffuse Ddx4^{YFP}, was followed over time as described above. After a period of approximately 1000 s, droplets were observed to form within the nucleus and grow over time (see Supplementary Video M1, Supplementary Figure S3). A relatively small number of droplets were observed to appear in any given instant, rather than many appearing simultaensouly, suggesting that the growth mechanism was one of nucleated growth rather than spinodal decomposition or secondary nucleation.

In order to test this, we followed the volumes of individual droplets within a single HeLa cell nucleus as a function of time. As described in the text, the total volume occupied by droplets was followed as a function of time (Figure 1D). The resulting values were well approximated by the Johnson-Mehl-Avrami-Komogorov (JMAK) equation (Avrami, 1939; Johnson and Mehl, 1939; Kolmogorov, 1937) (Figure 1D), commonly used to describe nucleated phenomena:

$$V(t) = V_T\left(1 - e^{-gt^a}\right) \qquad \text{[S4]}$$

where a is termed the Avrami exponent, g is a characteristic rate and V_T is the total droplet volume that the system will tend towards at long times. The data were found to be well described by this theory, yielding an Avrami exponent of a = 1.01 ± 0.01, g = 3.7 ± 0. 1 x 10^{-4} s^{-1} and V_T = 42.8 ± 0.1 μm^3 (Figure 1D). In the case of spherical droplets growing, the Avrami coefficient would be expected to be on the order 4. The lower value observed here is consistent with a model where the quantity of free material is limiting.

To test this, we sought to derive expressions to describe the growth of the ensemble of droplets. Following the approach of Avrami, we note that the total volume of the system $V=V_F+V_T$, the sum of droplet volume V_T and free volume V_F. In terms of concentrations, the total moles of Ddx4 is given by $N = C_0V$ where C_0 is the initial concentration of free protein. In addition, $N_T = C_TV_T$, where C_T is the concentration of protein inside a drop, a constant related to the droplet density $\rho = M_WC_T$ and M_W is

the molar mass of the constituent molecule. Finally, the concentration of free protein will be $N_F = C_F V_F$. Through conservation of mass, the total number of moles will be $C_0 V = C_F V_F + C_T V_T$, making the approximation that the system is closed. As droplets form, the concentration of free monomers will fall, as will the volume of available space, as the droplet volume increases. The concentration of proteins in the droplets, the total volume available to a droplet and the initial available concentration will be constants. Following the lead of Avrami, the increase in the number of moles in the droplets will be equal to the product of the increase in infinite dilution multiplied by the number of free moles $dN_T = dN_C(N - N_T)$. Dividing by N, we can express the quantities in terms of mole fractions, x such that $dx = dx_C(1 - x)$.

If we make the assumption that the maximum rate of formation of droplet is linear in time such that $dx_C = kdt$, and if we integrate noting that $x(0)=0$ then we obtain the result in an exponential form $x(t) = (1 - e^{-kt})$. Expanding the mole fraction, we arrive at our final result for the growth of an individual droplet:

$$V_D = \frac{C_0}{C_D} V_T \left(1 - e^{-kt}\right) = \frac{C_0 M_W}{\rho} V_T = V_D^* \left(1 - e^{-kt}\right) \qquad [S5]$$

Thus each drop will be expected to expand until either the concentration of source material is depleted or it coalesces with another droplet from an adjacent volume element, leading to a steady-state volume of V_D^* for the droplet in question. In the experiments in the cell, the concentration of droplets was relatively low and few coalescence events were observed. Nucleation events are random, and so if droplet appearance and growth occurs at a nucleation time t' relative to the observed time t, then the final expression for growth of each droplet becomes:

$$V(t,t') = V_0(t')\left(1 - e^{-k(t-t')}\right) \qquad [S6]$$

where k is the characteristic growth rate and $V_0(t')$ is the steady-state volume. This equation was found to well describe the data (Supplementary Figure S3B) with all droplets having a similar growth rates (Supplementary Figure S3E).

Three constants, the total available volume, the initial free protein concentration and the protein concentration inside the droplets specify the steady-state volume. Empirically, the steady-state volume V_D^* was found to decrease exponentially with time (Supplementary Figure S3C), suggesting that the free protein concentration varies between successively nucleated droplets such that $C_F = C_0 e^{-k_F t}$, consistent with the falling concentration of free $Ddx4^{YFP}$ that would be expected to accompany droplet growth.

The number of individual droplets was found to increase hyperbolically with time. Such a situation would be expected for a nucleated process where the limiting step is the unimolecular conversion of

a monomer to an unfavourable conformation. If nucleus formation is reversible, and that the concentration of available nucleation sites is proportional to the excess concentration C_{ex} such that nucleus formation can only happen above a threshold, then

$$\frac{dC_{ex}}{dt} = -k_N^+ C_{ex} + k_N^- N \quad [S7]$$

The excess concentration is given by $C_{ex}(t) = (C_{ex}^0 - C_{ex}^\infty)e^{-k_N t} + C_{ex}^\infty$, where $k_N = k_N^+ + k_N^-$ and so

$$N = N_0(1 - e^{-k_N t}) \quad [S8]$$

where $N_0 = k_N^+/(k_N^+ + k_N^-)$. This equation is found to be in excellent empirical accord with the data (Supplementary Figure S3D). The nucleation rate is given by the time derivative:

$$\frac{dN}{dt} = N_0 k_N e^{-k_N t} \quad [S9]$$

Taken together, we can derive an expression for the total volume of the droplets that can be compared to the Avrami equation. The total volume occupied by droplets will be given by

$$V_T = \sum N_i V_i = \int_0^t \frac{dN}{dt} V_D^*(t')\left(1 - e^{-k(t-t')}\right)dt' \quad [S10]$$

where in the second step we replace the sum with an integral over the history of all the particles to the current time. Substituting in the expression for $V_D{}^*$ and dN/dt and integrating yields

$$V_T = \frac{C_0 M_W N_0 k_N V}{\rho\left(k_N + k_F - k\right)\left(k_N + k_F\right)}\left(k\left(1 - e^{-(K_N + k_F)t}\right) + \left(k_N + k_F\right)\left(1 - e^{-kt}\right)\right) \quad [S11]$$

We note that as the values of the rates are comparable, and so this equation is essentially indistinguishable from the JMAK equation S4 with the fitting parameters obtained from the data.

Overall, the droplets appear to be in competition with each other for source material, as both the steady-state volume (Supplementary Figure S3C) and nucleation rates (Supplementary Figure S3D) decrease with time. The characteristic growth rate was found to be largely independent of time, as expected for nucleated growth (Supplementary Figure S3E). We conclude that the formation of $Ddx4^{YFP}$ organelles follow kinetics expected of a primary nucleation mechanism and that the expansion of the droplets is limited by the quantity of free $Ddx4^{YFP}$. In the cell, it is likely that the total concentration will be time dependent, reflecting factors such as translation rates.

S6) *In vitro* measurements of T_P

Experimental arrangement

Recombinant $Ddx4^{N1}$ and $Ddx4^{N1}$Me binodal phase transition points were measured using a THMS600 thermal stage controlled by a CI94 control unit and LinkSys32 software. The THMS600 unit was mounted on an Olympus BX61 upright microscope equipped with a Hamamatsu 1394 ORCA-ERA camera. Microscope hardware and image acquisition protocols were controlled using Volocity software. Samples were observed in bright field with a 10x (NA 0.4) air immersion objective.

The THMS600 thermal stage was cooled using nitrogen gas flowing through a 10ft copper (refrigerator) coil immersed in a box of dry ice coupled to and from the nitrogen supply by thick Tygon tubing. Sample chambers were constructed using two 18 mm diameter coverslips (#1; 0.16-0.19 mm thick) sandwiching a SecureSeal imaging spacer (Sigma, 9 mm internal diameter) containing 9 µL protein sample (Supplementary Figure S4A). Sealed sample chambers were positioned in the center of the THMS600 silver heating block and initially held at 50°C for 1 minute followed by a linear cooling ramp of 2°C min^{-1} to 10°C. For samples where the phase transition temperature occurred above 40°C, the initial holding temperature was 65°C for 1 minute. For transitions below 18°C the final temperature was set to 5°C.

Stacks of 25 Z-slices (10 µm step size, 4 ms exposure time per slice) aligned in Z with respect to the middle of the sample chamber were captured every 10 seconds using Olympus Focus Drive hardware. Images were acquired at bin2 (672 x 512 pixels) with a 12 bit camera chip sensitivity (0-4095 range in pixel intensity). Image stacks were processed and analyzed with Volocity software. Initial image stacks were 672 x 512 x 25 pixels (i.e. ± 120 µm in Z from the center of the sample chamber). Stacks were cropped to 120 µm thick (13 Z-slices) to correspond to the approximate thickness of the imaging spacer (and hence internal height of the sample chamber). A region of 191 x 183 x 13 pixels free of dust/dirt was then selected for further analysis. The mean and standard deviation in pixel intensity was calculated for each 191 x 183 x 13 pixel data set.

Determination of the Phase Transition (T_p) boundary

A linear baseline of the standard deviation of pixel intensity was taken from the pre-transition hold temperature (typically 50°C). The phase transition temperature for each run was taken as the point where the pixel intensity deviated from the linear baseline by more than three times the standard error of the regression estimate at a 95% confidence level (Supplementary Figure S4D):

$$S_{yo}^{2} = S_{y}^{2}\left\{1+\frac{1}{N}+\frac{(x_0-\bar{x})^2}{S_{xx}}\right\} \quad [S12]$$

In cases where the baseline displayed curvature, a smaller region in the XY plane (71 x 60 x 13 pixels) was selected for analysis. Selecting a smaller area was found not to lower the accuracy of determination of T_p.

Analysis and interpretation of T_P phase diagram

The free energy change on two separate species mixing, according to Flory-Huggins theory is given by:

$$\Delta G_{mix} = G_{mixed} - G_{unmixed} = RT\left(n_1 \ln\phi_1 + n_2 \ln\phi_2 + n_1\phi_2 \frac{\chi}{R} \right) \quad [S13]$$

Where R is the gas constant, T is the thermodynamic temperature, Φ are the volume fractions of species 1 and 2 and χ, the Flory-Huggins interaction constant reflects additional interactions between the two species. The logarithmic terms evaluate the change in translational entropy of the two species from a lattice model. The volume fraction is given by:

$$\phi_1 = \frac{n_1 N_1}{n_1 N_1 + n_2 N_2} \quad [S14]$$

Where n_i is the number of molecules and N_i is a measure of the size of species *i* and $\Phi_1+\Phi_2=1$. We can express the volume fraction in terms of the protein and free water concentrations as $\phi_1 = N_1[Ddx4]/[H_2O]$, where $1/[H_2O] = V_1 N_A$ where N_A is Avogadro's constant and V_1 is the volume of a single water molecule. In what follows, water is species 2 and $N_2=1$, and Ddx4 is species 1, and so $N_1=236$, the number of residues in $Ddx4^{N1}$.

For polymers characterized by an upper critical solution temperature (UCST), at very low temperatures, the condensed phase is favoured at all solution compositions. As the temperature is increased, a spinodal point is reached where the condensed phase is no longer the most stable. Above this temperature, the condensed phase co-exists with a mixed state. As temperature is raised further, a binodal point is reached, above which the dispersed state is the most stable. Just below this temperature, condensed droplets are expected to appear in solution, leading to this temperature originally termed the 'cloud point'. This point is determined experimentally in our work, and a suitable expression will be derived. On mixing, two phases are created. When a small amount of material 1, *dv*, is allowed to pass from phase B to phase A, the free energy of both are affected such that:

$$dG_{rxn} = \left(\frac{dG^{A}_{mix}}{dn_1} \right)_{n_2} dv - \left(\frac{dG^{B}_{mix}}{dn_1} \right)_{n_2} dv \quad [S15]$$

The condition for equilibrium is that $dG_{rxn}/dv = 0$. Noting that the chemical potential of component *i* is $\Delta\mu_i = \left(d\Delta G / dn_i \right)_j$, it follows that the equilibrium condition is given by the twin conditions $\Delta\mu_1^A = \Delta\mu_1^B$, and $\Delta\mu_2^A = \Delta\mu_2^B$. The chemical potential of species 1 and 2 are obtained from equations S13 and S14:

$$\frac{\Delta\mu_1}{RT} = \ln\phi_1 + \phi_2\left(1 - \frac{N_1}{N_2} \right) + \frac{\chi}{R} N_1 \phi_2^2 \quad [S16]$$

$$\frac{\Delta\mu_2}{RT} = \ln\phi_2 + \phi_1\left(1 - \frac{N_2}{N_1}\right) + \frac{\chi}{R}N_2\phi_1^2 \qquad \text{[S17]}$$

These can both be expressed in terms of a single parameter *Φ*, the volume fraction of species 1. The equilibrium condition for a given value of chi is characterized by the mole fractions of species 1 in the two phases, Φ_A and Φ_B. In the case of N_1=N_2, a closed form expression can be derived. When this is not the case the equations can be solved numerically. In the case of dilute polymer solutions, the Flory-Huggins interaction parameter χ will be expected to be independent of Φ and contain enthalpic and entropic terms:

$$\chi = \frac{\Delta G}{T} = \frac{\Delta H}{T} - \Delta S \qquad \text{[S18]}$$

Where *ΔH* and *ΔS* are the residual temperature independent entropy and enthalpy associated with the changing interactions that accompany the phase transition, and *T* is the thermodynamic temperature. Numerically solving equation equations S16 and S17 subject to the equilibrium conditions for a given value of *ΔH* and *ΔS* enables T_P, the temperature for equilibrium to be determined.

The calculated curves for T_P varying with Ddx4 concentration were found to be in excellent agreement with the experimental (Figure 4A). Numerical fitting of the equation enables the interaction parameters, *ΔS* and *ΔH* to be determined (Figure 4B and C). It is interesting to note that the agreement between the model and the data, and the scaling of *ΔH* and *ΔS* (Figure 4B and C) is not a function of the constants $[H_2O]$ and N_1. The specific values obtained for *ΔH* and *ΔS* however do have some dependence on the choice of these two values.

Variance of entropy and enthalpy interaction parameters with salt concentration

Both enthalpy and entropy parameters were found to vary significantly with salt concentration (Figure 4B and C). In the condensed phase, we would expect a combination of both ionic and non-ionic interactions between chains. Increasing the salt concentration would be expected to screen the ionic component according to the following potential for two single charge groups:

$$U = \frac{e^2 N_a}{4\pi\epsilon_0\epsilon_r r}e^{-r/r_d} \qquad \text{[S19]}$$

where *e* is the charge on the electron, N_a is Avogadro's constant, ε_0 is the permittivity of free space, ε_r is the relative permittivity, or dielectric of the medium, *r* is the equilibrium spacing, r_d is the Debye or interaction length given by $r_d = \sqrt{\epsilon_0\epsilon_r RT/\left(F^2 I\right)}$, and *I* is the concentration dependent part of ionic strength, $I = [NaCl]$.

Taken together, the enthalpy would be expected to scale as $\Delta H = \Delta H_0 + U$. This model gives an excellent description of the enthalpy parameter (Figure 4B), revealing a relative permittivity within the condense phase of 45 ± 13, an interaction spacing of 13 ± 2 Å, and a charge independent contribution ΔH_0 that is close to zero, -0.058 ± 0.137 kJ mol^{-1}. At low salt (50mM) the contribution to the enthalpy is predominantly ionic (U = 1 kJ mol^{-1}). At 300 mM salt, this interaction is almost entirely screened, and there is no longer an enthalpic preference for the chains to remain within droplets.

The entropic contribution to the interaction parameter was also observed to have a relatively weak dependence on salt, with a relatively modest 7% difference between the highest and lowest values (Figure 4C). Empirically, the scaling can be described by:

$$\Delta S = \Delta S_0 + B_0 R \log[NaCl] \quad \text{[S20]}$$

Where the residual entropy ΔS_0 was determined to be -6.9 ± 0.1 J mol^{-1} K^{-1} and the coefficient B_0 was -0.128 ± 0.007 J mol^{-1} K^{-1}. The form of this scaling is interesting. At higher salt concentrations, the excess entropy is more negative, indicating that the condensed phase is favoured. The hydrodynamic radius of free Ddx4 was not observed to vary with increasing salt concentration, suggesting that in addition to having weaker interactions between chains, they also become more mobile.

Phase transition propensity

The effect of ionic strength on the phase transition propensity of Ddx4^{N1}F in comparison to Ddx4^{N1} was investigated. Samples of 400 µM Ddx4^{N1}F and Ddx4^{N1} were prepared in buffers containing 50mM Tris pH 8.0, 300mM NaCl, and 2mM DTT. In order to induce phase separation, 20 µL samples were then rapidly diluted into buffers with 300, 200, 150, and 100mM NaCl with a final protein concentration of 140 µM and allowed to equilibrate for 24 h. The samples were then centrifuged at 16,000 rpm for 10 min, and 2 µL of the supernatant was taken for SDS-PAGE gel electrophoresis. Band intensities were quantified using Image Lab software (Bio-Rad) and normalised to a control sample containing 400 µM protein, 50mM Tris pH 8.0, 300mM NaCl and 2mM DTT that was rapidly diluted to 140 µM protein, 50mM Tris pH 8.0, 300mM NaCl and 2mM DTT and a sample immediately taken for SDS-PAGE gel electrophoresis.

S7) Statistical analysis of the sequence features that give rise to organelle formation

Datasets

As a training set we used all proteins in metazoans that were orthologous to Ddx4. Specifically we extracted from eggNOG (Powell et al., 2012) the orthologous group meNOG12075 (DEAD (Asp-Glu-

Ala-Asp) box polypeptide 4) comprising 68 proteins from 46 species. As a background set we used all the sequences in the proteomes of these 46 species. When applying our strategy to discover new proteins with features similar to Ddx4 and to assess whether these include proteins with the potential to form biologically relevant non-membrane-bound organelles we use the human proteome extracted from UniprotKB (The UniProt Consortium, 2012) and redundancy reduced at 40% identity level using cd-hit (Li and Godzik, 2006). We used IUPred (Dosztanyi et al., 2005) (cutoff of 0.3, setting long, in order to exclude disordered regions shorter than 20-30 residues long, and structural domain regions, while still including as many of the disordered regions as possible) to extract the disordered regions of the sequences in these proteins. All the following analyses were performed on the disordered regions of these datasets.

Sliding window method for calculating net charge index

In order to investigate patterns of charged residues within protein sequences, we define the "net charge" index, I, within a 10 residue window to be $I = RK - ED$ where RK and ED is the ratio of the counts of the specified residues within a 10 residue window to that in all IDP regions in the human proteome (Figure 6A).

Calculation of residue pair preferences

The frequency of all observed pairs in the disordered regions of our positive dataset (meNOG12075) is calculated as the 'observed' frequency, and our background set as the 'expected' frequency. We then normalise these frequencies, using the probability of observing these residue pairs by random chance, based on the set's sequence amino acid composition. Finally we calculate the significance of the observed values, as the ratio of the observed normalised frequencies compared to the expected ones. This is represented in Supplementary Figure S6B, where the size of the dots represents the normalised frequency of amino acid pairs in the disordered regions of the Ddx4 human protein and the colour represents the significance of observation of the particular pair in the Ddx4 orthologous set.

Similarity 'fingerprint' generation

The inter-residue spacing of all possible dipeptides was calculated. We scanned each sequence and upon an occurrence of our residue of interest e.g. R flanked by G, we calculated the frequency of observing the next such residues in each distance from 1 to 30. The frequency was then normalised by the probability of observing this pair by chance. The resulting matrices were divided by similar matrices derived from the background set. These represent the profiles of blocks containing our residue pairs of interest in the Ddx4 orthologs and how these differ compared to the background set (Supplementary Figure 6C).

Sequence scanning

We used the matrices generated from the [FR]G blocks, the RG and the FG blocks with the hypothesis that these features of Ddx4 will lead to the identification to more similar proteins that are also able to form phase separations or gel-like structures within the cell. For each sequence we used a sliding window of length 30 and scored them based on the matrices generated previously, keeping only the hits with score >0. We performed this on the human proteome, after we reduced its redundancy by 40% using cd-hit in order to avoid bias when studying their functional enrichment (Li and Godzik, 2006). We then look at the hits with at least one hit with score greater than 2 to see the over-represented GO terms. We further scanned in a similar fashion (after extracting only the disordered regions) the Baker's yeast (*Saccharomyces cerevisiae*) proteome and the *E. coli* proteome (strain K12).

Functional enrichment analysis

Functional enrichment analyses were performed using DAVID and applying and EASE score cutoff of 0.05 to the results. (Da Wei Huang and Lempicki, 2008; Sherman and Lempicki, 2009). The results were represented using the 'Enrichment Map' plugin in Cytoscape and a p-value cutoff of 0.05, an overlap coefficient of 0.6 and a FDR Q-value of 0.1 (Shannon et al., 2003).

S8) DNA uptake into organelles (nucleic acid partitioning)

Organelles were prepared as for the hanging drop experiments by mixing 1.5 µl $Ddx4^{N1}$ protein (325 µM in 300 mM NaCl buffer) with 1.5 µl of a 0 mM NaCl buffer containing either no DNA or 2 µM total ssDNA or dsDNA, such that the salt dilution resulted in immediate organelle formation and a final concentration of 1 µM total fluorophore per sample. DIC and laser confocal images were obtained using an Olympus IX81 inverted microscope equipped with a 30x (NA 1.05) silicon immersion objective. Fluorescence excitation was achieved with a 635nm laser. Fluorescence emission between 650 – 750 nm was collected using the same objective. Stacks of 23 Z-slices (each 1024x1024 pixels, acquired with 4 µs $pixel^{-1}$ scanning speed, 1 µm step size, 1% laser power) centered in Z with respect to the middle of the sample were captured for 3 fields of view containing $Ddx4^{N1}$ droplets. Microscope hardware and image acquisition was controlled with FLUOVIEW software. Image analysis was performed with Volocity software. Inspection of Z-stacks revealed that the samples were 9 µm thick and were therefore initially cropped in Z to 9 slices before further analysis. Next, 60 regions of interest (ROIs; 10 inside $Ddx4^{N1}$ droplets and 10 outside $Ddx4^{N1}$ droplets per field of view) were selected and the total emission, *E*, was computed and normalised by ROI volume (Supplementary Figure S7A). Residual average fluorescence from the sample containing no DNA was then subtracted from the ssDNA and dsDNA-containing samples and the average and standard deviation (overbar) in total fluorescence calculated. The partition coefficient was defined as:

$$K_{partition} = \frac{[DNA]^{outside}}{[DNA]^{inside}} = \frac{\overline{E}^{outside}_{+DNA} - \overline{E}^{outside}_{empty}}{\overline{E}^{inside}_{+DNA} - \overline{E}^{inside}_{empty}} \quad [S21]$$

The partition coefficient was converted into a free energy via $\Delta G_{partition} = -RTlnK_{partition}$.

Supplementary references

Ahmed, K., Li, R., and Bazett-Jones, D.P. (2008). Electron spectroscopic imaging of the nuclear landscape. In The Nucleus (Springer), pp. 415-423.

Avrami, M. (1939). Kinetics of phase change. I. General theory. J. Chem. Phys. *7*, 1103.

Da Wei Huang, B.T.S., and Lempicki, R.A. (2008). Systematic and integrative analysis of large gene lists using DAVID bioinformatics resources. Nat. Protoc. *4*, 44-57.

Delaglio, F., Grzesiek, S., Vuister, G.W., Zhu, G., Pfeifer, J., and Bax, A. (1995). NMRPipe: a multidimensional spectral processing system based on UNIX pipes. J. Biomol. NMR *6*, 277-293.

Dosztanyi, Z., Csizmok, V., Tompa, P., and Simon, I. (2005). The pairwise energy content estimated from amino acid composition discriminates between folded and intrinsically unstructured proteins. J. Mol. Biol. *347*, 827-839.

Dundr, M., and Misteli, T. (2010). Biogenesis of nuclear bodies. Cold Spring Harb Perspect Biol *2*, a000711.

Goddard, T., and Kneller, D. (2004). SPARKY 3. University of California, San Francisco.

Johnson, W.A., and Mehl, R.F. (1939). Reaction kinetics in processes of nucleation and growth. Trans. Aime *135*, 396-415.

Kolmogorov, A.N. (1937). On the statistical theory of the crystallization of metals. Bull. Acad. Sci. USSR, Math. Ser *1*, 355-359.

Li, W., and Godzik, A. (2006). Cd-hit: a fast program for clustering and comparing large sets of protein or nucleotide sequences. Bioinformatics *22*, 1658-1659.

Powell, S., Szklarczyk, D., Trachana, K., Roth, A., Kuhn, M., Muller, J., Arnold, R., Rattei, T., Letunic, I., and Doerks, T. (2012). eggNOG v3. 0: orthologous groups covering 1133 organisms at 41 different taxonomic ranges. Nucleic Acids Res. *40*, D284-D289.

Shannon, P., Markiel, A., Ozier, O., Baliga, N.S., Wang, J.T., Ramage, D., Amin, N., Schwikowski, B., and Ideker, T. (2003). Cytoscape: a software environment for integrated models of biomolecular interaction networks. Genome Res. *13*, 2498-2504.

Sherman, B.T., and Lempicki, R.A. (2009). Bioinformatics enrichment tools: paths toward the comprehensive functional analysis of large gene lists. Nucleic Acids Res. *37*, 1-13.

The UniProt Consortium (2012). Reorganizing the protein space at the Universal Protein Resource (UniProt). Nucleic Acids Res. *40*, D71-75.

Theillet, F.X., Smet-Nocca, C., Liokatis, S., Thongwichian, R., Kosten, J., Yoon, M.K., Kriwacki, R.W., Landrieu, I., Lippens, G., and Selenko, P. (2012). Cell signaling, post-translational protein modifications and NMR spectroscopy. J Biomol NMR *54*, 217-236.

Wilkins, D.K., Grimshaw, S.B., Receveur, V., Dobson, C.M., Jones, J.A., and Smith, L.J. (1999). Hydrodynamic radii of native and denatured proteins measured by pulse field gradient NMR techniques. Biochemistry *38*, 16424-16431.
